# 4-Oxypiperidine Ethers as Multiple Targeting Ligands at Histamine H_3_ Receptors and Cholinesterases

**DOI:** 10.1021/acschemneuro.3c00800

**Published:** 2024-03-05

**Authors:** Beata Michalska, Marek Dzięgielewski, Justyna Godyń, Tobias Werner, Marek Bajda, Tadeusz Karcz, Katarzyna Szczepańska, Holger Stark, Anna Więckowska, Krzysztof Walczyński, Marek Staszewski

**Affiliations:** † Department of Synthesis and Technology of Drugs, Medical University of Lodz, Muszynskiego 1, 90-151 Lodz, Poland; ‡ Department of Physicochemical Drug Analysis, Jagiellonian University Medical College, Medyczna 9, 30-688 Krakow, Poland; § Institute of Pharmaceutical and Medicinal Chemistry, Heinrich Heine University Düsseldorf, Universitaetsstr. 1, 40225 Duesseldorf, Germany; ∥ Department of Technology and Biotechnology of Drugs, Faculty of Pharmacy, Jagiellonian University Medical College, Medyczna 9, 30-688 Krakow, Poland; ⊥ Department of Medicinal Chemistry, Maj Institute of Pharmacology, Polish Academy of Sciences, Smetna 12, 31-343 Krakow, Poland

**Keywords:** histamine H_3_ receptor, acetylcholinestrase inhibitor, butyrylcholinesterase inhibitor, multiple targeting, polypharmacology, neurodegenerative disease

## Abstract

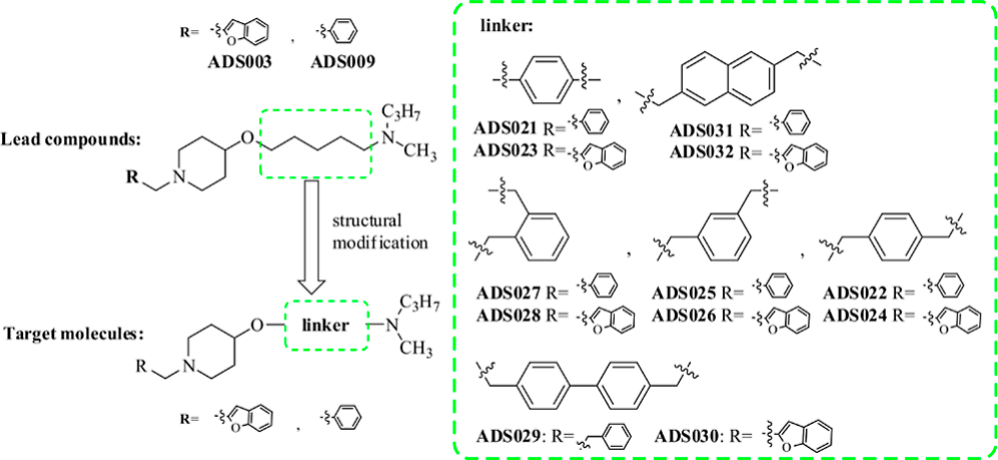

This study examines the properties of a novel series of 4-oxypiperidines designed and synthesized as histamine H_3_R antagonists/inverse agonists based on the structural modification of two lead compounds, viz., **ADS003** and **ADS009**. The products are intended to maintain a high affinity for H_3_R while simultaneously inhibiting AChE or/and BuChE enzymes. Selected compounds were subjected to *h*H_3_R radioligand displacement and *gp*H_3_R functional assays. Some of the compounds showed nanomolar affinity. The most promising compound in the naphthalene series was **ADS031**, which contained a benzyl moiety at position 1 of the piperidine ring and displayed 12.5 nM affinity at the *h*H_3_R and the highest inhibitory activity against AChE (IC_50_ = 1.537 μM). Eight compounds showed over 60% *eq*BuChE inhibition and hence were qualified for the determination of the IC_50_ value at *eq*BuChE; their values ranged from 0.559 to 2.655 μM. Therapy based on a multitarget-directed ligand combining H_3_R antagonism with additional AChE/BuChE inhibitory properties might improve cognitive functions in multifactorial Alzheimer’s disease.

## 1 Introduction

It has been more than 40 years since the seminal study by Arrang and co-workers, which demonstrated that histamine inhibits its own synthesis and release from cortical slices of the rat brain via a new histamine receptor that is pharmacologically distinct from both the histamine H_1_ and H_2_ receptors.^1^ The histamine H_3_ receptor (H_3_R) was described as a presynaptic autoreceptor involved in the inhibition of histamine secretion in the brain and proved the neurotransmitter function of histamine.^2^

It was later discovered that H_3_Rs do not only control histamine release but also, functioning as heteroreceptors, modulate the other neurotransmitter systems,^3^ e.g., the cholinergic,^4,5^ dopaminergic,^6^ noradrenergic,^7,8^ and serotoninergic^9^ systems, in both the central and peripheral nervous systems. H_3_R antagonists/inverse agonists demonstrated diverse pharmacological effects in preclinical and clinical studies, highlighting the importance of various central nervous system (CNS)-related therapeutic applications, including Parkinson’s disease and Alzheimer’s disease (AD),^10^ narcolepsy,^11^ obesity,^12^ dementia, epilepsy, schizophrenia,^13^ and attention deficit hyperactivity disorder (ADHD).^14^ Recently, many other CNS-related disorders have also been suggested as candidates for treatment by H_3_R antagonist/inverse agonist ligands: Tourette syndrome,^15^ Prader–Willi syndrome,^16^ depression,^17^ or autism.^18^

The most commonly prescribed drugs for the treatment of AD are cholinesterase inhibitors, which improve memory function and delay cognitive decline without altering the underlying pathology.^19^ However, these drugs show clear limitations concerning both efficacy and tolerability. Their efficacy on cognition enhancement is limited, with the preferred target patient population suffering mild to moderate cognitive impairment.^19^ From a neurochemical point of view, the decline in cognitive function associated with normal aging, mild cognitive impairment, AD, and related conditions is thought to be caused by a decrease in cortical cholinergic neurotransmission. Such neurotransmission can be improved and enhanced by both AChE/BuChE inhibitors and H_3_R antagonists, mediated by different neuronal mechanisms. For instance, the learning and memory-facilitating potential of plentiful H_3_R antagonists have been shown. The etiology stems from their wake-promoting properties in preclinical and clinical studies.^20−22^ The arousal effect is cumulated with enhanced cortical fast rhythms that correspond to higher brain functions, including alertness, attention, and cognition.^21,22^ Moreover, H_3_R antagonists increase the synthesis and release of histamine and its associated memory enhancement. Furthermore, they can increase the release of other neurotransmitters involved in cognition: the prefrontal cortex (acetylcholine, dopamine), anterior cingulate (acetylcholine, dopamine, norepinephrine), and hippocampus (acetylcholine).^23−25^ This suggests that it may be possible to enhance cholinergic neurotransmission by combining the two activities in a single molecule as a multitargeting approach.^26,27^ Histamine can control the signaling effects induced by the activation of the cholinergic receptor through the H_3_R-controlled mechanism, regulating the excitatory effects of acetylcholine.^28^ H_3_Rs, modulating the cholinergic system, increase the level of acetylcholine in the synaptic cleft. Two main enzymes hydrolyze acetylcholine: AChE and butyrylcholinesterase (BuChE). AChE is a neuronal enzyme predominantly found in the neuronal cleft of a healthy brain, and its activity decreases when AD occur.^29,30^ When AChE activity in AD is reduced, AChE and BuChE progression is inverted, denoting the importance of both enzymes in cognitive dysfunction. Dual-acting compounds, inhibiting additionally AChE or BuChE, prolong the survival time of acetylcholine in the neuronal cleft.^31,32^ Co-administration of a H_3_R antagonist (MK-3134) and an AChE inhibitor (donepezil) showed a more pronounced, pro-cognitive outcome than the effects of each compound separately.^33,34^ Dual-acting drugs can be important for the treatment of Alzheimer’s disease due to their lower toxicity, less drug–drug interactions, unified pharmacokinetic profile, and higher efficacy. By acting through H_3_R in the CNS, they lower peripheral AChE levels and may result in fewer side effects.^35^ By inhibiting AChE, these compounds may slow the β-amyloid peptide aggregation process promoted by AChE.^36^

**Figure 1:**
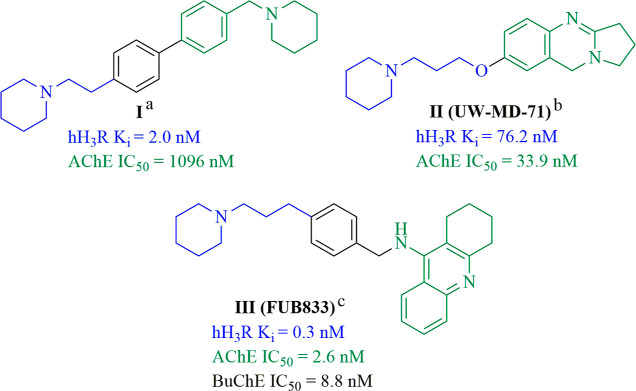
Representative structures of nonimidazole H_3_R antagonists with an AChE/BuChE inhibitory effect. (a) Data from ref (37). (b) Data from ref (38). (c) Data from ref (39).

In recent years, there has been the development of a novel class of procognitive dual-acting drugs, that exhibit both H_3_R antagonism/inverse agonism and inhibition of AChE and/or BuChE. Three such nonimidazole H_3_R antagonists with additional cholinesterase inhibition are shown in Figure 1.

**Figure 2:**
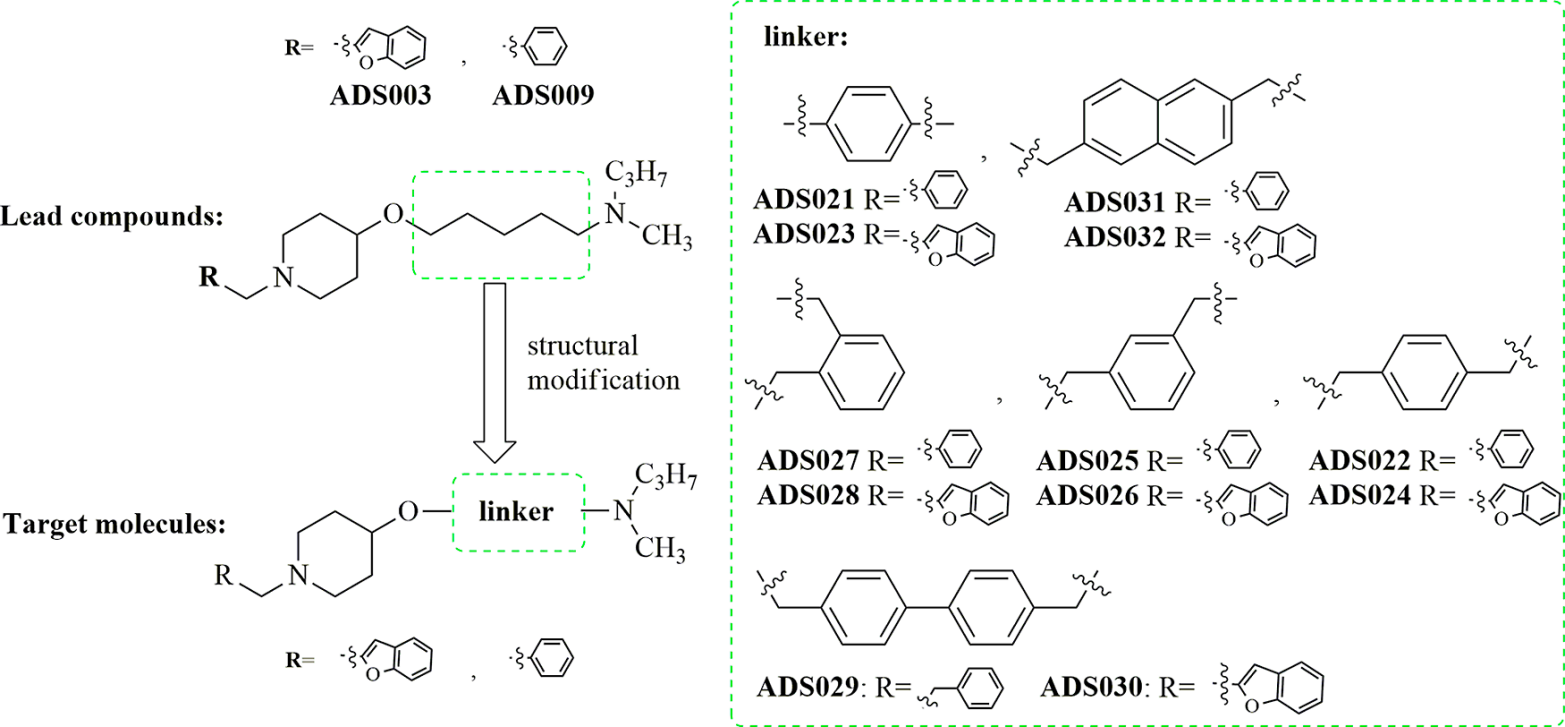
Target molecules for this study.

Previously, our laboratory has described piperazine,^40^ guanidine,^41^ and 4-hydroxpiperidine-based H_3_R antagonists.^42,43^ The structure–activity relationship (SAR) of the 4-hydroxpiperidine series found the most potent compounds to be 5-((1-(benzofuran-2-ylmethyl)piperidin-4-yl)oxy)-*N*-methyl-*N*-propylpentan-1-amine (**ADS003**) (pA_2_ = 8.47, for reference: thioperamide pA_2_ = 8.67) and 5-((1-benzylpiperidin-4-yl)oxy)-*N*-methyl-*N*-propylpentan-1-amine (**ADS009**) (pA_2_ = 7.79) (Figure 2).^42,43^ Further in vivo evaluation of the impact of **ADS003** on brain neurotransmitter systems showed its ability to cross the blood–brain barrier and potency at H_3_R similar to the reference compound, ciproxifan.^42^

## 2 Results and Discussion

### 2.1 Design

In previous studies on 4-hydroxypiperidines, the two ligands **ADS003** and **ADS009** (Figure 2) were shown to be highly potent antagonists/inverse agonists at the H_3_R. They were used as lead compounds for the following structural modification: replacement of the flexible five-methylene group chain with a benzene, biphenyl, or naphthalene linker (Figure 2). The idea to replace the flexible alkyl linker with semirigid groups of the lead compounds is consistent with studies that have demonstrated that replacement of the alkyl chain with more rigid moieties results in the formation of higher affinity H_3_R ligands.^42^ A moiety with a more restricted conformation may be better fitted to the receptor-binding site due to reduced flexibility (i.e., degrees of freedom).^44^

**Scheme 1:**
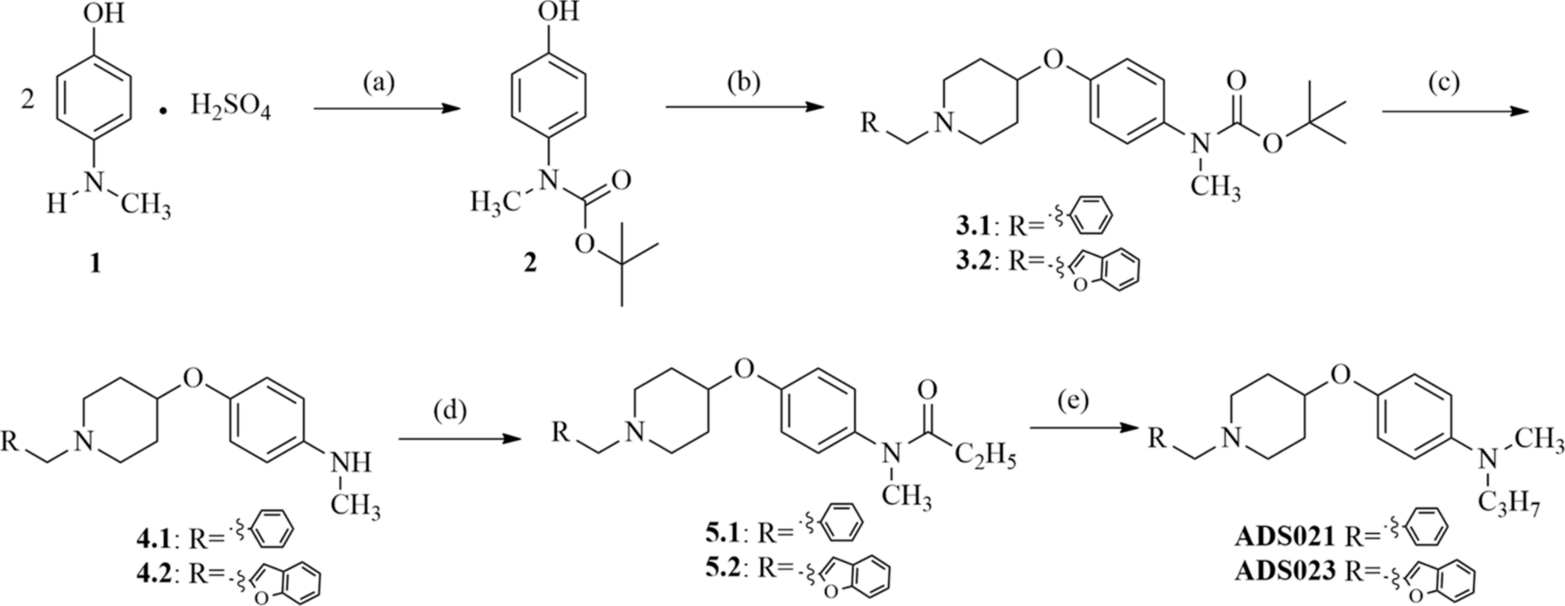
Synthesis of 4-((1-benzylpiperidin-4-yl)oxy)-*N*-methyl-*N*-propylaniline (**ADS021**) and 4-((1-(Benzofuran-2-ylmethyl)piperidin-4-yl)oxy)-*N*-methyl-*N*-propylaniline (**ADS023**) Reagents and conditions: (a) 4-(methylamino)phenol hemisulfate salt (1 equiv), di-*tert*-butyl dicarbonate (1.5 equiv), Et_3_N (2 equiv), CH_3_OH, 4 h, rt; (b) 1 (1 equiv), 1-benzyl-4-hydroxypiperidine/1-(benzofuran-2-ylmethyl)piperidin-4-ol (1.3 equiv), diethyl azodicarboxylate (1.3 equiv), triphenylphosphine (1.3 equiv), dry THF, 10 h, 0 °C; (c) **3.1**/**3.2** (1 equiv), trifluoroacetic acid (25 equiv), CH_2_Cl_2_, 12 h, rt; (d) **4.1**/**4.2** (1 equiv), propionic anhydride (2 equiv), 2 h, 0–10 °C; (e) **5.1**/**5.2** (1 equiv), LiAlH_4_ (2 equiv), Et_2_O, 2 h, reflux.

**Scheme 2:**
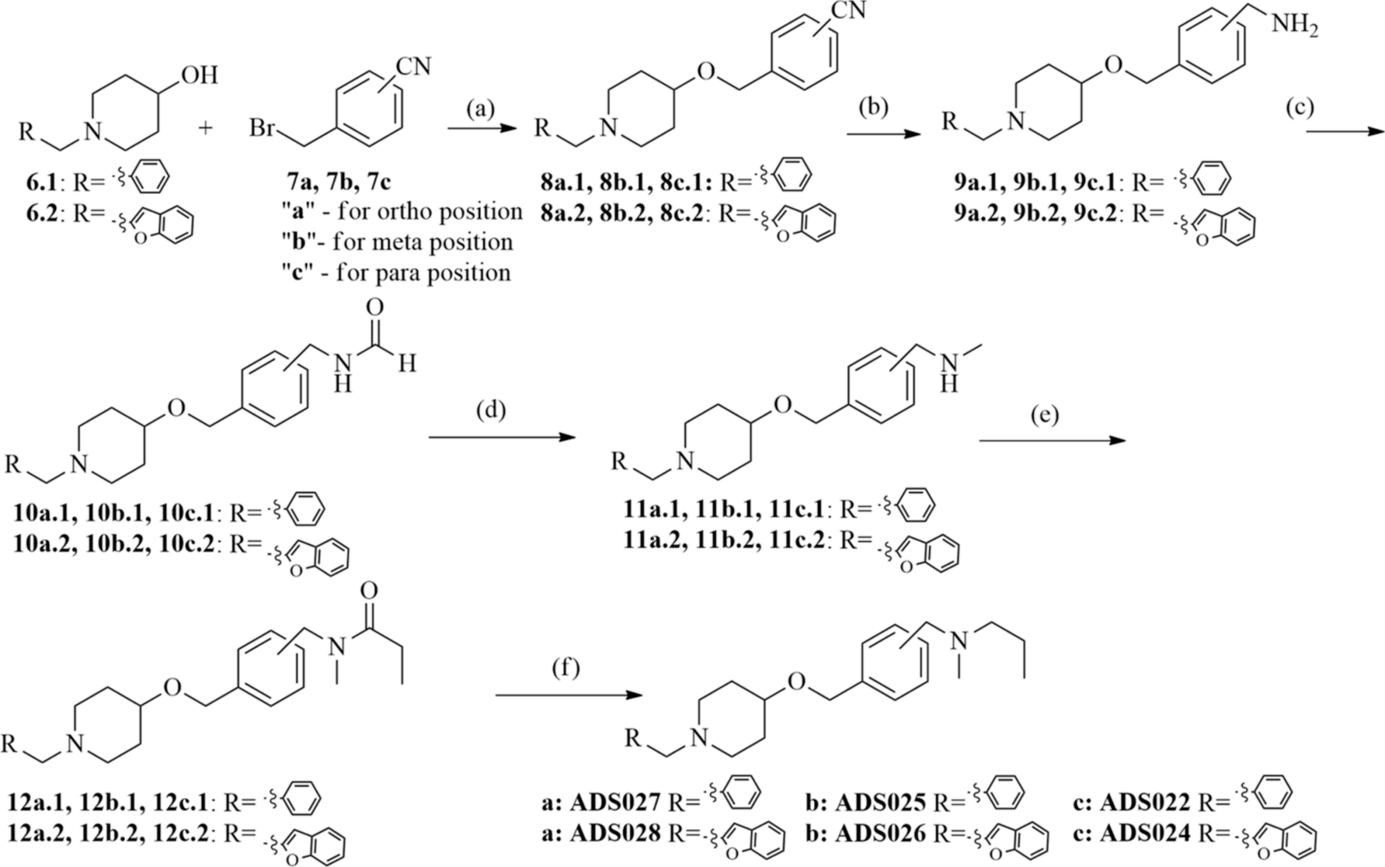
Synthesis of **ADS022**, **ADS024**, **ADS025**, **ADS026**, **ADS027**, and **ADS028** Reagents and conditions: (a) 1-benzyl-4-hydroxypiperidine/1-(benzofuran-2-ylmethyl)piperidin-4-ol (1 equiv), sodium hydride (2 equiv), 15-crown-5 ether (0.1 equiv), 4-(bromomethyl)benzonitrile (1.2 equiv), toluene, 48 h, rt; (b) **8a.1**/**8b.1**/**8c.1**/**8a.2**/**8b.2**/**8c.2** (1 equiv), LiAlH_4_ (2 equiv), Et_2_O, 3 h, reflux; (c) **9a.1**/**9b.1**/**9c.1**/9**a.2**/**9b.2**/**9c.2** (1 equiv), methyl formate (10 equiv), 10 h, rt; (d) **10a.1**/**10b.1**/**10c.1**/**10a.2**/**10b.2**/**10c.2** (1 equiv), LiAlH_4_ (2 equiv), Et_2_O, 3 h, reflux; (e) **11a.1**/**11b.1**/**11c.1**/**11a.2**/**11b.2**/**11c.2** (1 equiv), propionic anhydride (2 equiv), CH_2_Cl_2_, 10 h, 0–10 °C; (f) **12a.1**/**12b.1**/**12c.1**/**12a.2**/**12b.2**/**12c.2** (1 equiv), LiAlH_4_ (2 equiv), Et_2_O, 3 h, reflux.

**Scheme 3:**
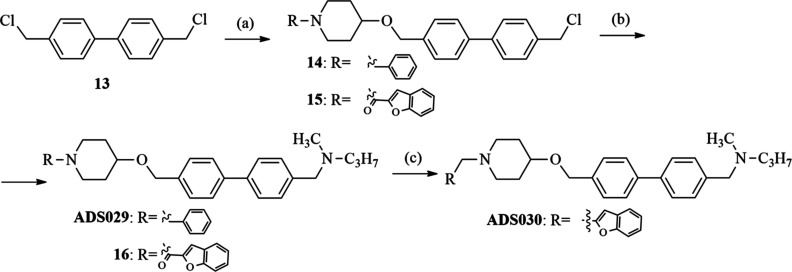
Synthesis of *N*-((4′-(((1-Benzylpiperidin-4-yl)oxy)methyl)-[1,1′-biphenyl]-4-yl)methyl)-*N*-methylpropan-1-amine (**ADS029**) and *N*-((4′-(((1-(Benzofuran-2-ylmethyl)piperidin-4-yl)oxy)methyl)-[1,1′-biphenyl]-4-yl)methyl)-*N*-methylpropan-1-amine (**ADS030**) Reagents and conditions: 4,4′-bis(chloromethyl)-1,1′-biphenyl (1.2 equiv), sodium hydride (2 equiv), 15-crown-5 ether (0.1 equiv), 1-benzyl-4-hydroxypiperidine or benzofuran-2-yl(4-hydroxypiperidin-1-yl)methanone (1 equiv), toluene, 48 h, rt; (b) **14**/**15** (1 equiv), K_2_CO_3_ (2 equiv), the *N*-methyl-*N*-propylamine was added (2 equiv), CH_3_CN, 12 h, rt; (c) **16** (1 equiv), LiAlH_4_ (2 equiv), Et_2_O, 3 h, reflux.

**Scheme 4:**
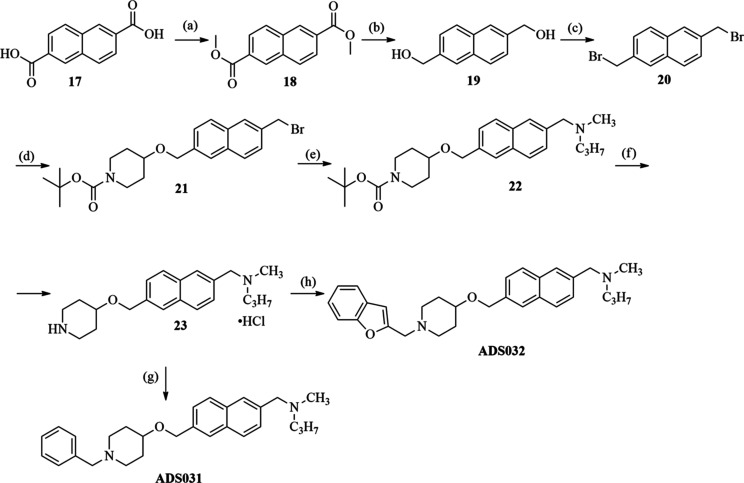
Synthesis of *N*-((6-(((1-Benzylpiperidin-4-yl)oxy)methyl)naphthalen-2-yl)methyl)-*N*-methylpropan-1-amine (**ADS031**) and *N*-((6-(((1-(Benzofuran-2-ylmethyl)piperidin-4-yl)oxy)methyl)naphthalen-2-yl)methyl)-*N*-methylpropan-1-amine (**ADS032**) Reagents and conditions: (a) **17** (1 equiv), Li_2_CO_3_ (6 equiv), CH_3_I (6 equiv), DMF_2_, 24 h, rt; (b) **18** (1 equiv), LiAlH_4_ (2 equiv), THF, 24 h, rt; (c) **19** (1 equiv), phosphorus tribromide (1 equiv), CH_2_Cl_2_, DMF, 1 h, rt; (d) **20** (1.2 equiv), *tert*-butyl 4-hydroxypiperidine-1-carboxylate (1 equiv), sodium hydride (2 equiv), 15-crown-5 ether (0.1 equiv), toluene, 48 h, rt; (e) **21** (1 equiv), K_2_CO_3_ (2 equiv), *N*-methyl-*N*-propylamine (2 equiv), acetone, 24 h, rt; (f) **22** (1 equiv), 4 N HCl in dioxane (10 equiv), CHCl_3_, 24 h, rt; (g) **23** (1 equiv) and benzaldehyde (1.3 equiv), 1,2-dichloroethane, NaBH(OAc)_3_ (5 equiv), 24 h, argon atmosphere, rt; (h) **23** (1 equiv), benzofuran-2-carbaldehyde (1.3 equiv), 1,2-dichloroethane, NaBH(OAc)_3_ (5 equiv), 12 h, argon atmosphere, rt.

In the first step, two compounds were synthesized (**ADS021**, **ADS023**; Scheme 1) in which the aliphatic linker was replaced by a benzene ring carrying an *N*-methyl-*N*-propylamine group in the para position. Following this, a series of derivatives were synthesized; these contained a methylene linker connected to the oxygen in position 4 of piperidine and a benzene ring carrying an *N*-methyl-*N*-propylaminomethyl substituent in the ortho-, meta-, or para-position (**ADS027**, **ADS028**, **ADS025**, **ADS026**, **ADS022**, and **ADS024**, respectively; Scheme 2). Finally, a series containing a biphenyl ring (**ADS029**, **ADS030**; Scheme 3) and a naphthalene ring (**ADS031**, **ADS032**; Scheme 4) in place of the aliphatic linker was prepared.

### 2.2 Chemistry

All synthetic procedures are illustrated in Schemes 1–4. To synthesize the compounds presented in all schemes, the following intermediates were prepared: 1-(benzofuran-2-ylmethyl)piperidin-4-ol, *tert*-butyl-4-hydroxypiperidine-1-carboxylate, benzofuran-2-yl(4-hydroxypiperidin-1-yl)methanone, and benzofuran-2-carbaldehyde (Supporting Information).

To obtain *tert*-butyl (4-((1-benzylpiperidin-4-yl)oxy)phenyl)(methyl)carbamate (**3.1**; Scheme 1), commercially available 4-(methylamino)phenol hemisulfate salt (**1**) was treated with di-*tert*-butyl dicarbonate in the presence of triethylamine (Scheme 1). Mitsunobu reactions of compound **2** with appropriate alcohol (1-benzyl-4-hydroxypiperidine or 1-(benzofuran-2-ylmethyl)piperidin-4-ol) in the presence of diethyl azodicarboxylate and triphenylphosphine lead to **3.1** and **3.2**, respectively. Acidic deprotection of Boc-groups gives products **4.1** and **4.2**. Amines (**4.1** and **4.2**) were treated with an excess of propionic anhydride to give amides **5.1** and **5.2**, which were subsequently reduced with LiAlH_4_ to the final products **ADS021** and **ADS023**.

Etherification of appropriate alcohol (1-benzyl-4-hydroxypiperidine **6.1** or 1-(benzofuran-2-ylmethyl)piperidin-4-ol **6.2**) with commercially available nitriles (**7a**, **7b**, **7c**) and sodium hydride as well as a catalytic amount of 15-crown-5 led to **8a.1**, **8b.1**, **8c.1**, **8a.2**, **8b.2**, and **8c.2** (Scheme 2). The amines **9a.1**, **9b.1**, **9c.1**, **9a.2**, **9b.2**, and **9c.2** were obtained from the nitriles **8a.1**, **8b.1**, **8c.1**, **8a.2**, **8b.2**, and **8c.2** by reduction with LiAlH_4_. Formylation of the amines with excess methyl formate yielded the amide derivatives **10a.1**, **10b.1**, **10c.1**, **10a.2**, **10b.2**, and **10c.2**, which were reduced with LiAlH_4_ to the secondary amines **11a.1**, **11b.1**, **11c.1**, **11a.2**, **11b.2**, and **11c.2**. The secondary amines were treated with excess propionic anhydride, giving the amide derivatives **12a.1**, **12b.1**, **12c.1**, **12a.2**, **12b.2**, and **12c.2**. The amides were reduced with LiAlH_4_ in dry ethyl ether to the corresponding products **ADS027**, **ADS028**, **ADS025**, **ADS026**, **ADS022**, and **ADS024**.

As illustrated in Scheme 3, etherification of commercially available 4,4′-bis(chloromethyl)-1,1′-biphenyl (**13**) with appropriate alcohol (1-benzyl-4-hydroxypiperidine or benzofuran-2-yl(4-hydroxypiperidin-1-yl)methanone) with sodium hydride and a catalytic amount of 15-crown-5 leads to **14** and **15**. *N*-alkylation of *N*-methyl-*N*-propylamine with **14** or **15** in the presence of potassium carbonate led to the formation of **ADS029** and **16**. The final **ADS030** was obtained by the reduction of amide (**16**) with LiAlH_4_.

To synthesize the final compounds presented in Scheme 4, commercially available naphthalene-2,6-dicarboxylic acid (**17**) was first treated with excess methyl iodide and lithium carbonate to obtain the diester derivative **18** (according to Benito and Meldal^45^). Following this, **18** was reduced with LiAlH_4_ to the relevant diol (**19**).^46^ Bromination of compound **19** led to derivative **20**,^46^ which was used as a substrate for the etherification process of *tert*-butyl 4-hydroxypiperidine-1-carboxylate. The obtained ether **21** was treated with *N*-methyl-*N*-propylamine in the presence of potassium carbonate, leading to compound **22**. Acidic deprotection of Boc-groups from the piperidine moiety yielded **23**. Finally, reductive amination of **23** and the corresponding aldehydes (benzaldehyde or benzofuran-2-carbaldehyde) in the presence of sodium triacetoxyborohydride allowed the final compounds **ADS031** and **ADS032** to be obtained.

All final **ADS** compounds were converted into their salts of oxalic or fumaric acid.

### 2.3 Pharmacology

#### 2.3.1 Ex Vivo *gp*H_3_R Screening on Isolated Guinea Pig Ileum and *h*H_3_R Radioligand Displacement Assays

All newly synthesized compounds were evaluated ex vivo as H_3_R antagonists/inverse agonists on guinea pig ileum (*gp*H_3_R) stimulated electrically for contraction.^47^ Two of the most active pairs (containing benzyl and benzofuran groups) of compounds **ADS022** and **ADS024** as well as **ADS031** and **ADS032**, all exceeded a pA_2_ of 7, were subjected to a radioligand displacement assay in membrane fractions of HEK-293 cells stably expressing human H_3_R (*h*H_3_R).^48^ The affinity for all compounds is summarized in Table 1.

**Table 1:**
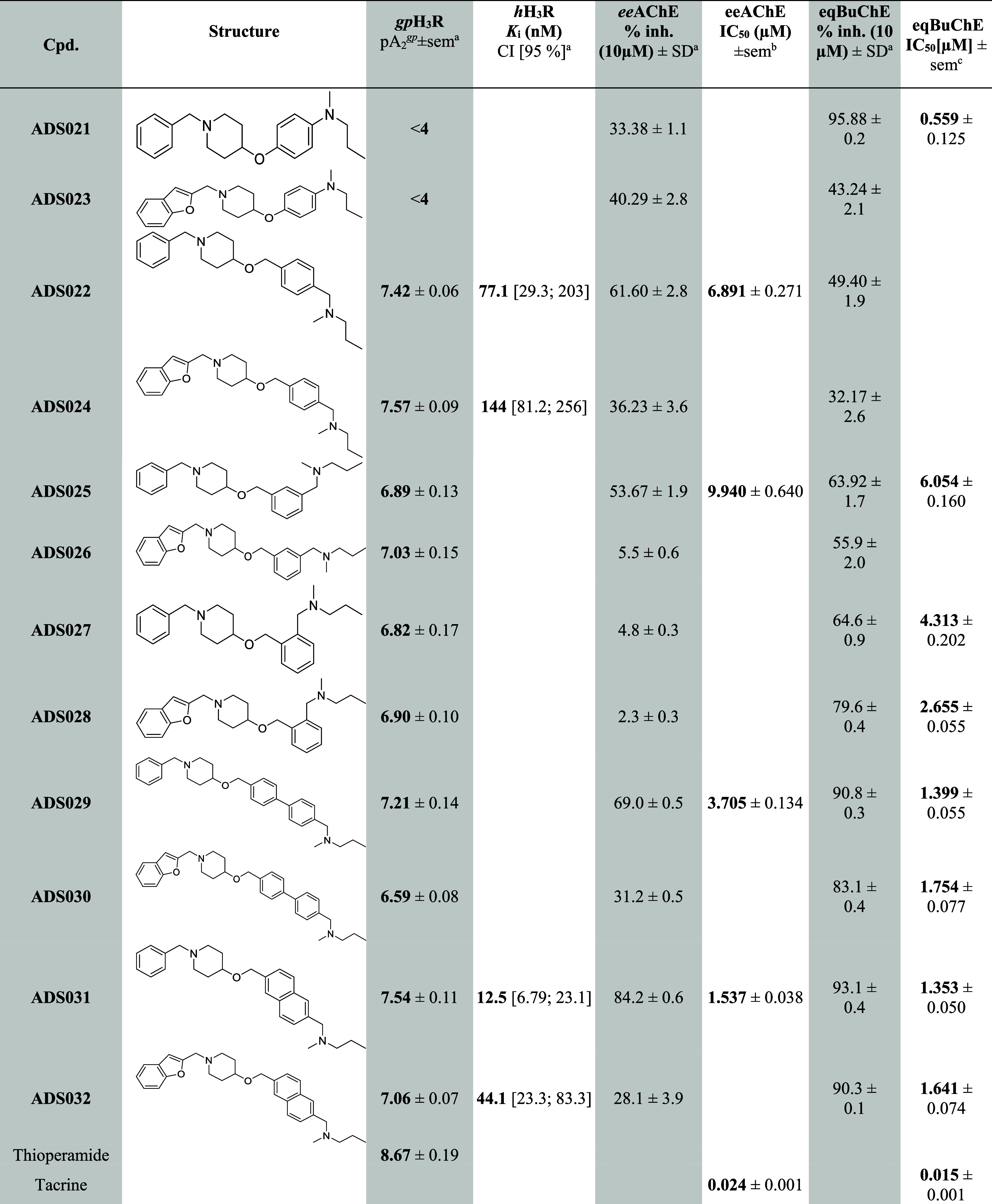
Results of ex vivo *gp*H_3_R Screening on the Isolated Guinea Pig Ileum; *h*H_3_R Radioligand Displacement Assays; Inhibition of *electric eel* AChE and *equine serum* BuChE

a pA_2_, *K*_i_, *ee*AChE%inh. and *eq*BuChE%inh. are means from at least three independent experiments.

b IC_50_ inhibitory concentration of *electric eel* AChE, mean value ± sem of triplicate independent experiments.

c IC_50_ inhibitory concentration of BuChE from *equine serum*, mean value ± sem of triplicate independent experiments. sem: standard error of the mean, *h*: human; *gp*: guinea pig.

In this series, derivatives **ADS021** and **ADS023** showed the lowest potency (pA_2_ < 4). The introduction of an additional methylene group between 4-oxypiperidine and the aromatic ring resulted in a significant increase in activity, viz., **ADS022** pA_2_ = 7.42 and **ADS024** pA_2_ = 7.57, carrying the *N*-methyl-*N*-propyl-aminomethyl substituent at position 4 of the benzene ring. Those with *N*-methyl-*N*-propyl-aminomethyl substituted at position 3 exhibited lowered activity, viz. **ADS025** (pA_2_ = 6.89) and **ADS026** (pA_2_ = 7.03). In addition, by altering the *N*-methyl-*N*-propyl-aminomethyl substituent at position 2 of the aromatic ring, synthesized compounds (**ADS027** and **ADS028**) demonstrated a further decrease in activity. By looking at the drug-likeness properties of all compounds calculated in the SwissADME web tool,^49^ we have observed that only **ADS021** of the benzyl derivatives has two H-bond acceptors, while the rest of the benzyl derivatives contain three H-bond acceptors. A similar observation applies to benzofuranyl derivatives, where **ADS023** contains three H-bond acceptors while the other benzofuranyl derivatives have four H-bond acceptors. As we show later in the manuscript, the lack of an additional fragment engaged in hydrogen bond formation could be related to the low affinity of **AD021** and **ADS023**. Moreover, both compounds consist of only seven rotatable bonds, while the others have nine or even ten (**ADS029** and **ADS030**), which in this case may make it difficult to fit into the binding site. Anyway, all compounds do not exceed ten rotatable bonds, which is consistent with the drug-likeness Veber assumptions.^50^

SAR showed that substitution at position 4 of the aromatic ring enhances H_3_R antagonistic activity. Following this, derivatives with biphenyl and naphthalene linkers in place of the 1,4-disubstituted aromatic ring were synthesized to increase structural rigidity and lipophilicity (Schemes 3 and 4). **ADS029** and **ADS030** containing the biphenyl linker showed lower affinity to *gp*H_3_R than the parent compounds **ADS022** and **ADS024**. Compounds with a naphthalene linker, viz., **ADS029** and **ADS030**, showed slightly higher activity than those with a biphenyl moiety. Among all derivatives, the most potent compound in the benzene series was **ADS024**, which contained a benzofuranyl substituent at position 1 of the piperidine ring, while the most potent compound in the naphthalene series was **ADS031**, which contained a benzyl moiety at position 1 of the piperidine ring.

Derivatives with the highest potency at *gp*H_3_R (**ADS022**, **ADS024**, **ADS031**, and **ADS032**) were subjected to the *h*H_3_R radioligand displacement assay, which demonstrated a nanomolar affinity among all tested compounds (Table 1). The lowest *K*_i_ value (12.5 nM; p*K*_i_ = 7.90) was observed for **ADS031**. The benzofuranyl derivative **ADS032** showed *K*_i_ = 44.1 nM (p*K*_i_ = 7.36). H_3_R ligands are known to have a distinct affinity profile and species-dependent pharmacology.^51^ The affinity toward the guinea pig and human H_3_R differed, which can be explained by the species-dependent isoforms of the receptor.^52,53^ Replacement of the 1,4-disubstituted aromatic rings of **ADS024** and **ADS025** by biphenyl rings leads to a decrease of affinity at *gp*H_3_R and violations of some log *P* values (*w* log *P*, *m* log *P*, *x* log *P*) according to drug-like criteria for a drug candidate described by Muegge, Ghose, or Lipinski.^54−56^ Results with higher affinities were observed for naphthalene derivatives, including *K*_i_ values evaluated at *h*H_3_R, but only lead compound **ADS031** did not exceed a log *P* of 6 in all predictive methods. All data determined by the SwissADME web tool can be found in the Supporting Information.

#### 2.3.2 Inhibition of *electric eel* AChE and *equine serum* BuChE

All compounds were tested for their ability to inhibit acetylcholinesterase (*ee*AChE from *electric eel*) and butyrylcholinesterase (*eq*BuChE from *equine serum*) using spectroscopic Ellman’s protocol,^57^ modified for 96-well microplates. Target compounds were tested at a screening concentration of 10 μM. Four compounds (**ADS022**, **ADS025**, **ADS029**, and **ADS031**) showed over 60% *ee*AChE inhibition, and hence the IC_50_ value was determined. The highest inhibitory activity was demonstrated by compound **ADS031** (IC_50_ = 1.537 μM). Moreover, a comparison of the structures of **ADS022**, **ADS025**, **ADS029**, and **ADS031** found their AChE inhibitory activity to increase according to the aromatic moiety, i.e., from benzene through biphenyl to naphthalene derivatives. Eight compounds showed over 60% *eq*BuChE inhibition and were qualified for the determination of the IC_50_ value (Table 1). Of these, the IC_50_ values ranged from 0.559 to 2.655 μM, with the lowest IC_50_ value (IC_50_ = 0.559 μM) being observed for **ADS021**, one of the least active compounds at *gp*H_3_R. **ADS031** showed micromolar inhibitory activity for both AChE and BuChE enzymes and demonstrated higher inhibitory activity against BuChE (IC_50_ = 1.353 μM) than AChE (IC_50_ = 1.537 μM). Selectivity is claimed as a therapeutic advantage, but therapy based on a multitarget-directed ligand combining H_3_R antagonism with additional AChE/BuChE inhibitory properties might improve cognitive functions in multifactorial Alzheimer’s disease. Nanomolar affinity at H_3_R of **ADS031** and micromolar inhibition of both cholinesterases could be more helpful, as BuChE takes over the task if AChE is inhibited.

#### 2.3.3 H_3_R Intrinsic Activity

For the lead compound **ADS031**, intrinsic activity at H_3_R was tested in HEK293 cells expressing recombinant human receptors using a cAMP accumulation assay. The tested compound was able to counteract (*R*)-(−)-α-methylhistamine-driven inhibition of cAMP production in forskolin-stimulated cells, which was indicative of its H_3_R antagonist/inverse agonist properties. The IC_50_ value obtained in the H_3_R functional assay was 41.7 ± 8.5 [nM] (*K*_b_ = 0.75 ± 0.15 [nM]).

### 2.4 Molecular Docking Studies

**Figure 3:**
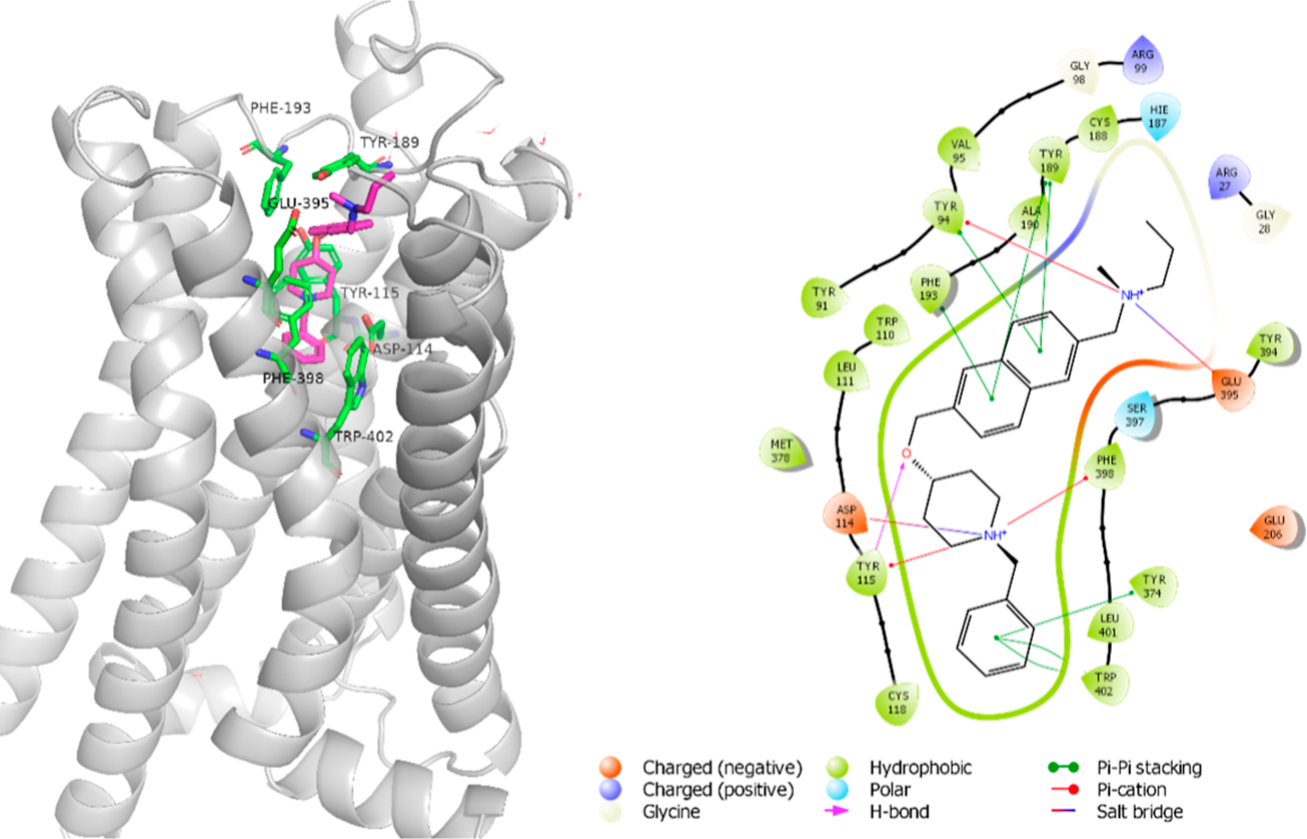
Predicted binding mode for the compound **ADS031** with the highest affinity to the histamine H_3_ receptor (general view—left panel, 2D interaction diagram—right panel).

The obtained compounds were docked to the recently published crystal structure of H_3_R to determine their binding modes.^58^ The induced fit docking procedure revealed similar arrangements in the receptor binding pocket for all derivatives, although they sometimes differed from one another in observed interactions. They were stretched from an orthosteric binding site where the crucial interaction was found with Asp114 from transmembrane domain TM3 toward the extracellular loop ECL2 (Figure 3). Compound **ADS031**, which showed the highest affinity to H_3_R (*K*_i_ = 12.5 nM) as well as other compounds with high pA_2_ values, demonstrated significant interactions within the receptor. The aromatic benzyl or benzofuranyl moiety provided π–π stacking interactions with Trp402. The protonated nitrogen atom from the piperidine ring created a salt bridge with Asp114 and cation–π interactions with Tyr115 and Phe398. The ether oxygen atom could form a hydrogen bond with the hydroxyl group of Tyr115. The second aromatic fragment (benzene, naphthalene, and biphenyl) was engaged in π–π stacking with Phe193 and Tyr189. The terminal protonated amino group formed a salt bridge with Glu395. Contrary to this, compounds with shorter ether linkers, a single-second aromatic ring, and an aromatic nonprotonated amine group, such as **ADS021** and **ADS023**, were characterized by reduced π–π stacking interactions and a lack of a salt bridge, which resulted in low pA_2_ values (below 4).

## 3 Conclusions

Among the tested compounds, **ADS031** (pA_2_ = 7.54) and **ADS024** (pA_2_ = 7.57) demonstrated the highest ex vivo potency as antagonist/inverse agonist at the *gp*H_3_R. Comparing the obtained pA_2_ values presented in Table 1, it was found that the ex vivo potency increases when the *N*-methyl-*N*-propyl-aminomethyl substituent is present in the para position of the aromatic moiety. In the radioligand displacement binding assay studies at the *h*H_3_R, compound **ADS031** showed the highest affinity (*K*_i_ = 12.5 nM), while the benzofuranyl derivative **ADS032** showed a lower nanomolar affinity (*K*_i_ = 44.1 nM). The lead compound **ADS031** was classified as a potent H_3_R antagonist/inverse agonist in the cAMP accumulation assay.

Molecular docking studies in the *h*H_3_R binding pocket indicate that the lead compound **ADS031** creates significant interactions, including π–π stacking interactions between Trp402 for the benzyl moiety, a salt bridge with Asp114, and cation–π interactions with Tyr115 and Phe398 and the protonated nitrogen from the piperidine ring: it also forms a hydrogen bond between the Tyr115 and ether oxygen atoms, and π–π stacking between Phe193 and Tyr189 for the naphthalene fragment.

The inhibitory activity of the novel compounds toward their second target was assessed by an AChE/BuChE spectrophotometric assay. All compounds were tested at a concentration of 10 μM and four of them: **ADS022**, **ADS025**, **ADS029**, and **ADS031** demonstrated over 60% inhibition of *ee*AChE and were hence tested for their IC_50_ value. Compound **ADS031** showed the highest inhibitory activity (IC_50_ = 1.537 μM).

In contrast, eight compounds were qualified for IC_50_ testing against *eq*BuChE. The lowest IC_50_ value was noticed for **ADS021** (IC_50_ = 0.559 μM), one of the least active compounds against *gp*H_3_R, while a lower value was demonstrated by **ADS031**. The most potent compound in the study toward hH_3_R (IC_50_ = 1.353 μM).

Therapy incorporating H_3_R antagonists with additional AChE/BuChE inhibitory effects may improve cognitive functions in Alzheimer’s disease. Combining two or more pharmacophore motifs in a single molecule is one of the strategies for designing multiple targeting ligands. One of the most popular motifs of AChE inhibitors is tacrine. Analogues of tacrine easily lead to potent AChE inhibitors; however, tarcrine has poor therapeutic efficacy and a high prevalence of detrimental effects. Although our lead compounds do not appear to be more active than the known dual-targeting ligands shown in Figure 1, they represent a good basis for structure optimization and promising pharmacological tools for further biological evaluation. Therefore, the assessment of their pharmacokinetic properties will be the subject of our further studies.

## 4 Materials and Methods

### 4.1 Chemistry

The 4-(methylamino)phenol hemisulfate salt, di-*tert*-butyl dicarbonate, 1-benzyl-4-hydroxypiperidine, triphenylphosphine, trifluoroacetic acid, lithium aluminum hydride, 15-crown-5, methyl formate, 4,4′-bis(chloromethyl)-1,1′-biphenyl, *N*-methyl-*N*-propylamine, phosphorus tribromide, NaBH(OAc)_3_, benzofuran-2-carboxylic acid, thionyl chloride (Sigma-Aldrich, St. Louis, MO, USA), diethyl azodicarboxylate, sodium hydride (60% dispersion in mineral oil) (TCI, Tokyo, Japan), benzaldehyde, piperidin-4-ol, propionic anhydride, 4-(bromomethyl)benzonitrile, 3-(bromomethyl)benzonitrile, 2-(bromomethyl)benzonitrile, naphthalene-2,6-dicarboxylic acid (Alfa Aesar, Ward Hill, Massachusetts, USA), and 4 M HCl in dioxane (Fluorochem, Hadfield, UK) were purchased from commercial suppliers and used without further purification.

Nuclear magnetic resonance spectra, ^1^H NMR and ^13^C NMR, were recorded on a Bruker Avance III 600 MHz spectrometer (Bruker, Billerica, MA, USA) in CDCl_3_ and CD_3_OD. The ^1^H NMR spectra were run at 600 MHz, and ^13^C NMR spectra were run at at 150.95 MHz. The chemical shifts are reported in ppm on scale downfield from tetramethylsilane (TMS) as the internal standard, and the signal patterns are indicated as follows: s = singlet, d = doublet, t = triplet, m = multiplet, and br = broad; the number of protons, and *J* approximate coupling constant in Hertz. Elemental analyses (C, H, and N) for all compounds were measured in a PerkinElmer Series II CHNS/O Analyzer 2400 (PerkinElmer, Waltham, MA, USA); the results agreed with the theoretical values within ±0.4%. TLC data were obtained with Merck silica gel 60F254 aluminum sheets. Flash column chromatography using silica gel, 60 Å, 50 μm (J. T. Baker B. V.) used the same solvent system as for TLC. All obtained final free bases were treated with a methanolic solution of oxalic acid or fumaric acid, and the salts were precipitated with dry diethyl ether and crystallized twice from ethanol. All oxalic (**ADS021**, **ADS022**, **ADS023**, **ADS024**, **ADS025**, **ADS026**, **ADS027**, **ADS028**, **ADS029**, **ADS031**, and **ADS032**) and fumaric (**ADS030**) acid salts were obtained as white crystalline solids.

#### 4.1.1 General Procedure for the Preparation of Final Compounds

##### 4.1.1.1 General Procedure for the Preparation of Compounds **ADS021**, **ADS022**, **ADS023**, **ADS024**, **ADS025**, **ADS026**, **ADS027**, **ADS028**, **and ADS030**

LiAlH_4_ (2 equiv) was slowly added to a solution of the corresponding amide (1 equiv) (**5.1**, **5.2**, **12a.1**, **12b.1**, **12c.1**, **12a.2**, **12b.2**, **12c.2**, and **16**) in 50 mL of anhydrous diethyl ether. The reaction mixture was stirred at 36 °C for 3 h. After completing the reaction, the mixture was quenched by the dropwise addition of water (8 equiv) and 10% NaOH solution (8 equiv), stirred for 2 h, and then filtered by Celite. The precipitate was discarded. The solvent was removed under vacuum, and the crude product was purified by column chromatography to yield the pure product.

4-((1-Benzylpiperidin-4-yl)oxy)-*N*-methyl-*N*-propylaniline (**ADS021**): (50%): *R*_f_ = 0.62 (hexane/EtOAc 1:5); ^1^H NMR (CDCl_3_, 600 MHz): δ = 0.90 (t, 3H, *J* = 7.4, NCH_2_CH_2_CH_3_), 1.53–1.59 (m, 2H, NCH_2_CH_2_CH_3_), 1.75–1.81 (m, 2H, H^pip^), 1.92–1.98 (m, 2H, H^pip^), 2.22–2.26 (m, 2H, H^pip^), 2.72–2.76 (m, 2H, H^pip^), 2.85 (s, 3H, NCH_3_), 3.17 (t, 2H, *J* = 7.5, NCH_2_CH_2_CH_3_), 3.51 (s, 2H, CH_2_–C_6_H_5_), 4.08–4.13 (m, 1H, H^pip^), 6.64 (d, 2H, *J* = 9.1, H^Ar^), 6.82 (d, 2H, *J* = 9.1, H^Ar^), 7.22–7.25 (m, 1H, H^Ar^), 7.29–7.33 (m, 4H, H^Ar^); ^13^C NMR (CDCl_3_, 150 MHz): δ = 11.78 (NCH_2_CH_2_CH_3_), 20.16 (NCH_2_CH_2_CH_3_), 31.44 (C^pip^), 39.07 (NCH_3_), 51.02 (NCH_2_CH_2_CH_3_), 55.89 (C^pip^), 63.28 (CH_2_C_6_H_5_), 74.64 (C^pip^), 114.27, 118.40, 127.16, 128.39, 129.31, 138.85, 145.22, 149.10 (C^Ar^).

Anal. Calcd for oxalic salt (C_22_H_30_N_2_O·C_2_H_2_O_4_·H_2_O): C, 65.88; H, 7.60; N, 6.40. Found: C, 66.51; H, 7.81; N, 6.41; mp 156–157.5 °C.

4-((1-(Benzofuran-2-ylmethyl)piperidin-4-yl)oxy)-*N*-methyl-*N*-propylaniline (**ADS023**): (80%): *R*_f_ = 0.60 (hexane/EtOAc 1:5); ^1^H NMR (CDCl_3_, 600 MHz): δ = 0.90 (t, 3H, *J* = 7.4, NCH_2_CH_2_CH_3_), 1.52–1.59 (m, 2H, NCH_2_CH_2_CH_3_), 1.80–1.87 (m, 2H, CH_2_^pip^), 1.95–2.00 (m, 2H, CH_2_^pip^), 2.36–2.41 (m, 2H, CH_2_^pip^), 2.79–2.83 (m, 2H, CH_2_^pip^), 2.84 (s, 3H, CH_3_), 3.16 (t, 2H, *J* = 7.4, NCH_2_CH_2_CH_3_), 3.71 (s, 2H, CH_2_), 4.10–4.15 (m, 1H, CH^pip^), 6.58 (s, 1H, CH^furan^), 6.64 (d, 2H, *J* = 9.1, H^Ar^), 6.81 (d, 2H, *J* = 9.0, H^Ar^), 7.17–7.24 (m, 2H, H^Ar^), 7.47 (d, 1H, *J* = 8.2, H^Ar^), 7.51 (d, 1H, *J* = 7.6, H^Ar^); ^13^C NMR (150 MHz, CDCl_3_): δ = 11.78 (NCH_2_CH_2_CH_3_), 20.14 (NCH_2_CH_2_CH_3_), 31.18 (C^pip^), 39.06 (CH_3_), 50.84 (C^pip^), 55.75 (NCH_2_CH_2_CH_3_), 55.86 (CH_2_), 74.13 (C^pip^), 105.69 (C^furan^), 111.53, 114.23, 118.36, 120.88, 122.83, 124.07, 128.56, 145.22, 148.95, 155.14, 155.31 (C^Ar^).

Anal. Calcd for oxalic salt (C_24_H_30_N_2_O_2_·2C_2_H_2_O_4_·1.5H_2_O): C, 57.43; H, 6.37; N, 4.58. Found: C, 57.51; H, 5.86; N, 5.00; mp 121–123 °C.

*N*-(4-(((1-Benzylpiperidin-4-yl)oxy)methyl)benzyl)-*N*-methylpropan-1-amine (**ADS022**): (82%): *R*_f_ = 0.43 (CH_2_Cl_2_/MeOH/NH_3(aq)_ 8:1:1%); ^1^H NMR (CDCl_3_, 600 MHz): δ = 0.89 (t, 3H, *J* = 7.4, NCH_2_CH_2_CH_3_), 1.49–1.55 (m, 2H, NCH_2_CH_2_CH_3_), 1.65–1.71 (m, 2H, CH_2_^pip^), 1.88–1.93 (m, 2H, CH_2_^pip^), 2.10–2.16 (m, 2H, CH_2_^pip^), 2.17 (s, 3H, CH_3_), 2.30–2.33 (m, 2H, NCH_2_CH_2_CH_3_), 2.73–2.77 (m, 2H, CH_2_^pip^), 3.39–3.43 (m, 1H, CH^pip^), 3.45 (s, 1H, CH_2_N), 3.48 (s, 2H, CH_2_C_6_H_5_), 4.52 (s, 2H, OCH_2_C_6_H_5_), 7.22–7.32 (m, 9H, H^Ar^); ^13^C NMR (150 MHz, CDCl_3_): δ = 12.06 (NCH_2_CH_2_CH_3_), 20.77 (NCH_2_CH_2_CH_3_), 31.59 (C^pip^), 42.44(C^pip^), 51.39 (C^pip^), 59.72 (NCH_2_CH_2_CH_3_), 62.30 (CH_2_C_6_H_5_), 63.24 (CH_2_N), 69.77 (O_CH_2_C_6_H_4_), 74.80 (C^pip^), 127.13, 127.61, 128.37, 129.24, 129.29, 137.80, 138.78 (C^Ar^).

Anal. Calcd for oxalic salt (C_24_H_34_N_2_O·3C_2_H_2_O_4_·0.5H_2_O): C, 55.81; H, 6.40; N, 4.34. Found: C, 57.61; H, 6.18; N, 4.29; mp 158–160 °C.

*N*-(4-(((1-(Benzofuran-2-ylmethyl)piperidin-4-yl)oxy)methyl)benzyl)-*N*-methylpropan-1-amine (**ADS024**): (80%): *R*_f_ = 0.74 (CH_2_Cl_2_/MeOH/NH_3(aq)_ 8:1:1%); ^1^H NMR (CDCl_3_, 600 MHz): δ = 0.89 (t, 3H, *J* = 7.3, NCH_2_CH_2_CH_3_), 1.49–1.56 (m, 2H, NCH_2_CH_2_CH_3_), 1.70–1.77 (m, 2H, CH_2_^pip^), 1.90–1.95 (m, 2H, CH_2_^pip^), 2.18 (s, 3H, CH_3_), 2.26–2.34 (m, 4H, CH_2_^pip^, NCH_2_CH_2_CH_3_), 2.80–2.85 (m, 2H, CH_2_^pip^), 3.40–3.45 (m, 1H, CH^pip^), 3.47 (s, 2H, C_6_H_4_CH_2_N), 3.67 (s, 2H, CH_2_NC_5_H_9_), 4.50 (s, 2H, OCH_2_C_6_H_4_), 6.56 (s, 1H, CH^furan^), 7.17–7.20 (m, 2H, CH^Ar^), 7.22–7.25 (m, 2H, CH^Ar^), 7.45–7.52 (m, 2H, CH^Ar^); ^13^C NMR (150 MHz, CDCl_3_): δ = 12.03 (NCH_2_CH_2_CH_3_), 20.62 (NCH_2_CH_2_CH_3_), 31.32 (2× CH_2_^pip^), 42.31 (CH_3_), 51.17 (CH_2_^pip^), 55.68 (CH_2_NC_5_H_9_), 59.56 (NCH_2_CH_2_CH_3_), 62.13 (C_6_H_4_CH_2_N), 69.75 (OCH_2_C_6_H_4_), 74.18 (CH^pip^), 105.67 (C^furan^), 111.50, 120.85, 122.81, 124.04, 127.61, 128.54, 129.32, 137.81, 138.37, 155.12 (C^Ar^).

Anal. Calcd for oxalic salt (C_26_H_34_N_2_O_2_·2C_2_H_2_O_4_·0.5H_2_O): C, 60.49; H, 6.60; N, 4.70. Found: C, 60.63; H, 6.44; N, 4.63; mp 179.4–181.1 °C.

*N*-(3-(((1-Benzylpiperidin-4-yl)oxy)methyl)benzyl)-*N*-methylpropan-1-amine (**ADS025**): (45%): *R*_f_ = 0.71 (CH_2_Cl_2_/MeOH/NH_3(aq)_ 8:1:1%); ^1^H NMR (CDCl_3_, 600 MHz): δ = 0.89 (t, 3H, *J* = 7.38, CH_3_CH_2_CH_2_N), 1.49–1.56 (m, 2H, CH_3_CH_2_CH_2_N), 1.64–1.71 (m, 2H, CH_2_^pip^), 1.87–1.93 (m, 2H, CH_2_^pip^), 2.09–2.15 (m, 2H, CH_2_^pip^), 2.17 (s, 3H, CH_3_), 2.31 (t, 3H, *J* = 7.44, CH_3_CH_2_CH_2_N), 2.72–2.77 (m, 2H, CH_2_^pip^), 3.38–3.43 (m, 1H, CH^pip^), 3.46 (s, 2H, C_6_H_5_CH_2_), 3.48 (s, 2H, CH_2_N), 4.52 (s, 2H, OCH_2_C_6_H_4_), 7.20–7.32 (m, 9H, CH^Ar^); ^13^C NMR (150 MHz, CDCl_3_): δ = 12.08 (CH_3_CH_2_CH_2_N), 20.73 (CH_3_CH_2_CH_2_N), 31.55 (2× C^pip^), 42.49 (CH_3_), 51.38 (2× C^pip^), 59.71 (CH_3_CH_2_CH_2_N), 62.43 (C_6_H_5_CH_2_), 63.20 (CH_2_N), 69.90 (OCH_2_C_6_H_4_), 74.80 (C^pip^), 126.27, 127.10, 128.34, 128.39, 129.26, 138.79 (C^Ar^).

Anal. Calcd for oxalic salt (C_24_H_34_N_2_O·3C_2_H_2_O_4_): C, 56.60; H, 6.33; N, 4.40. Found: C, 56.61; H, 6.42; N, 4.44; mp 176.6–178.2 °C.

*N*-(3-(((1-(Benzofuran-2-ylmethyl)piperidin-4-yl)oxy)methyl)benzyl)-*N*-methylpropan-1-amine (**ADS026**): (65%): *R*_f_ = 0.59 (CH_2_Cl_2_/MeOH/NH_3(aq)_ 8:1:1%); ^1^H NMR (CDCl_3_, 600 MHz): δ = 0.89 (t, 3H, *J* = 7.4, CH_3_CH_2_CH_2_N), 1.49–1.56 (m, 2H, CH_3_CH_2_CH_2_N), 1.71–1.78 (m, 2H, CH_2_^pip^), 1.90–1.96 (m, 2H, CH_2_^pip^), 2.18 (s, 3H, CH_3_), 2.24–2.34 (m, 4H, CH_2_^pip^, CH_3_CH_2_CH_2_N), 2.79–2.86 (m, 2H, CH_2_^pip^), 3.39–3.44 (m, 1H, CH^pip^), 3.46 (s, 2H, C_8_H_5_CH_2_), 3.68 (s, 2H, CH_2_N), 4.52 (s, 2H, OCH_2_C_6_H_4_), 6.57 (s, 1H, CH^furan^), 7.17–7.29 (m, 6H, CH^Ar^), 7.45–7.54 (m, 2H, CH^Ar^); ^13^C NMR (CDCl_3_, 150 MHz): δ = 12.08 (CH_3_CH_2_CH_2_N), 20.69 (CH_3_CH_2_CH_2_N), 31.32 (2× C^pip^), 42.46 (CH_3_), 51.21 (2× C^pip^), 55.66 (CH_3_CH_2_CH_2_N), 59.69 (C_8_H_5_CH_2_), 62.40 (CH_2_N), 69.93 (OCH_2_C_6_H_4_), 74.27 (C^pip^), 105.68 (C^furan^), 111.50, 120.84, 122.80, 124.04, 126.33, 128.39, 139.00, 139.46, 155.25 (C^Ar^).

Anal. Calcd for oxalic salt (C_26_H_34_N_2_O_2_·2C_2_H_2_O_4_·1H_2_O): C, 59.59; H, 6.67; N, 4.63. Found: C, 59.69; H, 6.52; N, 4.29; mp 187–189 °C.

*N*-(2-(((1-Benzylpiperidin-4-yl)oxy)methyl)benzyl)-*N*-methylpropan-1-amine (**ADS027**): (77%): *R*_f_ = 0.34 (CH_2_Cl_2_/MeOH/NH_3(aq)_ 8:1:1%); ^1^H NMR (CDCl_3_, 600 MHz): δ = 0.87 (t, 3H, *J* = 7.32, NCH_2_CH_2_CH_3_), 1.46–1.53 (m, 2H, NCH_2_CH_2_CH_3_), 1.66–1.71 (m, 2H, CH_2_^pip^), 1.89–1.95 (m, 2H, CH_2_^pip^), 2.11–2.17 (m, 5H, CH_2_^pip^, CH_3_), 2.30 (t, 2H, J = 7.26, NCH_2_CH_2_CH_3_), 2.73–2.79 (m, 2H, CH_2_^pip^), 3.39–3.44 (m, 1H, CH^pip^), 3.47 (s, 2H, CH_2_C_6_H_5_), 3.49 (s, 1H, CH_2_N), 4.65 (s, 2H, OCH_2_C_6_H_4_), 7.20–7.41 (m, 9H, H^Ar^); ^13^C NMR (CDCl_3_, 150 MHz): δ = 12.10 (CH_3_CH_2_CH_2_N), 20.27 (CH_3_CH_2_CH_2_N), 31.59 (C^pip^ 2×), 42.22 (CH_3_), 51.42 (C^pip^ 2×), 60.06 and 60.19 (CH_2_C_6_H_5_, CH_3_CH_2_CH_2_N), 63.25 (CH_2_N), 67.49 (OCH_2_C_6_H_5_), 75.10 (C^pip^), 127.12, 128.35, 128.55, 129.30, 130.11, 137.59, 137.96, 138.75 (C^Ar^).

Anal. Calcd for oxalic salt (C_24_H_34_N_2_O·2.5C_2_H_2_O_4_·H_2_O): C, 57.13; H, 6.78; N, 4.60. Found: C, 57.15; H, 6.45; N, 4.38; mp 176–178 °C.

*N*-(2-(((1-(Benzofuran-2-ylmethyl)piperidin-4-yl)oxy)methyl)benzyl)-*N*-methylpropan-1-amine (**ADS028**): (43%): *R*_f_ = 0.52 (CH_2_Cl_2_/MeOH/NH_3(aq)_ 8:1:1%); ^1^H NMR (CDCl_3_, 600 MHz): δ = 0.84–0.90 (m, 3H, NCH_2_CH_2_CH_3_), 1.42–1.53 (m, 2H, NCH_2_CH_2_CH_3_), 1.71–1.79 (m, 2H, CH_2_^pip^), 1.92–1.97 (m, 2H, CH_2_^pip^), 2.10 (s, 3H, CH_3_), 2.27–2.32 (m 2H, NCH_2_CH_2_CH_3_), 2.79–2.85 (m, 2H, CH_2_^pip^), 3.39–3.44 (m, 1H, CH^pip^), 3.47 (s, 2H, CH_2_C_8_H_5_O), 3.66 (s, 2H, OCH_2_C_6_H_4_), 4.63 (s, 2H, CH_2_N), 6.55 (s, 1H, H^furan^), 7.15–7.42 (m, 6H, H^Ar^), 7.45–7.51 (m, 2H, H^Ar^); ^13^C NMR (CDCl_3_, 150 MHz): δ = 12.07 (CH_3_CH_2_CH_2_N), 20.70 (CH_3_CH_2_CH_2_N), 31.32 (C^pip^ 2×), 42.16 (CH_3_), 51.22 (C^pip^ 2×), 55.64 (CH_2_C_8_H_5_O), 60.06 (CH_3_CH_2_CH_2_N), 67.46 (CH_2_N), 70.33 (OCH_2_C_6_H_4_), 74.26 (C^pip^), 105.64 (C^furan^), 111.45, 120.79, 122.75, 127.06, 127.32, 128.51, 130.15, 137.57, 155.00.

Anal. Calcd for oxalic salt (C_26_H_34_N_2_O_2_·2C_2_H_2_O_4_·H_2_O): C, 59.69; H, 6.52; N, 4.29. Found: C, 59.59; H, 6.67; N, 4.63; mp 156–158 °C.

*N*-((4′-(((1-(Benzofuran-2-ylmethyl)piperidin-4-yl)oxy)methyl)-[1,1′-biphenyl]-4-yl)methyl)-*N*-methylpropan-1-amine (**ADS030**): (99%): *R*_f_ = 0.56 (CH_2_Cl_2_/MeOH/NH_3(aq)_ 8:1:1%); ^1^H NMR (600 MHz, CDCl_3_): δ = 0.91 (t, 3H, *J* = 7.8, CH_3_CH_2_CH_2_N), 1.53–1.58 (m, 2H, CH_3_CH_2_CH_2_N), 1.73–1.78 (m, 2H, CH_2_^pip^), 1.92–1.97 (m, 2H, CH_2_^pip^), 2.21 (s, 3H, CH_3_), 2.25–2.33 (m, 2H, CH_2_^pip^), 2.35 (t, 2H, *J* = 7.2, CH_3_CH_2_CH_2_N), 2.79–2.86 (m, 2H, CH_2_^pip^), 3.42–3.48 (m, 1H, CH^pip^), 3.51 (s, 2H, CH_2_C_12_H_8_), 3.67 (s, 2H, CH_2_C_8_H_5_), 4.55 (s, 2H, OCH_2_), 6,57 (s, 1H, H^furan^), 7.17–7.58 (m, 12H, H^Ar^); ^13^C NMR (150 MHz, CDCl_3_): δ = 12.06 (CH_3_CH_2_CH_2_N), 20.71 (CH_3_CH_2_CH_2_N), 31.28 (CH_2_^pip^), 42.41 (CH_3_), 51.12 (CH_2_^pip^), 55.63 (CH_2_C_8_H_5_), 59.69 (CH_3_CH_2_CH_2_N), 62.11 (CH_2_N(CH_3_)C_3_H_7_), 69.57 (OCH_2_), 74.13(CH^pip^), 105.65 (CH^furan^), 111.47, 120.82, 122.77, 124.01, 127.00, 127.03, 128.08, 128.47, 137.97, 139.67, 140.39, 155.22 (C^Ar^).

Anal. Calcd for fumarate salt (C_32_H_38_N_2_O_2_·2C_4_H_4_O_4_): C, 67.21; H, 6.49; N, 3.92. Found: C, 67.12; H, 6.19; N, 3.93; mp 167–169 °C.

##### 4.1.1.2 General Procedure for the Preparation of Compound **ADS029**

To a solution of corresponding chloride (**14**) (1 equiv) and K_2_CO_3_ (2 equiv) in 20.0 mL of acetonitrile, *N*-methyl-*N*-propylamine was added (2 equiv). The mixture was stirred at room temperature for 12 h. Water (20 mL) was then added, and the mixture was extracted with dichloromethane (3× 20 mL). The organic layer was dried over Na_2_SO_4_, the solvent was removed under vacuum, and the crude product was purified by column chromatography to yield the pure product to give compounds **ADS029** as a sticky oil.

*N*-((4′-(((1-Benzylpiperidin-4-yl)oxy)methyl)-[1,1′-biphenyl]-4-yl)methyl)-*N*-methylpropan-1-amine (**ADS029**): (51%): *R*_f_ = 0.56 (CHCl_3_/MeOH 9:1); ^1^H NMR (CDCl_3_, 600 MHz): δ = 0.91 (t, 3H, *J* = 7.32, CH_3_CH_2_CH_2_N), 1.52–1.59 (m, 2H, CH_3_CH_2_CH_2_N), 1.67–1.74 (m, 2H, CH_2_^pip^), 1.91–1.97 (m, 2H, CH_2_^pip^), 2.13–2.21 (m, 2H, CH_2_^pip^), 2.22 (s, 3H, CH_3_), 2.36 (t, 2H, *J* = 7.44, CH_3_CH_2_CH_2_N), 2.72–2.80 (m, 2H, CH_2_^pip^), 3.43–3.47 (m, 1H, CH^pip^), 3.51 (s, 2H, CH_2_C_6_H_5_), 3.52 (s, 2H, CH_2_C_12_H_8_), 4.56 (s, 2H, OCH_2_), 7.20–7.66 (m, 13H, H^Ar^); ^13^C NMR (150 MHz, CDCl_3_): δ = 12.10 (CH_3_CH_2_CH_2_N), 20.78 (CH_3_CH_2_CH_2_N), 31.55 (CH_2_^pip^), 42.48 (CH_3_), 51.25 (CH_2_^pip^), 59.76 (CH_3_CH_2_CH_2_N), 62.17 (CH_2_C_6_H_5_), 63.23 (CH_2_N(CH_3_)C_3_H_7_), 69.61 (OCH_2_), 74.86 (CH^pip^), 127.05, 127.14, 127.23, 128.14, 128.38, 129.32, 129.64, 138.09, 138.60, 138.76, 139.72, 140.43 (C^Ar^).

Anal. Calcd for oxalic salt (C_30_H_38_N_2_O·3C_2_H_2_O_4_): C, 60.67; H, 6.22; N, 3.93. Found: C, 60.71; H, 6.51; N, 4.18; mp 164–165 °C.

##### 4.1.1.3 General Procedure for the Preparation of Compounds **ADS031** and **ADS032**

To the suspension of *N*-methyl-*N*-((6-((piperidin-4-yloxy)methyl)naphthalen-2-yl)methyl)propan-1-amine hydrochloride **23** (1 equiv) and benzofuran-2-carbaldehyde (1.3 equiv) or benzaldehyde (1.3 equiv) in 1,2-dichloroethane (2 mL), NaBH(OAc)_3_ (5 equiv) was added. The reaction mixture was stirred under an argon atmosphere overnight. After completion of the reaction, DCM (5 mL) and a 5% aq solution of NaHCO_3_ (10 mL) were added, and the biphasic mixture was stirred for 30 min. The layers were separated, and the aqueous layer was additionally extracted with DCM. The combined organic extracts were then dried over MgSO_4_, and the solvent was removed in vacuo. The crude products were purified by silica gel-flash column chromatography (eluent CH_2_Cl_2_/MeOH, gradient 40:1 to 10:1) to give sticky oils.

*N*-((6-(((1-Benzylpiperidin-4-yl)oxy)methyl)naphthalen-2-yl)methyl)-*N*-methylpropan-1-amine (**ADS031**): (61%): *R*_f_ = 0.20 (CH_2_Cl_2_/MeOH 8:1); ^1^H NMR (600 MHz, CDCl_3_): δ = 0.91 (t, 3H, *J* = 7.8 Hz, CH_3_CH_2_CH_2_N), 1.52–1.59 (m, 2H, CH_3_CH_2_CH_2_N), 1.67–1.75 (m, 2H, CH_2_^pip^), 1.90–1.96 (m, 2H, CH_2_^pip^), 2.08–2.16 (m, 2H, CH_2_^pip^), 2.21 (s, 3H, CH_3_), 2.36 (t, 2H, *J* = 7.8 Hz, CH_3_CH_2_CH_2_N), 2.73–2.79 (m, 2H, CH_2_^pip^), 3.42–3.47 (m, 1H, CH^pip^), 3.48 (s, 2H, C_6_H_5_CH_2_), 3.61 (s, 2H, NCH_2_), 4.68 (s, 2H, OCH_2_), 7.21–7.26 (m, 1H, H^Ar^), 7.28–7.32 (m, 4H, H^Ar^), 7.43–7.49 (m, 2H, H^Ar^), 7.69–7.71 (m, 1H, H^Ar^), 7.73–7.79 (m, 3H, H^Ar^); ^13^C NMR (CDCl_3_, 150 MHz): δ = 12.10 (CH_3_CH_2_CH_2_N), 20.77 (CH_3_CH_2_CH_2_N), 31.58 (CH_2_^pip^), 42.57 (CH_3_), 51.35 (CH_2_^pip^), 59.83 (CH_3_CH_2_CH_2_N), 62.68 (C_6_H_5_CH_2_), 63.21 (NCH_2_), 69.97 (OCH_2_), 74.68 (CH^pip^), 125.96, 126.09, 127.12, 127.49, 127.82, 127.95, 128.10, 128.36, 129.29, 132.75, 133.03, 136.32, 137.15, 138.77 (C^Ar^).

Anal. Calcd for oxalic salt (C_28_H_36_N_2_O·2C_2_H_2_O_4_·0.5H_2_O): C, 63.46; H, 6.82; N, 4.63. Found: C, 63.51; H, 6.83; N, 4.49; mp 154–156 °C.

*N*-((6-(((1-(Benzofuran-2-ylmethyl)piperidin-4-yl)oxy)methyl)naphthalen-2-yl)methyl)-*N*-methylpropan-1-amine (**ADS032**): (47%): *R*_f_ = 0.33 (CH_2_Cl_2_/MeOH 8:1); ^1^H NMR (600 MHz, CDCl_3_): δ = 0.90 (t, 3H, *J* = 7.8 Hz, CH_3_CH_2_CH_2_N), 1.52–1.60 (m, 2H, CH_3_CH_2_CH_2_N), 1.74–1.81 (m, 2H, CH_2_^pip^), 1.92–1.99 (m, 2H, CH_2_^pip^), 2.21 (s, 3H, CH_3_), 2.24–2.30 (m, 2H, CH_2_^pip^), 2.36 (t, 2H, *J* = 7.8 Hz, CH_3_CH_2_CH_2_N), 2.80–2.87 (m, 2H, CH_2_^pip^), 3.43–3.50 (m, 1H, CH^pip^), 3.61 (s, 2H, C_8_H_5_CH_2_), 3.67 (s, 2H, NCH_2_), 4.67 (s, 2H, OCH_2_), 6.57 (s, 1H, CH^furan^), 7.17–7.26 (m, 2H, H^Ar^), 7.42–7.53 (m, 4H, H^Ar^), 7.69–7.78 (m, 4H, H^Ar^); ^13^C NMR (CDCl_3_, 150 MHz): δ = 12.10 (CH_3_CH_2_CH_2_N), 20.77 (CH_3_CH_2_CH_2_N), 31.34 (CH_2_^pip^), 42.58 (CH_3_), 51.19 (CH_2_^pip^), 55.67 (C_8_H_5_CH_2_), 59.83 (CH_3_CH_2_CH_2_N), 62.68 (NCH_2_), 69.97 (OCH_2_), 74.08 (CH^pip^), 105.68 (CH^furan^), 111.51, 120.84, 122.81, 124.04, 126.11, 127.83, 127.94, 128.10, 128.12, 128.50, 133.04, 136.22, 137.19, 155.06 (C^Ar^).

Anal. Calcd for oxalic salt (C_30_H_36_N_2_O_2_·2.5C_2_H_2_O_4_): C, 61.67; H, 6.06; N, 4.11. Found: C, 61.72; H, 6.14; N, 4.08; mp 145–148 °C.

Details of the synthesis of all semiproducts, including NMR spectra, are presented in Supporting Information.

### 4.2 Biological Activity

The compounds were tested against H_3_R by an ex vivo assay using isolated guinea pig ileum electrically stimulated to contraction, according to Vollinga et al.^47^ The pA_2_-values were calculated according to Arunlakshana and Schild.^59^ The radioligand-displacement assay was performed in membrane fractions of HEK-293 cells stably expressing hH_3_R. Cell cultivation and membrane preparation were performed according to Kottke et al.^48^ The *K*_i_ values were calculated from the IC_50_ values using the Cheng–Prusoff equation.^60^ The statistical calculations were performed on −log(*K*_i_). The mean values and 95% confidence intervals were transformed to nanomolar concentrations. The inhibitory activities toward AChE and BuChE of novel ADS compounds were assessed in a spectrophotometric Ellman’s assay using *electric eel* AChE (*ee*AChE) and *equine* BuChE (*eq*BuChE).^57^ Intracellular cAMP accumulation was measured with a homogeneous TR-FRET immunoassay using the LANCE Ultra cAMP kit (PerkinElmer) and HEK293 cells stably expressing the human histamine H_3_ receptor.^61^ A more detailed description of the biological methods is presented in the Supporting Information.

### 4.3 Molecular Docking Studies

Ligand structures were prepared with LigPrep (Schrodinger Suite) from SMILES strings. Protonation states were predicted with Epik at pH 7.4 ± 0.2. The recently obtained crystal structure of H_3_R was used for the studies (PDB code: 7F61).^58^ The binding modes of the analyzed compounds were predicted by the Induced Fit Docking procedure (Schrodinger Suite) based on the standard protocol and the OPLS_2005 force field. The binding site was defined as a box with the center at Asp3.32 and size adjusted to allow docking of ligands with length ≤25 Å. The obtained ligand poses were assessed by IFDScore and GlideScore. The interaction networks were visualized with Maestro (Schrodinger Suite) and PyMOL 0.99rc6 (DeLano Scientific LLC).

## Supporting Information Available

Chemical synthesis and NMR spectra; description of the biological methods, including an ex vivo assay for histamine H_3_R receptor antagonists on guinea pig ileum, an *h*H_3_R radioligand displacement binding assay, inhibition of *electric eel* AChE and *equine* serum BuChE, and H_3_R intrinsic activity, and physiochemical parameters determined at the SwissADME web tool (PDF)

## References

[ref1] Arrang J. M.; Garbarg M.; Schwartz J. C. Auto-Inhibition of Brain Histamine Release Mediated by a Novel Class (H3) of Histamine Receptor. Nature 1983, 302 (5911), 832–837. 10.1038/302832a0. 6188956

[ref2] Panula P.; Chazot P. L.; Cowart M.; Gutzmer R.; Leurs R.; Liu W. L. S.; Stark H.; Thurmond R. L.; Haas H. L. International Union of Basic and Clinical Pharmacology. XCVIII. Histamine Receptors. Pharmacol. Rev. 2015, 67 (3), 601–655. 10.1124/pr.114.010249. 26084539 PMC4485016

[ref3] Schlicker E.; Malinowska B.; Kathmann M.; Göthert M. Modulation of Neurotransmitter Release via Histamine H3 Heteroreceptors. Fundam. Clin. Pharmacol. 1994, 8 (2), 128–137. 10.1111/j.1472-8206.1994.tb00789.x. 8020871

[ref4] Clapham J.; Kilpatrick G. J. Histamine H3 Receptors Modulate the Release of [3H]-acetylcholine from Slices of Rat Entorhinal Cortex: Evidence for the Possible Existence of H3 Receptor Subtypes. Br. J. Pharmacol. 1992, 107 (4), 919–923. 10.1111/j.1476-5381.1992.tb13386.x. 1334753 PMC1907926

[ref5] Yokotani K.; Murakami Y.; Okada S.; Wang M.; Nakamura K. Histamine H3 Receptor-Mediated Inhibition of Endogenous Acetylcholine Release from the Isolated, Vascularly Perfused Rat Stomach. Eur. J. Pharmacol. 2000, 392 (1–2), 23–29. 10.1016/S0014-2999(00)00085-6. 10748268

[ref6] Schlicker E.; Fink K.; Detzner M.; Göthert M. Histamine Inhibits Dopamine Release in the Mouse Striatum via Presynaptic H3 Receptors. J. Neural. Transm. 1993, 93 (1), 1–10. 10.1007/BF01244933. 8396945

[ref7] Schlicker E.; Fink K.; Hinterthaner M.; Göthert M. Inhibition of Noradrenaline Release in the Rat Brain Cortex via Presynaptic H3 Receptors. Naunyn-Schmiedebergs Arch. Pharmakol. 1989, 340 (6), 633–638. 10.1007/BF00717738. 2615855

[ref8] Blandizzi C.; Tognetti M.; Colucci R.; Tacca M. D. Histamine H3 Receptors Mediate Inhibition of Noradrenaline Release from Intestinal Sympathetic Nerves. Br. J. Pharmacol. 2000, 129 (7), 1387–1396. 10.1038/SJ.BJP.0703194. 10742294 PMC1571974

[ref9] Schlicker E.; Betz R.; Göthert M. Histamine H3 Receptor-Mediated Inhibition of Serotonin Release in the Rat Brain Cortex. Naunyn-Schmiedebergs Arch. Pharmakol. 1988, 337 (5), 588–590. 10.1007/BF00182737. 3412497

[ref10] Witkin J. M.; Nelson D. L. Selective Histamine H3 Receptor Antagonists for Treatment of Cognitive Deficiencies and Other Disorders of the Central Nervous System. Pharmacol. Ther. 2004, 103 (1), 1–20. 10.1016/j.pharmthera.2004.05.001. 15251226

[ref11] Robin Ganellin C.; Schwartz J. C.; Stark H. Discovery of Pitolisant, the First Marketed Histamine H3-Receptor Inverse Agonist/Antagonist for Treating Narcolepsy. Success. Drug Discovery 2018, 3, 359–381. 10.1002/9783527808694.CH13.

[ref12] Takahashi K.; Suwa H.; Ishikawa T.; Kotani H. Targeted Disruption of H3 Receptors Results in Changes in Brain Histamine Tone Leading to an Obese Phenotype. J. Clin. Invest. 2002, 110 (12), 1791–1799. 10.1172/JCI200215784. 12488429 PMC151650

[ref13] Pillot C.; Ortiz J.; Héron A.; Ridray S.; Schwartz J. C.; Arrang J. M. Ciproxifan, a Histamine H3-Receptor Antagonist/Inverse Agonist, Potentiates Neurochemical and Behavioral Effects of Haloperidol in the Rat. J. Neurosci. 2002, 22 (16), 7272–7280. 10.1523/JNEUROSCI.22-16-07272.2002. 12177222 PMC6757889

[ref14] Glase S. A.; Robertson D. W.; Wise L. D. Chapter 2. Attention Deficit Hyperactivity Disorder: Pathophysiology and Design of New Treatments. Annu. Rep. Med. Chem. 2002, 37, 11–20. 10.1016/S0065-7743(02)37003-9.

[ref15] Rapanelli M.; Pittenger C. Histamine and Histamine Receptors in Tourette Syndrome and Other Neuropsychiatric Conditions. Neuropharmacology 2016, 106, 85–90. 10.1016/j.neuropharm.2015.08.019. 26282120

[ref16] Pullen L. C.; Picone M.; Tan L.; Johnston C.; Stark H. Cognitive Improvements in Children with Prader-Willi Syndrome Following Pitolisant Treatment-Patient Reports. J. Pediatr. Pharmacol. Therapeut. 2019, 24 (2), 166–171. 10.5863/1551-6776-24.2.166. PMC6478354 31019411

[ref17] Iida T.; Yoshikawa T.; Kárpáti A.; Matsuzawa T.; Kitano H.; Mogi A.; Harada R.; Naganuma F.; Nakamura T.; Yanai K. JNJ10181457, a Histamine H3 Receptor Inverse Agonist, Regulates in Vivo Microglial Functions and Improves Depression-like Behaviours in Mice. Biochem. Biophys. Res. Commun. 2017, 488 (3), 534–540. 10.1016/j.bbrc.2017.05.081. 28526411

[ref18] Baronio D.; Castro K.; Gonchoroski T.; de Melo G. M.; Nunes G. D. F.; Bambini-Junior V.; Gottfried C.; Riesgo R. Effects of an H3R Antagonist on the Animal Model of Autism Induced by Prenatal Exposure to Valproic Acid. PLoS One 2015, 10 (1), e0116363 10.1371/JOURNAL.PONE.0116363. 25560049 PMC4283962

[ref19] Johannsen P. Long-Term Cholinesterase Inhibitor Treatment of Alzheimer’s Disease. CNS Drugs 2004, 18 (12), 757–768. 10.2165/00023210-200418120-00001. 15377166

[ref20] Dauvilliers Y.; Bassetti C.; Lammers G. J.; Arnulf I.; Mayer G.; Rodenbeck A.; Lehert P.; Ding C. L.; Lecomte J. M.; Schwartz J. C. Pitolisant versus Placebo or Modafinil in Patients with Narcolepsy: A Double-Blind, Randomised Trial. Lancet Neurol. 2013, 12 (11), 1068–1075. 10.1016/S1474-4422(13)70225-4. 24107292

[ref21] Lin J. S.; Sergeeva O. A.; Haas H. L. Histamine H3 Receptors and Sleep-Wake Regulation. J. Pharmacol. Exp. Ther. 2011, 336 (1), 17–23. 10.1124/jpet.110.170134. 20864502

[ref22] Passani M. B.; Lin J. S.; Hancock A.; Crochet S.; Blandina P. The Histamine H3 Receptor as a Novel Therapeutic Target for Cognitive and Sleep Disorders. Trends Pharmacol. Sci. 2004, 25 (12), 618–625. 10.1016/j.tips.2004.10.003. 15530639

[ref23] Mochizuki T.; Okakura-Mochizuki K.; Horii A.; Yamamoto Y.; Yamatodani A. Histaminergic Modulation of Hippocampal Acetylcholine Release in Vivo. J. Neurochem. 1994, 62 (6), 2275–2282. 10.1046/j.1471-4159.1994.62062275.x. 7910631

[ref24] Fox G. B.; Esbenshade T. A.; Pan J. B.; Radek R. J.; Krueger K. M.; Yao B. B.; Browman K. E.; Buckley M. J.; Ballard M. E.; Komater V. A.; Miner H.; Zhang M.; Faghih R.; Rueter L. E.; Bitner R. S.; Drescher K. U.; Wetter J.; Marsh K.; Lemaire M.; Porsolt R. D.; Bennani Y. L.; Sullivan J. P.; Cowart M. D.; Decker M. W.; Hancock A. A. Pharmacological Properties of ABT-239 [4-(2-{2-[(2R)-2-Methylpyrrolidinyl]Ethyl}-Benzofuran-5-Yl)Benzonitrile]: II. Neurophysiological Characterization and Broad Preclinical Efficacy in Cognition and Schizophrenia of a Potent and Selective Histamine H3 Receptor Antagonist. J. Pharmacol. Exp. Ther. 2005, 313 (1), 176–190. 10.1124/jpet.104.078402. 15608077

[ref25] Medhurst A. D.; Atkins A. R.; Beresford I. J.; Brackenborough K.; Briggs M. A.; Calver A. R.; Cilia J.; Cluderay J. E.; Crook B.; Davis J. B.; Davis R. K.; Davis R. P.; Dawson L. A.; Foley A. G.; Gartlon J.; Gonzalez M. I.; Heslop T.; Hirst W. D.; Jennings C.; Jones D. N. C.; Lacroix L. P.; Martyn A.; Ociepka S.; Ray A.; Regan C. M.; Roberts J. C.; Schogger J.; Southam E.; Stean T. O.; Trail B. K.; Upton N.; Wadsworth G.; Wald J. A.; White T.; Witherington J.; Woolley M. L.; Worby A.; Wilson D. M. GSK189254, a Novel H3 Receptor Antagonist That Binds to Histamine H3 Receptors in Alzheimer’s Disease Brain and Improves Cognitive Performance in Preclinical Models. J. Pharmacol. Exp. Ther. 2007, 321 (3), 1032–1045. 10.1124/jpet.107.120311. 17327487

[ref26] Proschak E.; Stark H.; Merk D. Polypharmacology by Design: A Medicinal Chemist’s Perspective on Multitargeting Compounds. J. Med. Chem. 2019, 62 (2), 420–444. 10.1021/acs.jmedchem.8b00760. 30035545

[ref27] Khanfar M. A.; Affini A.; Lutsenko K.; Nikolic K.; Butini S.; Stark H. Multiple Targeting Approaches on Histamine H3 Receptor Antagonists. Front. Neurosci. 2016, 10 (MAY), 201 10.3389/fnins.2016.00201. 27303254 PMC4884744

[ref28] Lopes F. B.; Aranha C. M. S. Q.; Fernandes J. P. S. Histamine H3 Receptor and Cholinesterases as Synergistic Targets for Cognitive Decline: Strategies to the Rational Design of Multitarget Ligands. Chem. Biol. Drug Des. 2021, 98 (2), 212–225. 10.1111/cbdd.13866. 33991182

[ref29] Kilic B.; Gulcan H. O.; Aksakal F.; Ercetin T.; Oruklu N.; Umit Bagriacik E.; Dogruer D. S. Design and Synthesis of Some New Carboxamide and Propanamide Derivatives Bearing Phenylpyridazine as a Core Ring and the Investigation of Their Inhibitory Potential on In-Vitro Acetylcholinesterase and Butyrylcholinesterase. Bioorg. Chem. 2018, 79, 235–249. 10.1016/j.bioorg.2018.05.006. 29775949

[ref30] Brus B.; Košak U.; Turk S.; Pišlar A.; Coquelle N.; Kos J.; Stojan J.; Colletier J. P.; Gobec S. Discovery, Biological Evaluation, and Crystal Structure of a Novel Nanomolar Selective Butyrylcholinesterase Inhibitor. J. Med. Chem. 2014, 57 (19), 8167–8179. 10.1021/jm501195e. 25226236

[ref31] Li B.; Duysen E. G.; Carlson M.; Lockridge O. The Butyrylcholinesterase Knockout Mouse as a Model for Human Butyrylcholinesterase Deficiency. J. Pharmacol. Exp. Ther. 2008, 324 (3), 1146–1154. 10.1124/jpet.107.133330. 18056867

[ref32] Holmes C.; Ballard C.; Lehmann D.; Smith A. D.; Beaumont H.; Day I. N.; Khan M. N.; Lovestone S.; McCulley M.; Morris C. M.; Munoz D. G.; O’Brien K.; Russ C.; Del Ser T.; Warden D. Rate of Progression of Cognitive Decline in Alzheimer’s Disease: Effect of Butyrylcholinesterase K Gene Variation. J. Neurol. Neurosurg. Psychiatr. 2005, 76 (5), 640–643. 10.1136/jnnp.2004.039321. PMC1739631 15834019

[ref33] Cho W.; Maruff P.; Connell J.; Gargano C.; Calder N.; Doran S.; Fox-Bosetti S.; Hassan A.; Renger J.; Herman G.; Lines C.; Verma A. Additive Effects of a Cholinesterase Inhibitor and a Histamine Inverse Agonist on Scopolamine Deficits in Humans. Psychopharmacology 2011, 218 (3), 513–524. 10.1007/s00213-011-2344-y. 21644059

[ref34] Łażewska D.; Jończyk J.; Bajda M.; Szałaj N.; Więckowska A.; Panek D.; Moore C.; Kuder K.; Malawska B.; Kieć-Kononowicz K. Cholinesterase Inhibitory Activity of Chlorophenoxy Derivatives—Histamine H3 Receptor Ligands. Bioorg. Med. Chem. Lett. 2016, 26 (16), 4140–4145. 10.1016/j.bmcl.2016.04.054. 27445168

[ref35] Bembenek S. D.; Keith J. M.; Letavic M. A.; Apodaca R.; Barbier A. J.; Dvorak L.; Aluisio L.; Miller K. L.; Lovenberg T. W.; Carruthers N. I. Lead Identification of Acetylcholinesterase Inhibitors-Histamine H3 Receptor Antagonists from Molecular Modeling. Bioorg. Med. Chem. 2008, 16 (6), 2968–2973. 10.1016/j.bmc.2007.12.048. 18249544

[ref36] Dorronsoro I.; Castro A.; Martinez A. Peripheral and Dual Binding Site Inhibitors of Acetylcholinesterase as Neurodegenerative Disease Modifying Agents. Expert Opin. Ther. Pat. 2003, 13 (11), 1725–1732. 10.1517/13543776.13.11.1725.

[ref37] Morini G.; Comini M.; Rivara M.; Rivara S.; Bordi F.; Plazzi P. V.; Flammini L.; Saccani F.; Bertoni S.; Ballabeni V.; Barocelli E.; Mor M. Synthesis and Structure-Activity Relationships for Biphenyl H3 Receptor Antagonists with Moderate Anti-Cholinesterase Activity. Bioorg. Med. Chem. 2008, 16 (23), 9911–9924. 10.1016/j.bmc.2008.10.029. 18976927

[ref38] Darras F. H.; Pockes S.; Huang G.; Wehle S.; Strasser A.; Wittmann H. J.; Nimczick M.; Sotriffer C. A.; Decker M. Synthesis, Biological Evaluation, and Computational Studies of Tri- and Tetracyclic Nitrogen-Bridgehead Compounds as Potent Dual-Acting AChE Inhibitors and HH3 Receptor Antagonists. ACS Chem. Neurosci. 2014, 5 (3), 225–242. 10.1021/cn4002126. 24422467 PMC3963125

[ref39] Petroianu G.; Arafat K.; Sasse B. C.; Stark H. Multiple Enzyme Inhibitions by Histamine H3 Receptor Antagonists as Potential Procognitive Agents. Pharmazie 2006, 61, 179. 16599255

[ref40] Staszewski M.; Walczyński K. 1-Phenoxyalkyl-4-[(N,N-Disubstitutedamino)Alkyl]Piperazine Derivatives as Non-Imidazole Histamine H3-Antagonists. Med. Chem. Res. 2013, 22 (3), 1287–1304. 10.1007/s00044-012-0090-2.

[ref41] Staszewski M.; Iwan M.; Werner T.; Bajda M.; Godyń J.; Latacz G.; Korga-Plewko A.; Kubik J.; Szałaj N.; Stark H.; Malawska B.; Więckowska A.; Walczyński K. Guanidines: Synthesis of Novel Histamine H3R Antagonists with Additional Breast Anticancer Activity and Cholinesterases Inhibitory Effect. Pharmaceuticals 2023, 16 (5), 675 10.3390/ph16050675. 37242458 PMC10223552

[ref42] Olszewska B.; Stasiak A.; McNaught Flores D.; Fogel W. A.; Leurs R.; Walczyński K. 4-Hydroxypiperidines and Their Flexible 3-(Amino)Propyloxy Analogues as Non-Imidazole Histamine H3 Receptor Antagonist: Further Structure-Activity Relationship Exploration and in Vitro and in Vivo Pharmacological Evaluation. Int. J. Mol. Sci. 2018, 19 (4), 1243–1261. 10.3390/ijms19041243. 29671795 PMC5979327

[ref43] Masłowska-Lipowicz I.; Walczyński K. Structure-Activity Relationships of New 1-Substitutedmethyl-4-[5-(N-Methyl-N-Propylamino)Pentyloxy]Piperidines and Selected 1-[(N-Substituted-N-Methyl)-3-Propyloxy]-5-(N-Methy-l-N-Propyl)-Pentanediamines as H3-Antagonists. Chem. Biol. Drug Des. 2014, 83 (1), 106–118. 10.1111/cbdd.12206. 23957330

[ref44] Jończyk J.; Malawska B.; Bajda M. Hybrid Approach to Structure Modeling of the Histamine H3 Receptor: Multi-Level Assessment as a Tool for Model Verification. PLoS One 2017, 12 (10), e0186108 10.1371/journal.pone.0186108. 28982153 PMC5629032

[ref45] Benito J. M.; Meldal M. Bicyclic Organo-Peptides as Selective Carbohydrate Receptors: Design, Solid-Phase Synthesis, and on-Bead Binding Capability. QSAR Comb. Sci. 2004, 23 (2–3), 117–129. 10.1002/qsar.200320011.

[ref46] Łukasik B.; Milczarek J.; Pawlowska R.; Żurawiński R.; Chworos A. Facile Synthesis of Fluorescent Distyrylnaphthalene Derivatives for Bioapplications. New J. Chem. 2017, 41 (15), 6977–6980. 10.1039/C7NJ00004A.

[ref47] Vollinga R. C.; Zuiderveld O. P.; Scheerens H.; Bast A.; Timmerman H. A Simple and Rapid in Vitro Test System for the Screening of Histamine H3 Ligands. Methods Find Exp. Clin. Pharmacol. 1992, 14, 747–751. 1338472

[ref48] Kottke T.; Sander K.; Weizel L.; Schneider E. H.; Seifert R.; Stark H. Receptor-Specific Functional Efficacies of Alkyl Imidazoles as Dual Histamine H3/H4 Receptor Ligands. Eur. J. Pharmacol. 2011, 654 (3), 200–208. 10.1016/j.ejphar.2010.12.033. 21237145

[ref49] Daina A.; Michielin O.; Zoete V. SwissADME: A Free Web Tool to Evaluate Pharmacokinetics, Drug-Likeness and Medicinal Chemistry Friendliness of Small Molecules. Sci. Rep. 2017, 7 (1), 42717–42813. 10.1038/srep42717. 28256516 PMC5335600

[ref50] Veber D. F.; Johnson S. R.; Cheng H. Y.; Smith B. R.; Ward K. W.; Kopple K. D. Molecular Properties That Influence the Oral Bioavailability of Drug Candidates. J. Med. Chem. 2002, 45 (12), 2615–2623. 10.1021/jm020017n. 12036371

[ref51] Stark H.; Sippl W.; Ligneau X.; Arrang J. M.; Ganellin C. R.; Schwartz J. C.; Schunack W. Different Antagonist Binding Properties of Human and Rat Histamine H3 Receptors. Bioorg. Med. Chem. Lett. 2001, 11 (7), 951–954. 10.1016/S0960-894X(01)00090-7. 11294398

[ref52] Nieto-Alamilla G.; Márquez-Gómez R.; García-Gálvez A. M.; Morales-Figueroa G. E.; Arias-Montaño J. A. The Histamine H3 Receptor: Structure, Pharmacology, and Function. Mol. Pharmacol. 2016, 90, 649–673. 10.1124/mol.116.104752. 27563055

[ref53] Hancock A. A.; Esbenshade T. A.; Krueger K. M.; Yao B. B. Genetic and Pharmacological Aspects of Histamine H3 Receptor Heterogeneity. Life Sci. 2003, 73 (24), 3043–3072. 10.1016/j.lfs.2003.06.003. 14550847

[ref54] Muegge I.; Heald S. L.; Brittelli D. Simple Selection Criteria for Drug-like Chemical Matter. J. Med. Chem. 2001, 44 (12), 1841–1846. 10.1021/jm015507e. 11384230

[ref55] Ghose A. K.; Viswanadhan V. N.; Wendoloski J. J. A Knowledge-Based Approach in Designing Combinatorial or Medicinal Chemistry Libraries for Drug Discovery. 1. A Qualitative and Quantitative Characterization of Known Drug Databases. J. Comb. Chem. 1999, 1 (1), 55–68. 10.1021/cc9800071. 10746014

[ref56] Lipinski C. A.; Lombardo F.; Dominy B. W.; Feeney P. J. Experimental and computational approaches to estimate solubility and permeability in drug discovery and development settings 1PII of original article: S0169-409X(96)00423-1. The article was originally published in Advanced Drug Delivery Reviews 23 (1997) 3–25. 1. Adv. Drug Deliv. Rev. 2001, 46 (1–3), 3–26. 10.1016/S0169-409X(00)00129-0. 11259830

[ref57] Ellman G. L.; Courtney K. D.; Andres V.; Featherstone R. M. A New and Rapid Colorimetric Determination of Acetylcholinesterase Activity. Biochem. Pharmacol. 1961, 7 (2), 88–95. 10.1016/0006-2952(61)90145-9. 13726518

[ref58] Peng X.; Yang L.; Liu Z.; Lou S.; Mei S.; Li M.; Chen Z.; Zhang H. Structural Basis for Recognition of Antihistamine Drug by Human Histamine Receptor. Nat. Commun. 2022, 13 (1), 6105–6109. 10.1038/s41467-022-33880-y. 36243875 PMC9569329

[ref59] Arunlakshana O.; Schild H. O. Some Quantitative Uses Of Drug Antagonists. Br. J. Pharmacol. Chemother. 1959, 14 (1), 48–58. 10.1111/j.1476-5381.1959.tb00928.x. 13651579 PMC1481829

[ref60] Yung-Chi C.; Prusoff W. H. Relationship between the Inhibition Constant (KI) and the Concentration of Inhibitor Which Causes 50 per Cent Inhibition (I50) of an Enzymatic Reaction. Biochem. Pharmacol. 1973, 22 (23), 3099–3108. 10.1016/0006-2952(73)90196-2. 4202581

[ref61] Leff P.; Dougall I. G. Further Concerns over Cheng-Prusoff Analysis. Trends Pharmacol. Sci. 1993, 14 (4), 110–112. 10.1016/0165-6147(93)90080-4. 8516953

