# Supplementary material for: 4-Oxypiperidine Ethers as Multiple Targeting Ligands at Histamine H3 Receptors and Cholinesterases
Source: ACS Chem Neurosci. 2024 Mar 5;15(6):1206–18. doi: 10.1021/acschemneuro.3c00800 (PMC10958501; doi:10.1021/acschemneuro.3c00800)
Supplement: Supplementary file 1 — cn3c00800_si_001.pdf [file cn3c00800_si_001.pdf]

Supporting Information

4-Oxypiperidine Ethers as Multiple Targeting Ligands at Histamine H_3 Receptors and Cholinesterases

Beata Michalska*[a], Marek Dzięgielewski[a], Justyna Godyń[b], Tobias Werner[c], Marek Bajda[b], Tadeusz Karcz [d], Katarzyna Szczepańska [d,e], Holger Stark[c], Anna Więckowska[b], Krzysztof Walczyński[a], Marek Staszewski*[a]

[a] Department of Synthesis and Technology of Drugs, Medical University of Lodz, Muszynskiego 1, 90-151 Lodz, Poland.

[b] Department of Physicochemical Drug Analysis, Jagiellonian University Medical College, Medyczna 9, 30–688 Krakow, Poland.

[c] Institute of Pharmaceutical and Medicinal Chemistry, Heinrich Heine University Düsseldorf, Universitaetsstr. 1, 40225 Duesseldorf, Germany.

[d] Department of Technology and Biotechnology of Drugs, Faculty of Pharmacy, Jagiellonian University Medical College, Medyczna 9, 30-688 Krakow, Poland.

[e] Department of Medicinal Chemistry, Maj Institute of Pharmacology, Polish Academy of Sciences, Smetna 12, 31-343 Krakow, Poland.

Table of contents

Entry	Section	Page
1	**Chemical synthesis and data analysis.**	**S2**
2	**NMR spectra.**	**S22**
3	**Pharmacological assay results.**	**S46**
3.1	*Ex vivo* assay for histamine H_3R receptor antagonists on guinea pig ileum.	**S46**
3.2	*h*H_3R radioligand displacement binding assay.	**S47**
3.3	Inhibition of electric eel AChE and equine serum BuChE	**S48**
3.4	H_3R intrinsic activity	**S49**
3.5	Physiochemical parameters	**S51**
4	**References**	**S52**

1. Chemical synthesis and data analysis.

Synthesis of 1-(benzofuran-2-ylmethyl)piperidin-4-ol (6.2)

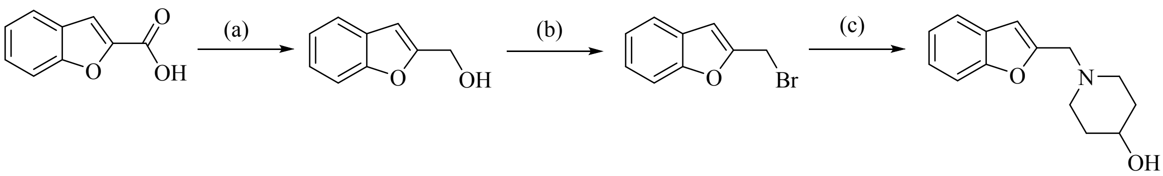

Reagents and conditions: (a) benzofuran-2-carboxylic acid (1 equiv), $LiAlH_4$ (1 equiv), Et_2O, 12h, 0°C→rt; (b) benzofuran-2-ylmethanol (1 equiv), PBr_3 (1 equiv), THF, 12h, 0°C; (c) 2-(bromomethyl)benzofuran (1 equiv), piperidin-4-ol (2 equiv), Cs_2CO_3 (1 equiv), CH_3CN, 10h, 80°C

Benzofuran-2-ylmethanol

$LiAlH_4$ (1 equiv.) was slowly diluted in 50 mL of anhydrous diethyl ether and cooled to 0 °C. To the reaction mixture was added benzofuran-2-carboxylic acid (1 equiv.) diluted in 30 mL of anhydrous diethyl ether. The reaction mixture was stirred at room temperature overnight. After completion, the reaction mixture was quenched by dropwise addition of water (8 equiv.) and 10% NaOH solution (8 equiv.) stirred for 2 h and then filtered by Celite. The precipitate was discarded. The solvent was removed under vacuum, and the crude product was purified by column chromatography to yield the pure product.

(71%): Rf = 0.82 ($CHCl_3$/AcOEt 7:3); **^{1}H-NMR (600MHz, $CDCl_3$):** δ =4.77 (d, 2H, *J*=5.3, CH_2), 6.66 (s, 1H, H^{furan}), 7.20-7.29 (m, 2H, H^{Ar}), 7.46 (d, 1H, *J*=8.2, H^{Ar}), 7.55 (d, 1H, *J*=7.6, H^{Ar})

2-(Bromomethyl)benzofuran

Benzofuran-2-ylmethanol (1 equiv.) was dissolved in THF and cooled to 0 °C. To the reaction mixture was added PBr_3 dropwise (1 equiv.) and the mixture was stirred for 4 h at room temperature. After completion, to the reaction mixture water (20 mL) was added and extracted with dichloromethane (3x 20 mL). The organic layer was dried over Na_2SO_4, the solvent was removed under vacuum, and the crude product was added to next step.

(99%): Rf = 0.90 (CH_2Cl_2/MeOH/NH_3(aq) 8:1:1%); **^{1}H-NMR (600MHz, $CDCl_3$):** δ =4.60 (bs, 2H, CH_2), 6.74 (s, 1H, H^{furan}), 7.15-7.56 (m, 4H, H^{Ar})

1-(Benzofuran-2-ylmethyl)piperidin-4-ol (6.2)

To a solution of 2-(bromomethyl)benzofuran (1 equiv.) and Cs_2CO_3 (1 equiv.) in 20.0 mL of acetonitrile, piperidin-4-ol was added (1 equiv.). The mixture was stirred at 80 °C for 10 h. Water (20 mL) was then added, and the reaction mixture was extracted with dichloromethane

(3x 20 mL). The organic layer was dried over Na_2SO_4, the solvent was removed under vacuum, and the crude product was purified by column chromatography to give compound as a sticky oil.

(35%): Rf = 0.62 ($CHCl_3$/MeOH 8:1); **^{1}H-NMR (600MHz, $CDCl_3$):** δ =1.60-1.68 (m, 2H, CH_2^{pip}), 1.79 (bs, 1H, OH), 1.88-1.94 (m, 2H, CH_2^{pip}), 2.24-2.31 (m, 2H, CH_2^{pip}), 2.80-2.87 (m, 2H, CH_2^{pip}), 3.66-3.73 (m, 3H, CH^{pip}, $CH_2C_8H_5O$), 6.58 (s, 1H, H^{furan}), 7.18-7.27 (m, 2H, H^{Ar}), 7.47 (d, 1H, *J*=8.2, H^{Ar}), 7.52 (d, 1H, *J*=7.5, H^{Ar})

Synthesis of *tert*-butyl 4-hydroxypiperidine-1-carboxylate

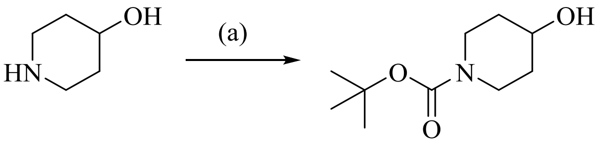

Reagents and contitions: (a) piperidin-4-ol (1 equiv), BOC_2O (2 equiv), THF, Et_3N, 18h, rt.

tert-Butyl 4-hydroxypiperidine-1-carboxylate was obtained according to the literature procedure [1].

(90%): Rf = 0.53 (EtOAc/Hexane 2:1); **^{1}H-NMR (600MHz, $CDCl_3$):** δ =1.42-1.48 (m, 11H, $(CH_3)_3$, CH_2^{pip}), 1.77 (s, 1H, OH), 1.81-1.88 (m, 2H, CH_2^{pip}), 3.00-3.06 (m, 2H, CH_2^{pip}), 3.79-3.87 (m, 3H, CH_2^{pip} ,CH^{pip})

Synthesis of benzofuran-2-yl(4-hydroxypiperidin-1-yl)methanone

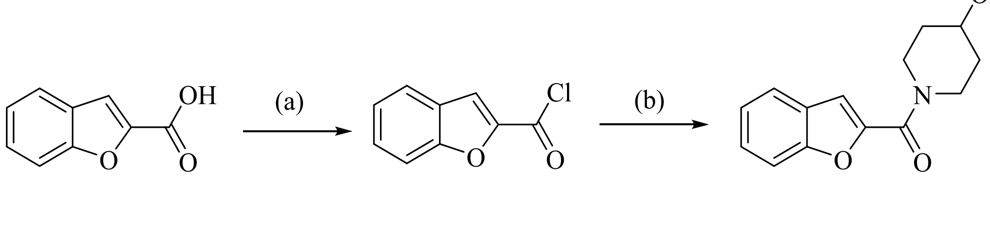

Reagents and conditions: (a) benzofuran-2-carboxylic acid (1 equiv), $SOCl_2$ (5 equiv), toluene, 3h, reflux; (b) benzofuran-2-carbonyl chloride (1 equiv), piperidin-4-ol (1 equiv), Et_3N (3 equiv), CH_2Cl_2, 12h, rt.

Benzofuran-2-yl(4-hydroxypiperidin-1-yl)methanone:

To a solution of piperidin-4-ol (1 equiv.) in dichloromethane (30 mL) excess of Et_3N (3 equiv.) was added dropwise. To this reaction mixture benzofuran-2-carbonyl chloride [2] was

added (1 equiv.) and the mixture was stirred for 12 h at room temperature. The solvent was removed by evaporation, and the residue was dissolved in dichloromethane (20 mL) and washed with K_2CO_3 (aq) (20 mL). The organic layers were dried over $MgSO_4$ and filtered. The solvent was evaporated and the residue was purified by silica gel-flash column chromatography ($CHCl_3$/MeOH 8:1) to afford compound as a white solid.

(90%): Rf = 0.79 ($CHCl_3$/MeOH 8:1); **^{1}H-NMR (600MHz, $CDCl_3$):** δ =1.62-1.69 (m, 2H, CH_2^{pip}), 1.84-1.90 (s, 1H, OH), 1.94-2.04 (m, 2H, CH_2^{pip}), 3.39-3.62 (m, 2H, CH_2^{pip}), 4.00-4.21 (m, 2H, CH^{pip}), 4.14-4.21 (m, 2H, CH_2^{pip}), 7.25-7.31 (m, 2H, H^{Ar}), 7.36-7.42 (m, 1H, H^{Ar}), 7.49-7.56 (m, 1H, H^{Ar}), 7.63-7.68 (m, 1H, H^{Ar}); **^{13}C-NMR ($CDCl_3$, 150 MHz):** δ =67.24 (C^{pip}), 111.80, 112.06, 122.40, 123.77, 126.58, 127.19, 149.30, 154.74 (C^{Ar}), 160.10 (CO)

Synthesis of benzofuran-2-carbaldehyde

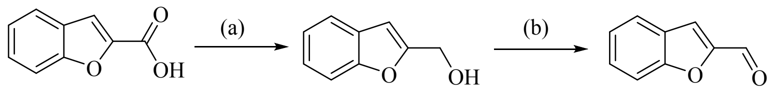

Reagents and conditions: (a) benzofuran-2-carboxylic acid (1 equiv), $LiAlH_4$ (1 equiv), Et_2O, 12h, 0°C→rt; (b) benzofuran-2-ylmethanol (1 equiv), MnO_2 (10 equiv), CH_2Cl_2, 12h, argon atmosphere, rt.

Benzofuran-2-ylmethanol:

Benzofuran-2-ylmethanol was obtained according to the literature procedure [3].

(71%): Rf = 0.82 ($CHCl_3$/AcOEt 7:3); **^{1}H-NMR (600MHz, $CDCl_3$):** δ =4.77 (d, 2H, *J*=5.3, CH_2), 6.66 (s, 1H, H^{furan}), 7.20-7.29 (m, 2H, H^{Ar}), 7.46 (d, 1H, *J*=8.2, H^{Ar}), 7.55 (d, 1H, *J*=7.6, H^{Ar})

Benzofuran-2-carbaldehyde

Benzofuran-2-carbaldehyde was obtained according to the literature procedure and carried directly to the next transformation without further purification [4].

(98%): Rf = 0.70 (CH_2Cl_2/CH_3OH 8:1); **^{1}H-NMR (600MHz, $CDCl_3$):** δ =7.35 (t, 1H, *J*=7.2, CH^{Ar}), 7.53 (t, 1H, *J*=7.2, CH^{Ar}), 7.57 (bs, 1H, CH^{Ar}), 7.60-7.63 (m, 1H, CH^{Ar}), 7.74-7.77 (m, 1H, CH^{Ar}), 9.88 (s, 1H, CHO)

Synthesis of compound 2

4-(Methylamino)phenol hemisulfate salt (1 equiv.) and triethylamine (2 equiv.) were dissolved in 20 mL of methanol. Boc_2O (1.5 equiv.) was added, and the mixture was stirred for 4 h at room temperature. The solvent was removed by evaporation, and the residue was dissolved in dichloromethane (20 mL) and washed with brine (20 mL). The organic layers were dried over $MgSO_4$ and filtered. The solvent was evaporated and the residue was purified by silica gel-flash column chromatography (CH_2Cl_2/MeOH 19:1) to afford compound **1** as a white solid.

tert-butyl (4-hydroxyphenyl)(methyl)carbamate (**2**):

(97%): Rf = 0.64 (CH_2Cl_2/MeOH 19:1); Melting temp. 134-136ºC; **^{1}H-NMR ($CDCl_3$, 600MHz):**δ =0.45 (s, 9H, $(CH_3)_3$), 3.19 (s, 3H, CH_3), 6.67 (bs, 2H, H^{Ar}), 6.99 (d, 2H, *J*=8.8, H^{Ar}); **^{13}C-NMR ($CDCl_3$, 150 MHz,):** δ =28.61 ($C(CH_3)_3$), 38.23 ($C(CH_3)_3$), 116.05, 127.37 (C^{Ar}), 154.62 (C^{Ar}-OH), 156.06 (C=O)

Synthesis of compounds 3.1, 3.2

Diethyl azodicarboxylate (1.3 equiv.) was added dropwise to a stirred solution of 1-benzyl-4-hydroxypiperidine or 1-(benzofuran-2-ylmethyl)piperidin-4-ol (1.3 equiv.), triphenylphosphine (1.3 equiv.) and compound **2** (1 equiv.) in dry THF (20 mL) at 0 °C under argon atmosphere. The reaction mixture was stirred at 0 °C for 10 h. The solvent was removed by evaporation, and the residue was dissolved in ethyl acetate and washed with a saturated $NaHCO_3$ solution. The organic layers were dried over $MgSO_4$ and filtered. The solvent was evaporated and the residue was purified by silica gel-flash column chromatography to afford **3.1** (18%) and **3.2** (58%) as a yellow oil.

tert-butyl (4-((1-benzylpiperidin-4-yl)oxy)phenyl)(methyl)carbamate (**3.1**):

(18%): Rf = 0.33 ($CHCl_3$/EtOAc 7:3); **^{1}H-NMR ($CDCl_3$, 600MHz):**δ =1.42 (s, 9H, $(CH_3)_3$), 1.77-1.83 (m, 2H, H^{pip}), 1.93-1.99 (m, 2H, H^{pip}), 2.24-2.31 (m, 2H, H^{pip}), 2.69-2.75 (m, 2H, H^{pip}), 3.20 (s, 3H, CH_3), 3.52 (s, 2H, CH_2-C_6H_5), 4.23-4.28 (m, 1H, H^{pip}), 6.28-6.85 (m, 2H, H^{Ar}), 7.08-7.10 (m, 2H, H^{Ar}), 7.23-7.25 (m, 1H, H^{Ar}), 7.29-7.33 (m, 4H, H^{Ar}); **^{13}C-NMR ($CDCl_3$, 150 MHz):**δ =28.59 ($C(CH_3)_3$), 31.17 (CH_3), 37.83 (C^{pip}), 50.79 (C^{pip}), 63.26 (CH_2-C_6H_5), 77.15 (C^{pip}), 80.19 ($C(CH_3)_3$), 116.39, 127.10, 127.22, 128.43, 129.30, 137.22, 138.56 (C^{Ar}), 155.35 (C=O)

tert-butyl (4-((1-(benzofuran-2-ylmethyl)piperidin-4-yl)oxy)phenyl)(methyl)carbamate (**3.2**): (58%): Rf = 0.40 (Hexane/EtOAc 7:3); **^{1}H-NMR ($CDCl_3$, 600MHz):**δ =1.42 (bs, 9H, $(CH_3)_3$), 1.82-1.90 (m, 2H, CH_2^{pip}), 1.97-2.04 (m, 2H, CH_2^{pip}), 2.41-2.46 (m, 2H, CH_2^{pip}), 2.78-2.83 (m, 2H, CH_2^{pip}), 3.20 (s, 3H, CH_3), 3.71 (s, 2H, CH_2), 4.26-4.30 (m, 1H, CH^{pip}), 6.81-6.85 (m, 2H, H^{Ar}), 7.06-7.13 (m, 2H, H^{Ar}), 7.18-7.26 (m, 2H, H^{Ar}), 7.46-7.53 (m, 2H, H^{Ar}); **^{13}C-NMR (150 MHz, $CDCl_3$):** δ =28.57 ($C(\mathit{C}H_3)_3$), 30.91 (CH_2^{pip}), 50.61 (CH_2^{pip}), 55.70 (CH_2), 72.88 (CH^{pip}), 80.20 ($\mathit{C}(CH_3)_3$), 105.77 (CH^{furan}), 116.36, 120.90, 122.86, 124.12, 127.10, 128.52, 137.27, 154.98 (C^{Ar}), 155.29 (C=O)

Synthesis of compounds 4.1, 4.2

To a solution of appropriate carbamate (**3.1**, **3.2**) (1 equiv.) in dichloromethane (30 mL) excess of trifluoroacetic acid (25 equiv.) was added dropwise. The reaction mixture was stirred overnight at room temperature. After completing the reaction mixture was evaporated, dissolved with dichloromethane (20 mL), and washed with a saturated solution of $NaHCO_3$. The organic layers were dried over $MgSO_4$ and filtered. The solvent was evaporated and the residue was purified by silica gel-flash column chromatography to afford the products as a yellow oil.

4-((1-benzylpiperidin-4-yl)oxy)-N-methylaniline (**4.1**):
(99%): Rf = 0.14 ($CHCl_3$/EtOAc 7:3); **^{1}H-NMR ($CDCl_3$, 600MHz):**δ =1.74-1.82 (m, 2H, H^{pip}), 1.90-1.97 (m, 2H, H^{pip}), 2.20-2.27 (m, 2H, H^{pip}), 2.71-2.77 (m, 2H, H^{pip}), 2.79 (s, 3H, CH_3), 3.52 (s, 2H, $C\mathit{H_2}$-C_6H_5), 4.08-4.13 (m, 1H, H^{pip}), 6.53-6.56 (m, 2H, H^{Ar}), 6.78-6.81 (m, 2H, H^{Ar}), 7.22-7.25 (m, 1H, H^{Ar}), 7.29-7.33 (m, 4H, H^{Ar}); **^{13}C-NMR ($CDCl_3$, 150 MHz):** δ =31.37 (CH_3), 31.72 (C^{pip}), 50.94 (C^{pip}), 63.24 ($\mathit{C}H_2$-C_6H_5), 113.69, 118.71, 127.18, 128.40, 129.32, 144.36, 149.72 (C^{Ar})

4-((1-(benzofuran-2-ylmethyl)piperidin-4-yl)oxy)-N-methylaniline (**4.2**):
(80%): Rf = 0.20 ($CHCl_3$/EtOAc 7:3); **^{1}H-NMR ($CDCl_3$, 600MHz):**δ =1.77-1.83 (m, 2H, CH_2^{pip}), 1.90-1.95 (m, 2H, CH_2^{pip}), 2.29-2.37 (m, 2H, CH_2^{pip}) , 2.68 (s, 3H, CH_3), 2.74-2.79 (m, 2H, CH_2^{pip}), 3.56 (s, 1H, NH), 3.63 (s, 2H, CH_2), 4.05-4.09 (m, 1H, CH^{pip}), 6.47 (d, 2H, *J*=8.9, H^{Ar}), 6.53 (s, 1H, CH^{furan}), 6.76 (d, 2H, *J*=8.9, H^{Ar}), 7.14-7.21 (m, 2H, H^{Ar}), 7.46 (dd, 2H, *J*=7.6, 21.06, H^{Ar}); **^{13}C-NMR (150 MHz, $CDCl_3$):** δ =30.78 (CH_3), 31.23 (CH_2^{pip}), 50.37 (CH_2^{pip}), 55.24 (CH_2), 73.67 (CH^{pip}), 105.39 (CH^{furan}), 111.13, 113.25, 118.24, 120.57, 122.53, 123.77, 128.22, 144.11, 149.16, 154.70 (C^{Ar})

General procedure for the preparation of compounds 9a.1, 9b.1, 9c.1, 9a.2, 9b.2, 9c.2.

$LiAlH_4$ (2 equiv.) was slowly added to a solution of the corresponding nitrile (1 equiv.) (**8a.1, 8b.1, 8c.1, 8a.2, 8b.2, 8c.2**) in 50 mL of anhydrous diethyl ether. The reaction mixture was stirred at 36 °C for 3 h. After completion, the reaction mixture was quenched by dropwise addition of water (8 equiv.) and 10% NaOH solution (8 equiv.) stirred for 2 h and then filtered by Celite. The precipitate was discarded. The solvent was removed under vacuum, and the crude product was purified by column chromatography to yield the pure product.

(4-(((1-Benzylpiperidin-4-yl)oxy)methyl)phenyl)methanamine (**9c.1**):
(96%): Rf = 0.50 ($CH_2Cl_2/MeOH/NH_{3(aq)}$ 8:1:1%); **^{1}H-NMR ($CDCl_3$, 600MHz):**δ = 1.65-1.71 (m, 2H, CH_2^{pip}), 1.88-1.92 (m, 2H, CH_2^{pip}), 2.13-2.18 (m, 2H, CH_2^{pip}), 2.72-2.77 (m, 2H, CH_2^{pip}), 3.38-3.43 (m, 1H, CH^{pip}), 3.49 (s, 2H, $CH_2C_6H_5$), 3.84 (s, 2H, $C\underline{H_2}NH_2$), 4.51 (s, 2H, $OC\underline{H_2}C_6H_5$), 7.22-7.31 (m, 9H, HAr); **^{13}C-NMR (150 MHz, $CDCl_3$):** δ =31.39 (C^{pip}), 46.36 ($\underline{C}H_2NH_2$), 51.21 (C^{pip}), 63.16 ($\underline{C}H_2C_6H_5$), 69.63 ($O\underline{C}H_2C_6H_4$), 74.55 (C^{pip}), 127.19, 127.32, 127.98, 128.36, 129.35, 137.81, 138.47, 142.46 (C^{Ar})
(4-(((1-(Benzofuran-2-ylmethyl)piperidin-4-yl)oxy)methyl)phenyl)methanamine (**9c.2**):
(50%): Rf = 0.51 ($CH_2Cl_2/MeOH/NH_{3(aq)}$ 8:1:1%); **^{1}H-NMR ($CDCl_3$, 600MHz):**δ =1.70-1.76 (m, 2H, CH_2^{pip}), 1.89-1.95 (m, 2H, CH_2^{pip}), 2.26-2.33 (m, 2H, CH_2^{pip}), 2.79-2.85 (m, 2H, CH_2^{pip}), 3.39-3.44 (m, 1H, CH^{pip}), 3.68 (s, 2H, $C\mathit{H_2}NC_5H_9$), 3.84 (s, 2H, $\mathit{C}H_2NH_2$), 4.50 (s, 2H, $OC\mathit{H_2}C_6H_4$), 6.57 (s, 1H, CH^{furan}), 7.17-7.31 (m, 7H, CH^{Ar}), 7.46 (d, 1H, *J*=8.1, CH^{Ar}), 7.51 (d, 1H, *J*=8.1, CH^{Ar}); **^{13}C-NMR (150 MHz, $CDCl_3$):** δ =31.24 ($2xCH_2^{pip}$), 43.10 ($\mathit{C}H_2NH$), 46.07 ($2xCH_2^{pip}$), 57.90 ($\mathit{C}H_2NC_5H_9$), 66.95 ($O\mathit{C}H_2C_6H_5$), 69.62 (CH^{pip}), 105.77 (C^{furan}), 111.49, 120.87, 122.83, 124.07, 127.54, 128.00, 128.51, 137.98, 154.93 (C^{Ar})

(3-(((1-Benzylpiperidin-4-yl)oxy)methyl)phenyl)methanamine (**9b.1**):
(62%): Rf = 0.28 ($CH_2Cl_2/MeOH/NH_{3(aq)}$ 8:1:1%); **^{1}H-NMR ($CDCl_3$, 600MHz):**δ =1.47 (bs, 2H, NH_2), 1.65-1.71 (m, 2H, CH_2^{pip}), 1.89-1.95 (m, 2H, CH_2^{pip}), 2.10-2.16 (m, 2H, CH_2^{pip}), 2.72-2.79 (m, 2H, CH_2^{pip}), 3.39-3.44 (m, 1H, CH^{pip}), 3.49 (s, 2H, $C_6H_5C\mathit{H_2}$), 3.86 (s, 2H, $OC\mathit{H_2}C_6H_4$), 4.53 (s, 2H, $C\mathit{H_2}NH_2$) 7.19-7.26 (m, 3H, CH^{Ar}), 7.28-7.33 (m, 6H, CH^{Ar}); **^{13}C-NMR (150 MHz, $CDCl_3$):** δ =31.55 ($2xC^{pip}$), 46.66 ($\mathit{C}H_2NH_2$), 51.37 ($2xC^{pip}$), 63.20 ($C_6H_5\mathit{C}H_2$), 69.85 ($O\mathit{C}H_2C_6H_4$ and C^{pip}), 126.18, 126.39, 127.12, 128.35, 128.76, 129.28, 138.76, 139.48, 143.66 (C^{Ar})

(3-(((1-(Benzofuran-2-ylmethyl)piperidin-4-yl)oxy)methyl)phenyl)methanamine (**9b.2**): (62%): Rf = 0.20 (CH_2Cl_2/MeOH/$NH_{3(aq)}$ 8:1:1%); **^{1}H-NMR ($CDCl_3$, 600MHz):** δ =1.71-1.78 (m, 2H, CH_2^{pip}), 1.91-1.98 (m, 2H, CH_2^{pip}), 2.24-2.33 (m, 2H, CH_2^{pip}), 2.80-2.86 (m, 2H, CH_2^{pip}), 3.39-3.46 (m, 1H, CH^{pip}), 3.68 (s, 2H, $CH_2NC_5H_9$), 3.85 (s, 2H,CH_2NH_2), 4.52 (s, 2H, $OCH_2C_6H_4$), 6.58 (s, 1H, CH^{furan}), 7.18-7.33 (m, 6H, CH^{Ar}), 7.47 (d, 1H, *J*=8.2, CH^{Ar}), 7.51 (d, 1H, *J*=7.4, CH^{Ar}); **^{13}C-NMR (150 MHz, $CDCl_3$):** δ =31.28 (2xC^{pip}), 46.62 (CH_2NH_2), 51.18 (2xC^{pip}), 55.61 ($CH_2NC_5H_9$), 69.84 ($OCH_2C_6H_5$), 72.87 (C^{pip}), 105.66 (C^{furan}), 111.47, 120.82, 122.78, 124.02, 126.44, 128.74, 139.36, 143.61 155.00 (C^{Ar})

(2-(((1-Benzylpiperidin-4-yl)oxy)methyl)phenyl)methanamine (**9a.1**): (59%): Rf = 0.64 (CH_2Cl_2/MeOH/$NH_{3(aq)}$ 8:1:1%); **^{1}H-NMR ($CDCl_3$, 600MHz):** δ =1.58-1.68 (m, 2H, CH_2^{pip}), 1.86-1.93 (m, 2H, CH_2^{pip}), 1.99 (bs, 2H, NH_2), 2.06-2.14 (m, 2H, CH_2^{pip}), 2.66-2.75 (m, 2H, CH_2^{pip}), 3.38-3.46 (m, 3H, CH^{pip}, CH_2-C_6H_5), 3.81 (s, 2H, CH_2NH_2), 4.50 (s, 2H, $OCH_2C_6H_4$), 7.15-7.38 (m, 9H, H^{Ar}); **^{13}C-NMR ($CDCl_3$, 150MHz):** δ =31.45 (C^{pip} 2x), 44.15 (CH_2NH_2), 51.27 (C^{pip} 2x), 63.09 ($CH_2C_6H_5$), 68.64 ($OCH_2C_6H_5$), 75.34 (C^{pip}), 127.13, 128.31, 128.58, 129.24, 129.95, 136.05, 138.57, 142.56 (C^{Ar})

(2-(((1-(Benzofuran-2-ylmethyl)piperidin-4-yl)oxy)methyl)phenyl)methanamine (**9a.2**): (27%): Rf = 0.23 (CH_2Cl_2/MeOH/$NH_{3(aq)}$ 8:1:1%); **^{1}H-NMR ($CDCl_3$, 600MHz):** δ =1.69-1.79 (m, 4H, NH_2, CH_2^{pip}), 1.93-2.01 (m, 2H, CH_2^{pip}), 2.26-2.33 (m, 2H, CH_2^{pip}), 2.79-2.87 (m, 2H, CH_2^{pip}), 3.45-3.50 (m, 1H, CH^{pip}), 3.68 (s, 2H, $CH_2C_8H_5O$), 3.86 (s, 2H, CH_2NH_2), 4.56 (s, 2H, $OCH_2C_6H_4$), 6.57 (s, 1H, H^{furan}), 7.18-7.35 (m, 6H, H^{Ar}), 7.46-7.53 (m, 6H, H^{Ar}); **^{13}C-NMR ($CDCl_3$, 150MHz):** δ =31.30 (C^{pip} 2x), 44.24 (CH_2NH_2), 51.19 (C^{pip} 2x), 55.63 ($CH_2C_8H_5O$), 66.16 ($OCH_2C_6H_4$), 74.85 (C^{pip}), 105.72 (C^{furan}),111.51, 120.86, 124.06, 128.48, 128.54, 129.91, 136.02, 142.71 (C^{Ar})

General procedure for preparation of compounds 10a.1, 10b.1, 10c.1, 10a.2, 10b.2, 10c.2.

To a corresponding amine (**9a.1, 9b.1, 9c.1, 9a.2, 9b.2, 9c.2**) (1 equiv.) excess of methyl formate was added (10 equiv.). The reaction mixture was stirred at room temperature for 10 h. The solvent was evaporated and the residue was purified by silica gel-flash column chromatography to yield the pure product.

N-(4-(((1-Benzylpiperidin-4-yl)oxy)methyl)benzyl)formamide (**10c.1**):

(78%): Rf = 0.54 (CH_2Cl_2/MeOH/$NH_{3(aq)}$ 8:1:1%); **^{1}H-NMR ($CDCl_3$, 600MHz):**δ =1.63-1.70 (m, 2H, CH_2^{pip}), 1.85-1.91 (m, 2H, CH_2^{pip}), 2.10-2.16 (m, 2H, CH_2^{pip}), 2.71-2.77 (m, 2H, CH_2^{pip}), 3.37-3.42 (m, 1H, CH^{pip}), 3.48 (s, 2H, $CH_2C_6H_5$), 4.38 (d, 0.3H, *J*=6.5, $C\underline{H_2}N$), 4.51 (d, 1.7H, *J*=6.5, $C\underline{H_2}N$), 4.51 (s, 2H, $OC\underline{H_2}C_6H_5$), 5.84 (bs, 1H, NH), 7.20-7.34 (m, 9H, HAr), 8.23 (s, 1H, CHO); **^{13}C-NMR (150 MHz, $CDCl_3$):** δ =31.57 (C^{pip}), 42.19 ($\underline{C}H_2N$), 51.31 (C^{pip}), 63.21 ($\underline{C}H_2C_6H_5$), 69.43 ($O\underline{C}H_2C_6H_4$), 74.93 (C^{pip}), 127.15, 127.22, 128.13, 128.38, 129.27, 136.94, 138.92 (C^{Ar}), 161.06 (CO)

N-(4-(((1-(Benzofuran-2-ylmethyl)piperidin-4-yl)oxy)methyl)benzyl)formamide (**10c.2**):

(70%): Rf = 0.90 (CH_2Cl_2/MeOH/$NH_{3(aq)}$ 8:1:1%); **^{1}H-NMR ($CDCl_3$, 600MHz):**δ =1.68-1.76 (m, 2H, CH_2^{pip}), 1.88-1.95 (m, 2H, CH_2^{pip}), 2.24-2.32 (m, 2H, CH_2^{pip}), 2.79-2.85(m, 2H, CH_2^{pip}), 3.36-3.45 (m, 1H, CH^{pip}), 3.67 (s, 2H, $OCH_2C_6H_4$), 4.43 (d, 0.5H *J*=5.9, $NHCH_2$), 4.46 (d, 1.5H *J*=5.9, $NHCH_2$), 4.50 (s, 2H, $CH_2NC_5H_9$), 6.56 (s, 1H, CH^{furan}), 7.17-7.33 (m, 7H, CH^{Ar}), 7.46 (d, 1H, *J*=8.0, CH^{Ar}), 7.51 (d, 1H, *J*=8.0, CH^{Ar}); **^{13}C-NMR (150 MHz, $CDCl_3$):** δ =31.32 (2xCH_2^{pip}), 42.25 (CH_2NH)), 51.17 (2xCH_2^{pip}), 55.68 ($OCH_2C_6H_5$, $CH_2NC_5H_9$), 69.53 (CH^{pip}), 105.66 (C^{furan}), 111.45, 120.86, 122.87, 124.08, 128.16, 128.56, 138.96, 138.93, 155.33 (C^{Ar}), 161.02 (CO)

N-(3-(((1-Benzylpiperidin-4-yl)oxy)methyl)benzyl)formamide (**10b.1**):

(99%): Rf = 0.68 (CH_2Cl_2/MeOH/$NH_{3(aq)}$ 8:1:1%); **^{1}H-NMR ($CDCl_3$, 600MHz):**δ =1.63-1.72 (m, 2H, CH_2^{pip}), 1.87-1.94 (m, 2H, CH_2^{pip}), 2.07-2.19 (m, 2H, CH_2^{pip}), 2.71-2.80 (m, 2H, CH_2^{pip}), 3.37-3.43 (m, 1H, CH^{pip}), 3.50 (s, 2H, $C_6H_5CH_2$), 4.41 (d, 0.4H, *J*=6.5, CH_2NHCO), 4.47 (d, 1.6H, *J*=6.5, CH_2NHCO), 4.52 (s, 2H, $OCH_2C_6H_4$), 5.80 (bs, 1H, NH), 7.18-7.39 (m, 9H, CH^{Ar}), 8.25 (bs, 1H, CHO); **^{13}C-NMR (150 MHz, $CDCl_3$):** δ =31.52 (2xC^{pip}), 42.36 (CH_2NH), 49.79 (C^{pip}), 51.35 (C^{pip}), 63.18 ($C_6H_5CH_2$), 69.71 ($OCH_2C_6H_4$), 75.10 (C^{pip}), 126.21, 127.11, 127.16, 127.23, 128.42, 129.08, 129.36, 137.85, 139.89 (C^{Ar}), 161.15 (C=O)

N-(3-(((1-(Benzofuran-2-ylmethyl)piperidin-4-yl)oxy)methyl)benzyl)formamide (**10b.2**):

(84%): Rf = 0.82 (CH_2Cl_2/MeOH/$NH_{3(aq)}$ 8:1:1%); **^{1}H-NMR ($CDCl_3$, 600MHz):**δ =1.70-1.78 (m, 2H, CH_2^{pip}), 1.90-1.99 (m, 2H, CH_2^{pip}), 2.22-2.33 (m, 2H, CH_2^{pip}), 2.78-2.88(m, 2H, CH_2^{pip}), 3.39-3.46 (m, 1H, CH^{pip}), 3.68 (s, 2H, $OCH_2C_6H_4$), 4.45-4.51 (m, 4H $NHCH_2$, $CH_2NC_5H_9$), 5.94 (bs, 1H, NH), 6.58 (s, 1H, CH^{furan}), 7.17-7.34 (m, 6H, CH^{Ar}), 7.47 (d, 1H, *J*=8.1, CH^{Ar}), 7.52 (d, 1H, *J*=7.6, CH^{Ar}), 8.24 (s, 1H, CHO); **^{13}C-NMR (150 MHz, $CDCl_3$):**

δ =31.26 (2xC^{pip}), 42.28 (CH_2NH)), 51.15 (2xC^{pip}), 55.59 ($OCH_2C_6H_5$), 69.68 ($CH_2NC_5H_9$), 74.59 (C^{pip}), 105.74 (C^{furan}), 111.48, 120.85, 122.82, 124.07, 127.21, 129.03, 137.88, 155.22 (C^{Ar}), 171.38 (CO)

N-(2-(((1-Benzylpiperidin-4-yl)oxy)methyl)benzyl)formamide (**10a.1**):
(87%): Rf = 0.75 (CH_2Cl_2/MeOH/$NH_{3(aq)}$ 8:1:1%); **^{1}H-NMR ($CDCl_3$, 600MHz):** δ =1.62-1.72 (m, 2H, CH_2^{pip}), 1.92-1.99 (m, 2H, CH_2^{pip}), 2.13-2.20 (m, 2H, CH_2^{pip}), 2.74-2.80 (m, 2H, CH_2^{pip}), 3.47-3.53 (m, 3H, CH^{pip}, CH_2-C_6H_5), 4.51 (s, 2H, CH_2NH_2), 4.55 (s, 2H, $OCH_2C_6H_4$), 7.23-7.38 (m, 9H, H^{Ar}), 8.14 (s, 1H, CHO)***;*** **^{13}C-NMR ($CDCl_3$, 150MHz):**δ =31.45 (C^{pip} 2x), 40.07 (CH_2NH), 51.21 (C^{pip} 2x), 63.06 ($CH_2C_6H_5$), 69.11 ($OCH_2C_6H_5$), 75.71 (C^{pip}), 127.29, 128.24, 128.42, 129.12, 129.32, 130.34, 136.63, 137.17 (C^{Ar}), 160.65 (CHO)

N-(2-(((1-(Benzofuran-2-ylmethyl)piperidin-4-yl)oxy)methyl)benzyl)formamide (**10a.2**):

(84%): Rf = 0.82 (CH_2Cl_2/MeOH/NH_3(aq) 8:1:1%); **^{1}H-NMR ($CDCl_3$, 600MHz):**δ =1.70-1.77 (m, 2H, CH_2^{pip}), 1.94-2.02 (m, 2H, CH_2^{pip}), 2.25-2.33 (m, 2H, CH_2^{pip}), 2.80-2.88 (m, 2H, CH_2^{pip}), 3.46-3.53 (m, 1H, CH^{pip}), 3.68 (s, 2H, $CH_2C_8H_5O$), 4.51 (s, 2H, CH_2NH), 4.55 (s, 2H, $OCH_2C_6H_4$), 6.58 (s, 1H, H^{furan}), 7.18-7. 53 (m, 8H, H^{Ar}), 8.14 (s, 1H, CHO)*;* **^{13}C-NMR ($CDCl_3$, 150MHz):** δ =31.38 (C^{pip} 2x), 40.06 (CH_2NH), 51.20 (C^{pip} 2x), 55.53 ($CH_2C_8H_5O$), 69.07 ($OCH_2C_6H_5$), 75.80 (C^{pip}), 105.79 (C^{furan}), 111.48, 120.87, 124.14, 128.18, 130.37, 137.12, 155.23, 160.69 (C^{Ar}), 171.36 (CHO)

General procedure for the preparation of compounds 11a.1, 11b.1, 11c.1, 11a.2, 11b.2, 11c.2

$LiAlH_4$ (2 equiv.) was slowly added to a solution of the corresponding amide (1 equiv.) (**10a.1, 10b.1, 10c.1, 10a.2, 10b.2, 10c.2**) in 50 mL of anhydrous diethyl ether. The reaction mixture was stirred at 36 °C for 3 h. After completion, the reaction mixture was quenched by dropwise addition of water (8 equiv.) and 10% NaOH solution (8 equiv.) stirred for 2 h and then filtered by Celite. The precipitate was discarded. The solvent was removed under vacuum, and the crude product was purified by column chromatography to yield the pure product.

1-(4-(((1-Benzylpiperidin-4-yl)oxy)methyl)phenyl)-N-methylmethanamine (**11c.1**):
(71%): Rf = 0.84 (CH_2Cl_2/MeOH/$NH_{3(aq)}$ 8:1:1%); **^{1}H-NMR ($CDCl_3$, 600MHz):** δ =1.65-1.71 (m, 2H, CH_2^{pip}), 1.87-1.93 (m, 2H, CH_2^{pip}), 2.12-2.17 (m, 2H, CH_2^{pip}), 2.44 (s, 3H, CH_3), 2.73-2.76 (m, 2H, CH_2^{pip}), 3.39-3.43 (m, 1H, CH^{pip}), 3.49 (s, 2H, $CH_2C_6H_5$), 3.74 (s, 2H, $C\mathit{H_2}N$), 4.52 (s, 2H, $OC\mathit{H_2}C_6H_5$), 7.22-7.31 (m, 9H, H^{Ar}); **^{13}C-NMR (150 MHz, $CDCl_3$):** δ =31.52 (C^{pip}), 36.07 (CH_3), 51.32 ($\mathit{C}H_2N$), 55.94 (C^{pip}), 63.21 ($\mathit{C}H_2C_6H_5$), 69.69 (O $\mathit{C}H_2C_6H_4$), 74.68 (C^{pip}), 127.18, 127.84, 128.13, 128.40, 128.46, 128.51, 128.54, 129.33, 138.06 (C^{Ar})

1-(4-(((1-(Benzofuran-2-ylmethyl)piperidin-4-yl)oxy)methyl)phenyl)-N-methylmethanamine (**11c.2**):
(90%): Rf = 0.54 (CH_2Cl_2/MeOH/$NH_{3(aq)}$ 8:1:1%); **^{1}H-NMR ($CDCl_3$, 600MHz):** δ = 1.68-1.77 (m, 2H, CH_2^{pip}), 1.89-1.95 (m, 2H, CH_2^{pip}), 2.26-2.31 (m, 2H, CH_2^{pip}), 2.44 (s, 3H, CH_3), 2.78-2.84 (m, 2H, CH_2^{pip}), 3.39-3.44 (m, 1H, CH^{pip}), 3.67 (s, 2H, $C_6H_4C\mathit{H_2}N$), 3.73 (s, 2H, $C\mathit{H_2}NC_5H_9$), 4.50 (s, 2H, $OC\mathit{H_2}C_6H_4$), 6.56 (s, 1H, CH^{furan}), 7.17-7.30 (m, 6H, CH^{Ar}), 7.45-7.52 (m, 2H, CH^{Ar}); **^{13}C-NMR (150 MHz, $CDCl_3$):** δ =31.34 (2xCH_2^{pip}), 35.93 (CH_3), 51.17 (2xCH_2^{pip}), 55.65 ($C_6H_4\mathit{C}H_2N$, $\mathit{C}H_2NC_5H_9$), 69.67 ($O\mathit{C}H_2C_6H_4$), 74.17 (CH^{pip}), 105.68 (C^{furan}), 111.49, 120.84, 122.81, 124.03, 127.82, 128.47, 138.00, 155.15 (C^{Ar})

1-(3-(((1-Benzylpiperidin-4-yl)oxy)methyl)phenyl)-N-methylmethanamine (**11b.1**):
(77%): Rf = 0.47 (CH_2Cl_2/MeOH/$NH_{3(aq)}$ 8:1:1%); **^{1}H-NMR ($CDCl_3$, 600MHz):** δ =1.64-1.71 (m, 2H, CH_2^{pip}), 1.88-1.96 (m, 2H, CH_2^{pip}), 2.10-2.16 (m, 2H, CH_2^{pip}), 2.45 (s, 3H, CH_3), 2.73-2.78 (m, 2H, CH_2^{pip}), 3.38-3.42 (m, 1H, CH^{pip}), 3.49 (s, 2H, $C_6H_5C\mathit{H_2}$), 3.73 (s, 2H, $C\mathit{H_2}NHCH_3$), 4.52 (s, 2H, $OC\mathit{H_2}C_6H_4$), 7.20-7.31 (m, 9H, CH^{Ar}); **^{13}C-NMR (150 MHz, $CDCl_3$):** δ =31.46 (2xC^{pip}), 36.15 (CH_3), 51.30 ($\mathit{C}H_2NHCH_3$), 56.12 (2xC^{pip}), 63.16 ($C_6H_5\mathit{C}H_2$), 69.81 ($O\mathit{C}H_2C_6H_4$), 74.84 (C^{pip}), 126.39, 127.11, 127.48, 128.32, 128.59, 129.28, 138.62, 139.30, 140.21 (C^{Ar})

1-(3-(((1-(Benzofuran-2-ylmethyl)piperidin-4-yl)oxy)methyl)phenyl)-N-methylmethanamine (**11b.2**):
(78%): Rf = 0.19 (CH_2Cl_2/MeOH/$NH_{3(aq)}$ 8:1:1%); **^{1}H-NMR ($CDCl_3$, 600MHz):** δ =1.69-1.78 (m, 2H, CH_2^{pip}), 1.89-1.96 (m, 2H, CH_2^{pip}), 2.21-2.31 (m, 2H, CH_2^{pip}), 2.44 (s, 3H, CH_3), 2.79-2.87 (m, 2H, CH_2^{pip}), 3.39-3.45 (m, 1H, CH^{pip}), 3.67 (s, 2H, $C_6H_5C\mathit{H_2}$), 3.73 (s, 2H,

CH_2NHCH_3), 4.52 (s, 2H, $OCH_2C_6H_4$), 6.57 (s, 1H, CH^{furan}), 7.18-7.33 (m, 6H, CH^{Ar}), 7.45-7.52 (m, 2H, CH^{Ar}); **^{13}C-NMR (150 MHz, $CDCl_3$):** δ =31.31 (2xC^{pip}), 36.27 (CH_3), 51.20(2xC^{pip}), 55.64 (CH_2NHCH_3), 56.23 ($C_8H_5CH_2$), 69.87 ($OCH_2C_6H_4$), 74.87 (C^{pip}), 105.66 (C^{furan}), 111.49, 120.83, 122.79, 124.03, 126.35, 127.68, 128.61, 139.24, 140.48, 155.05 (C^{Ar})

1-(2-(((1-Benzylpiperidin-4-yl)oxy)methyl)phenyl)-N-methylmethanamine (**11a.1**):
(70%): Rf = 0.30 (CH_2Cl_2/MeOH/$NH_{3(aq)}$ 8:1:1%); **^{1}H-NMR ($CDCl_3$, 600MHz):** δ =1.62-1.71 (m, 2H, CH_2^{pip}), 1.85-1.94 (m, 2H, CH_2^{pip}), 2.10-2.18 (m, 2H, CH_2^{pip}), 2.45 (s, 3H, CH_3), 2.72-2.78 (m, 2H, CH_2^{pip}), 3.42-3.47 (m, 1H, CH^{pip}), 3.48 (s, 2H, CH_2-C_6H_5), 3.76 (s, 2H, CH_2NH_2), 4.57 (s, 2H, $OCH_2C_6H_4$), 7.22-7.35 (m, 9H, H^{Ar})***;*** **^{13}C-NMR ($CDCl_3$, 150MHz):** δ =31.52 (C^{pip} 2x), 36.35 (CH_3), 51.32 (C^{pip} 2x), 53.52 (CH_2NH), 63.12 ($CH_2C_6H_5$), 68.39 ($OCH_2C_6H_5$), 75.38 (C^{pip}), 127.09, 127.28, 128.12, 128.32, 129.22, 129.70, 129.75, 136.83, 138.68, 138.95 (C^{Ar})

1-(2-(((1-(Benzofuran-2-ylmethyl)piperidin-4-yl)oxy)methyl)phenyl)-N-methylmethanamine (**11a.2**): (55%): Rf = 0.45 (CH_2Cl_2/MeOH/$NH_{3(aq)}$ 8:1:1%); **^{1}H-NMR ($CDCl_3$, 600MHz):** δ =**^{1}H-NMR ($CDCl_3$, 600MHz):**1.67-1.78 (m, 2H, CH_2^{pip}), 1.90-1.99 (m, 2H, CH_2^{pip}), 2.25-2.33 (m, 2H, CH_2^{pip}), 2.43 (s, 3H, CH_3), 2.79-2.86 (m, 2H, CH_2^{pip}), 3.43-3.48 (m, 1H, CH^{pip}), 3.67 (s, 2H, $CH_2C_8H_5O$), 3.75 (s, 2H, CH_2NH), 4.56 (s, 2H, $OCH_2C_6H_4$), 6.57 (s, 1H, H^{furan}), 7.17-7. 34 (m, 6H, H^{Ar}), 7.45-7. 52 (m, 2H, H^{Ar})***;*** **^{13}C-NMR ($CDCl_3$, 150MHz):** δ =31.27 (C^{pip} 2x), 36.27 (CH_3), 51.15 (C^{pip} 2x), 53.45 (CH_2NH), 55.57 ($CH_2C_8H_5O$), 68.40 ($OCH_2C_6H_4$), 74.62 (C^{pip}), 105.67 (C^{furan}), 111.44, 120.80, 122.76, 124.01, 127.28, 128.42, 129.73, 138.95, 154.92 (C^{Ar})

General procedure for the preparation of compounds 5.1, 5.2, 12a.1, 12b.1, 12c.1, 12a.2, 12b.2, 12c.2

Propionic anhydride (2 equiv.) was added to a solution of corresponding amine (**4.1, 4.2, 11a.1, 11b.1, 11c.1, 11a.2, 11b.2, 11c.2**) (1 equiv.) in 20.0 mL of dichloromethane. The mixture was stirred at room temperature for 2 h. To the reaction mixture water (20 mL) was added, neutralized with K_2CO_3, and extracted with dichloromethane (3x 20 mL). The organic

layer was dried over Na_2SO_4, the solvent was removed under vacuum, and the crude product was purified by column chromatography to obtain the pure products as a sticky oil.

N-(4-((1-Benzylpiperidin-4-yl)oxy)phenyl)-N-methylpropionamide (**5.1**):
(99%): Rf = 0.26 (Hexane/EtOAc 1:5); **^{1}H-NMR ($CDCl_3$, 600MHz):**δ =1.03 (t, 3H, *J*=7.5, CH_2CH_3), 1.81-1.86 (m, 2H, H^{pip}), 1.98-2.03 (m, 2H, H^{pip}), 2.06 (t, 3H, CH_2CH_3), 2.30-2.35 (m, 2H, H^{pip}), 2.73-2.78 (m, 2H, H^{pip}), 3.21 (s, 3H, CH_3), 3.54 (s, 2H, CH_2-C_6H_5), 4.29-4.33 (m, 1H, H^{pip}), 6.88-6.91 (m, 2H, H^{Ar}), 7.04-7.07 (m, 2H, H^{Ar}), 7.23-7.27 (m, 1H, H^{Ar}), 7.30-7.33 (m, 4H, H^{Ar})

N-(4-((1-(Benzofuran-2-ylmethyl)piperidin-4-yl)oxy)phenyl)-N-methylpropionamide (**5.2**):
(99%): Rf = 0.30 (Hexane/EtOAc 1:5); **^{1}H-NMR ($CDCl_3$, 600MHz):**δ =1.03 (t, 3H, *J*=7.5, $NC(O)CH_2CH_3$), 1.87-1.93 (m, 2H, CH_2^{pip}), 2.02-2.09 (m, 4H, CH_2^{pip}), 2.36 (q, 2H, *J*=7.6, $NC(O)CH_2CH_3$), 2.82-2.87 (m, 2H, CH_2^{pip}), 3.21 (s, 3H, CH_3), 3.76 (s, 2H, CH_2), 4.31-4.35 (m, 1H, CH^{pip}), 6.61 (s, 1H, CH^{furan}), 6.88 (d, 2H, *J*=8.8, H^{Ar}), 7.05 (d, 2H, *J*=8.8, H^{Ar}), 7.19-7.28 (m, 2H, H^{Ar}), 7.46-7.74 (m, 2H, H^{Ar});**^{13}C-NMR (150 MHz, $CDCl_3$):** δ =9.24 i 9.94 ($NCOCH_2CH_3$), 28.93 ($NCOCH_2CH_3$), 30.57 (C^{pip}), 37.67 (CH_3), 50.15 (C^{pip}), 55.24 (C^{pip}), 55.28 (CH_2), 74.60 (C^{pip}), 106.23 (C^{furan}), 111.51, 116.99, 120.99, 122.95, 124.47, 128.47, 137.33, 154.31 (C^{Ar}), 170.49 (C=O)

N-(4-(((1-Benzylpiperidin-4-yl)oxy)methyl)benzyl)-N-methylpropionamide (**12c.1**):
(81%): Rf = 0.44 (Hexane/EtOAc 1:5); **^{1}H-NMR ($CDCl_3$, 600MHz):**δ =1.35-1.60 (m, 3H, $C(O)CH_2\underline{CH_3}$), 1.68-1.74 (m, 2H, CH_2^{pip}), 1.90-1.96 (m, 2H, CH_2^{pip}), 2.24-2.29 (m, 2H, CH_2^{pip}), 2.36-2.41 (m, 2H, $C(O)\underline{CH_2}CH_3$), 2.76-2.81 (m, 2H, CH_2^{pip}), 2.89 (s, 1.7H, CH_3), 2.94 (s, 1.3H, CH_3), 3.42-3.47 (m, 1H, CH^{pip}), 3.56 (s, 2H, $CH_2C_6H_5$), 4.49 (s, 1H, $\underline{CH_2}N$), 4.51 (s, 2H, $O\underline{CH_2}C_6H_5$), 4.57 (s, 1H, $\underline{CH_2}NH_2$), 7.24-7.33 (m, 9H, HAr); **^{13}C-NMR (150 MHz, $CDCl_3$):** δ = 9.55 ($C(O)CH_2\underline{C}H_3$), 26.53 i 26.98 ($C(O)\underline{C}H_2CH_3$), 28.37 i 30.93 (C^{pip}), 34.17 i 34.85 (CH_3), 50.83 ($\underline{C}H_2N$ i C^{pip}), 62.73 i 62.76 ($\underline{C}H_2C_6H_5$), 69.58 i 69.70 (O $\underline{C}H_2C_6H_4$), 74.69 (C^{pip}), 126.55, 127.49, 127.95, 128.25, 128.32, 128.47, 129.66, 129.68 (C^{Ar})

N-(4-(((1-(Benzofuran-2-ylmethyl)piperidin-4-yl)oxy)methyl)benzyl)-N-methylpropionamide (**12c.2**):
(99%): Rf = 0.92 (CH_2Cl_2/MeOH/$NH_{3(aq)}$ 8:1:1%); **^{1}H-NMR ($CDCl_3$, 600MHz):**δ =1.13-1.20 (m, 3H, $NCOCH_2CH_3$), 1.70-1.78 (m, 2H, CH_2^{pip}), 1.90-1.96 (m, 2H, CH_2^{pip}), 2.26-2.33 (m,

2H, CH_2^{pip}), 2.37 (q, 2H, *J*=7.4, NCOC*H*$_2$CH$_3$), 2.79-2.85 (m, 2H, CH_2^{pip}), 2.88 (s, 1.7H, CH_3), 2.93 (s, 1.3H, CH_3), 3.39-3.45 (m, 1H, CH^{pip}), 3.68 (s, 2H, C*H*$_2$NC$_5$H$_9$), 4.48 (s, 2H, OC*H*$_2$C$_6$H$_4$), 4.50 (s, 0.8H, C$_6$H$_4$C*H*$_2$N) , 4.59 (s, 1.2H, C$_6$H$_4$C*H*$_2$N), 6.56 (s, 1H, CH^{furan}), 7.07-7.32 (m, 6H, CH^{Ar}), 7.43-7.53 (m, 2H, CH^{Ar}); **^{13}C-NMR (150 MHz, CDCl$_3$):** δ =9.53 (NCOCH$_2$*C*H$_3$), 26.50 (NCO*C*H$_2$CH$_3$), 31.28 (CH_2^{pip}), 34.82 (CH_3), 50.78 (CH_2^{pip}), 55.60 (C$_6$H$_4$*C*H$_2$N), 58.23 (*C*H$_2$NC$_5$H$_9$), 69.62 (C$_6$H$_4$*C*H$_2$N), 74.20 (CH^{pip}), 105.69 (C^{furan}), 111.47, 120.85, 122.80, 124.04, 126.50, 127.92, 128.51, 137.00, 138.21, 155.26 (C^{Ar}), 174.02 (CO)

N-(3-(((1-Benzylpiperidin-4-yl)oxy)methyl)benzyl)-N-methylpropionamide (**12b.1**):
(99%): Rf = 0.96 (CH_2Cl_2/MeOH/$NH_{3(aq)}$ 8:1:1%); **^{1}H-NMR (CDCl$_3$, 600MHz):**δ =1.14 (t, 3H, *J*=7.4, C*H*$_3$CH$_2$CO), 1.64-1.71 (m, 2H, CH_2^{pip}), 1.88-1.94 (m, 2H, CH_2^{pip}), 2.10-2.17 (m, 2H, CH_2^{pip}), 2.39 (q, 2H, *J*=7.4, (CH$_3$C*H*$_2$CO), 2.72-2.79 (m, 2H, CH_2^{pip}), 2.90 (s, 2H, CH_3), 2.94 (s, 1H, CH_3), 3.38-3.45 (m, 1H, CH^{pip}), 3.49 (s, 2H, C$_6$H$_5$C*H*$_2$), 4.51 (s, 2H, -OC*H*$_2$C$_6$H$_4$), 4.52 (s, 1H, C*H*$_2$N), 4.59 (s, 1H, C*H*$_2$N), 7.12-7.34 (m, 9H, CH^{Ar}); **^{13}C-NMR (150 MHz, CDCl$_3$):** δ =9.74 (*C*H$_3$CH$_2$CO), 26.43(CH$_3$*C*H$_2$CO), 31.48 (2xC^{pip}), 34.81 (CH_3), 50.84 (2xC^{pip}), 51.61 (*C*H$_2$N), 63.11 (C$_6$H$_5$*C*H$_2$), 69.61 and 69.70 (, O*C*H$_2$C$_6$H$_4$ and C^{pip}), 125.40, 126.57, 127.06, 127.19, 128.29, 129.21, 138.66, 140.03 (C^{Ar}), 174.34 (CO)

N-(3-(((1-(Benzofuran-2-ylmethyl)piperidin-4-yl)oxy)methyl)benzyl)-N-methylpropionamide (**12b.2**):
(99%): Rf = 0.16 (Hexane/EtOAc 5:1); **^{1}H-NMR (CDCl$_3$, 600MHz):**δ =1.19 (t, 3H, *J*=7.4, C*H*$_3$CH$_2$CO), 1.70-1.77 (m, 2H, CH_2^{pip}), 1.91-1.96 (m, 2H, CH_2^{pip}), 2.25-2.33 (m, 2H, CH_2^{pip}), 2.39 (q, 2H, *J*=7.4, (CH$_3$C*H*$_2$CO), 2.79-2.87 (m, 2H, CH_2^{pip}), 2.89 (s, 2H, CH_3), 2.94 (s, 1H, CH_3), 3.38-3.47 (m, 1H, CH^{pip}), 3.69 (s, 2H, C$_8$H$_5$C*H*$_2$), 4.50 (s, 3H, - C*H*$_2$N , OC*H*$_2$C$_6$H$_4$), 4.58 (s, 1H, C*H*$_2$N), 6.58 (s, 1H, CH^{furan}), 7.11-7.33 (m, 6H, CH^{Ar}), 7.45-7.54 (m, 2H, CH^{Ar}); **^{13}C-NMR (CDCl$_3$, 150MHz):** δ =9.77 (*C*H$_3$CH$_2$CO), 26.46(CH$_3$*C*H$_2$CO), 31.23 (2xC^{pip}), 34.84 (CH_3), 51.07 (2xC^{pip}), 53.34 (*C*H$_2$N), 55.52 (C$_8$H$_5$*C*H$_2$), 69.75 (O*C*H$_2$C$_6$H$_4$), 77.23 (C^{pip}), 105.74 (C^{furan}), 111.45, 120.83, 124.02, 126.60, 128.78, 137.84, 139.46, 155.21 (C^{Ar}), 174.03 (CO)

N-(2-(((1-Benzylpiperidin-4-yl)oxy)methyl)benzyl)-N-methylpropionamide (**12a.1**):
(98%): Rf = 0.68 (CH_2Cl_2/MeOH/$NH_{3(aq)}$ 8:1:1%); **^{1}H-NMR (CDCl$_3$, 600MHz):** δ =1.14(t, 3H, *J*=7.4, COCH$_2$C*H*$_3$), 1.64-1.73 (m, 2H, CH_2^{pip}), 1.89-1.95 (m, 2H, CH_2^{pip}), 2.18-2.26 (m, 2H, CH_2^{pip}), 2.31 (m, 2H, COC*H*$_2$CH$_3$), 2.73-2.82 (m, 2H, CH_2^{pip}), 2.89 (s, 1.8H, CH_3), 2.98

(s, 1.2H, CH_3), 3.41-3.47 (m, 1H, CH^{pip}), 3.54 (s, 2H, CH_2-C_6H_5), 4.49 (s, 2H, $OCH_2C_6H_4$), 4.63 (s, 1H, CH_2N), 4.70 (s, 1H, CH_2N), 7.06-7.14 (m, 1H, H^{Ar}), 7.22-7.36 (m, 8H, H^{Ar})*;* **^{13}C-NMR ($CDCl_3$, 150MHz):** δ =8.53 ($\mathit{C}H_3CH_2CO$), 28.86 ($CH_3\mathit{C}H_2CO$), 31.02 (C^{pip} 2x), 34.55 (CH_3), 47.66 (C^{pip} 2x), 50.47 (CH_2N), 62.73 ($CH_2C_6H_5$), 67.88 and 68.44 ($OCH_2C_6H_5$, C^{pip}), 125.35, 127.38, 128.06, 128.20, 128.38, 128.83, 129.55, 129.89, 135.59, 135.86, 136.85, 137.74 (C^{Ar}), 174.81 (CO)

N-(2-(((1-(Benzofuran-2-ylmethyl)piperidin-4-yl)oxy)methyl)benzyl)-N-methylpropionamide (**12a.2**): (98%): Rf = 0.62 (CH_2Cl_2/MeOH/$NH_{3(aq)}$ 8:1:1%); **^{1}H-NMR ($CDCl_3$, 600MHz):** δ =1.18 (t, 3H, J=7.4, $COCH_2C\mathit{H_3}$), 1.68-1.77 (m, 2H, CH_2^{pip}), 1.91-1.97 (m, 2H, CH_2^{pip}), 2.24-2.34 (m, 2H, CH_2^{pip}), 2.38-2.44 (m, 2H, $COC\mathit{H_2}CH_3$), 2.80-2.85 (m, 2H, CH_2^{pip}), 2.87 (s, 1.6H, CH_3), 2.93 (s, 1.4H, CH_3), 3.39-3.46 (m, 1H, CH^{pip}), 3.69 (s, 2H, $CH_2C_8H_5O$), 4.51 (s, 2H, $OCH_2C_6H_4$), 4.64 (s, 1H, CH_2N), 4.69 (s, 1H, CH_2N), 6.58 (s, 1H, H^{furan}), 7.06-7. 36 (m, 6H, H^{Ar}), 7.43-7. 53 (m, 2H, H^{Ar}); **^{13}C-NMR ($CDCl_3$, 150MHz):**δ =8.46 ($\mathit{C}H_3CH_2CO$), 26.87 ($CH_3\mathit{C}H_2CO$), 31.13 (C^{pip} 2x), 34.77 (CH_3), 47.61 (C^{pip} 2x), 53.58 (CH_2N), 55.37 ($OCH_2C_6H_4$), 67.80 ($OCH_2C_6H_4$), 74.35(C^{pip}), 105.76 (C^{furan}), 111.35, 120.78, 122.75, 123.96, 125.27, 128.13, 129.83, 136.78, 154.73 (C^{Ar}), 173.98 (CO)

General procedure for the preparation of compounds 8a.1, 8b.1, 8c.1, 8a.2, 8b.2, 8c.2, 14, 15, 21

A solution of the appropriate alcohol (1 equiv.) in 80 mL of dry toluene sodium hydride (2 equiv.) was added. After stirring at room temperature for 1 h, 15-crown-5 ether (0.1 equiv.) was added dropwise to the suspension followed by appropriate bromide or chloride (1.2 equiv.) (in the case of the synthesis compound **21**, 15 minutes after the addition of 2,6-bis(bromomethyl)naphthalene, a small amount of DCM was added). The reaction mixture was stirred at room temperature for 48 h. An excess of sodium hydride was quenched by dropwise addition of ethanol (10 mL). The solvent was evaporated. Then the mixture was washed with 100 mL of water. The water phase was washed three times with 50 mL of dichloromethane, then the combined organic phases were dried over Na_2SO_4.
The solvent was removed under vacuum, and the crude product was purified by column chromatography to obtain the pure products, i.e. compounds **8a.1, 8b.1, 8c.1, 8a.2, 8b.2, 8c.2, 14, 15, 21**, as sticky oils.

4-(((1-Benzylpiperidin-4-yl)oxy)methyl)benzonitrile (**8c.1**):

(15%): Rf = 0.70 (CH_2Cl_2/MeOH/$NH_{3(aq)}$ 8:1:1%); **^{1}H-NMR ($CDCl_3$, 600MHz):**δ = 1.66-1.71 (m, 2H, CH_2^{pip}), 1.88-1.93 (m, 2H, CH_2^{pip}), 2.13-2.18 (m, 2H, CH_2^{pip}), 2.71-2.77 (m, 2H, CH_2^{pip}), 3.40-3.44 (m, 1H, CH^{pip}), 3.49 (s, 2H, $CH_2C_6H_5$), 4.75 (s, 2H, OCH_2), 7.23-7.26 (m, 1 H, HAr), 7.30 (d, 4H, J=4.3, HAr), 7.44 (d, 2H, J=8.16, HAr), 7.61 (d, 2H, J=8.22, HAr); **^{13}C-NMR (150 MHz, $CDCl_3$):** δ =31.53 (C^{pip}), 51.19 (C^{pip}), 63.19 ($\underline{C}H_2C_6H_5$), 69.00 (O $\underline{C}H_2C_6H_4$), 77.02 (C^{pip}), 111.32 (C^{Ar}), 119.09 (CN), 127.18, 127.75, 128.39, 129.25, 132.36, 138.77, 144.98 (C^{Ar})

4-(((1-(Benzofuran-2-ylmethyl)piperidin-4-yl)oxy)methyl)benzonitrile (**8c.2**):

(82%): Rf = 0.77 (CH_2Cl_2/MeOH/$NH_{3(aq)}$ 8:1:1%); **^{1}H-NMR ($CDCl_3$, 600MHz):**δ =1.72-1.78 (m, 2H, CH_2^{pip}), 1.91-1.96 (m, 2H, CH_2^{pip}), 2.28-2.33 (m, 2H, CH_2^{pip}), 2.79-2.86 (m, 2H, CH_2^{pip}), 3.41-3.46 (m, 1H, CH^{pip}), 3.68 (s, 2H, $CH_2NC_5H_9$), 4.56 (s, 2H, $OCH_2C_6H_4$), 6.57 (s, 1H, CH^{furan}), 7.18-7.26 (m, 3H, CH^{Ar}), 7.41-7.61 (m, 5H, CH^{Ar}); **^{13}C-NMR (150 MHz, $CDCl_3$):** δ =31.27 (2xCH_2^{pip}), 50.97 (2xCH_2^{pip}), 55.60 ($CH_2NC_5H_9$), 69.00 ($OCH_2C_6H_5$), 70.86 (CH^{pip}), 105.73 (C^{furan}), 119.07 (CN), 120.88, 122.86, 124.11, 127.73, 128.50, 132.36, 144.85, 155.28 (C^{Ar})

3-(((1-Benzylpiperidin-4-yl)oxy)methyl)benzonitrile (**8b.1**):

(43%): Rf = 0.65 (CH_2Cl_2/MeOH/$NH_{3(aq)}$ 8:1:1%); **^{1}H-NMR ($CDCl_3$, 600MHz):**δ = 1.66-1.72 (m, 2H, CH_2^{pip}), 1.88-1.94 (m, 2H, CH_2^{pip}), 2.13-2.20 (m, 2H, CH_2^{pip}), 2.71-2.77 (m, 2H, CH_2^{pip}), 3.40-3.45 (m, 1H, CH^{pip}), 3.49 (s, 2H, $C_6H_5CH_2$), 4,54 (s, 2H, $OCH_2C_6H_4$), 7.22-7.52 (m, 9H, CH^{Ar}); **^{13}C-NMR (150 MHz, $CDCl_3$):** δ =31.46 (2xC^{pip}), 51.16 (2xC^{pip}), 63.15 ($C_6H_5CH_2$-), 68.69 ($OCH_2C_6H_4$), 75.56 (C^{pip}), 112.63 (C^{Ar}), 119.04 (CN), 127.15, 128.37, 129.28, 130.87, 131.18, 131.65, 138.70, 140.93 (C^{Ar})

3-(((1-(Benzofuran-2-ylmethyl)piperidin-4-yl)oxy)methyl)benzonitrile (**8b.2**):

(73%): Rf = 0.92 (CH_2Cl_2/MeOH/$NH_{3(aq)}$ 8:1:1%); **^{1}H-NMR ($CDCl_3$, 600MHz):**δ =1.72-1.79 (m, 2H, CH_2^{pip}), 1.92-1.98 (m, 2H, CH_2^{pip}), 2.26-2.35 (m, 2H, CH_2^{pip}), 2.77-2.85 (m, 2H, CH_2^{pip}), 3.40-3.47 (m, 1H, CH^{pip}), 3.69 (s, 2H, $CH_2NC_5H_9$), 4.54 (s, 2H, $OCH_2C_6H_4$)
6.58 (s, 1H, CH^{furan}), 7.19-7.27 (m, 3H, CH^{Ar}), 7.41-7.67 (m, 5H, CH^{Ar}); **^{13}C-NMR (150 MHz, $CDCl_3$):** δ =31.24 (2xC^{pip}), 51.09 (2xC^{pip}), 55.64 ($CH_2NC_5H_9$), 68.72 ($OCH_2C_6H_5$), 74.94 (C^{pip}), 105.78 (C^{furan}), 119.08 (CN), 120.89, 122.87, 124.11, 129.32, 130.91, 131.27, 140.81, 155.28 (C^{Ar})

2-(((1-Benzylpiperidin-4-yl)oxy)methyl)benzonitrile (**8a.1**): (86%): Rf = 0.70 (CH_2Cl_2/MeOH/$NH_{3(aq)}$ 8:1:1%); **^{1}H-NMR ($CDCl_3$, 600MHz):**δ =1.66-1.74 (m, 2H, CH_2^{pip}), 1.91-1.99 (m, 2H, CH_2^{pip}), 2.12-2.19 (m, 2H, CH_2^{pip}), 2.70-2.78 (m, 2H, CH_2^{pip}), 3.40-3.53 (m, 3H, CH^{pip}, $CH_2C_6H_5$), 4.67 (s, 2H, $OCH_2C_6H_4$), 7.19-7.35 (m, 6H, H^{Ar}), 7.49-7.60 (m,3H, H^{Ar}); **^{13}C-NMR ($CDCl_3$, 150MHz):**δ =31.17 (C^{pip} 2x), 50.95 (C^{pip} 2x), 62.93 ($CH_2C_6H_5$), 67.55($OCH_2C_6H_5$), 75.75 (C^{pip}), 111.20 (Cq), 117.35 (CN), 126.93, 128.16, 128.52, 129.02, 132.82, 138.46, 142.66 (C^{Ar})

2-(((1-(Benzofuran-2-ylmethyl)piperidin-4-yl)oxy)methyl)benzonitrile (**8b.2**): (75%): Rf = 0.93 (CH_2Cl_2/MeOH/$NH_{3(aq)}$ 8:1:1%); **^{1}H-NMR ($CDCl_3$, 600MHz):**δ =1.73-1.82 (m, 2H, CH_2^{pip}), 1.95-2.02 (m, 2H, CH_2^{pip}), 2.24-2.36 (m, 2H, CH_2^{pip}), 2.75-2.90 (m, 2H, CH_2^{pip}), 3.48-3.58 (m, 1H, CH^{pip}), 3.69 (s, 2H, $CH_2C_8H_5O$), 4.70 (s, 2H, $OCH_2C_6H_4$), 6.58 (s, 1H, H^{furan}), 7.19-7.60 (m,8H, H^{Ar}); **^{13}C-NMR ($CDCl_3$, 150MHz):** δ =31.15 (C^{pip} 2x), 51.03 (C^{pip} 2x), 55.61 ($CH_2C_8H_5O$), 67.76 ($OCH_2C_6H_4$), 75.52 (^{Cpip}), 105.73 (C^{furan}), 117.55 (CN), 120.84, 122.79, 124.04, 128.08, 128.46, 132.87, 142.78, 154.95(C^{Ar})

1-Benzyl-4-((4'-(chloromethyl)-[1,1'-biphenyl]-4-yl)methoxy)piperidine (**14**):
(17%): Rf = 0.78 (CH_2Cl_2/MeOH/$NH_{3(aq)}$ 8:1:1%); **^{1}H-NMR ($CDCl_3$, 600MHz):** δ =1.65-1.75 (m, 2H, CH_2^{pip}), 1.89-1.97 (m, 2H, CH_2^{pip}), 2.12-2.19 (m, 2H, CH_2^{pip}), 2.70-2.80 (m, 2H, CH_2^{pip}), 3.42-3.47 (m, 1H, CH^{pip}), 3.50 (s, 2H, $CH_2C_6H_5$), 4.54 (s, 0.7H, OCH_2), 4.57 (s, 2H, CH_2Cl), 4.64 (s, 1.3H, OCH_2), 7.22-7.59 (m, 13H, H^{Ar}); **^{13}C-NMR (150MHz, $CDCl_3$):** δ =31.54 (C^{pip}), 46.27 (CH_2Cl), 51.34 (C^{pip}), 63.23 (*C*$H_2C_6H_5$), 72.66 (OCH_2), 74.86 (C^{pip}), 127.32, 127.63, 128.19, 128.38, 129.27, 136.61, 137.81, 138.62, 138.75, 139.84, 140.34, 141.36 (C^{Ar})

Benzofuran-2-yl(4-((4'-(chloromethyl)-[1,1'-biphenyl]-4-yl)methoxy)piperidin-1-yl)methanone (**15**):
(40%): Rf = 0.44 (Hexane- EtOAc 1:1); **^{1}H-NMR ($CDCl_3$, 600MHz):** δ = 1.77-1.86 (m, 2H, CH_2^{pip}), 1.94-2.03 (m, 2H, CH_2^{pip}), 3.58-3.69 (m, 2H, CH_2^{pip}), 3.74-3.80 (m, 1H, CH^{pip}), 4.02-4.13 (m, 2H, CH_2^{pip}), 4.63 (s, 4H, OCH_2 and CH_2Cl), 7.23-7.67 (m, 13H, H^{Ar}); **^{13}C-NMR (150MHz, $CDCl_3$):** δ =21.23 (CH_2^{pip}), 46.21 (CH_2^{pip}), 60.57 (CH_2Cl), 69.93 (OCH_2), 73.52 (CH^{pip}), 112.05 (CH^{furan}), 122.36, 123.72, 127.61, 128.14, 128.36, 129.28, 136.71, 138.09, 140.08, 141.20, 149.39, 154.73, 160.06 (C^{Ar}), 171.33 (CO)

tert-Butyl 4-((6-(bromomethyl)naphthalen-2-yl)methoxy)piperidine-1-carboxylate (**21**): (13%): Rf = 0.83 (CH_2Cl_2/MeOH 8:1); **^{1}H-NMR (600MHz, $CDCl_3$):** δ =1.45 (s, 9H, 3xCH_3), 1.57-1.65 (m, 2H, CH_2^{pip}), 1.84-1.91 (m, 2H, CH_2^{pip}), 3.06-3.13 (m, 2H, CH_2^{pip}), 3.56-3.62 (m, 1H, CH^{pip}), 3.75-3.86 (m, 2H, CH_2^{pip}), 4.66 (s, 2H, CH_2), 4.71 (s, 2H, CH_2), 7.49 (ddd, 2H, *J*=12.6 Hz, *J*=8.4 Hz, *J*=1.2 Hz, H^{Ar}), 7.76-7.84 (m, 4H, H^{Ar}); **^{13}C-NMR ($CDCl_3$, 150 MHz):** δ =28.66 (3xCH_3), 31.30 (CH_2^{pip}), 34.27 (CH_2Br), 46.85 (CH_2^{pip}), 70.06 (OCH_2), 74.35 (CH^{pip}), 79.69 ($\underline{C}(CH_3)_3$), 126.09 and 126.44, 127.28 and 127.92, 128.45 and 128.92, 132.88, 135.29, 137.37 (C^{Ar}), 155.06 (CO)

Synthesis of compounds 16

To a solution of corresponding chloride (**15**) (1 equiv.) and K_2CO_3 (2 equiv.) in 20.0 mL of acetonitrile, *N*-methyl-*N*-propylamine was added (2 equiv.). The mixture was stirred at room temperature for 12 h. Water (20 mL) was then added, and the reaction mixture was extracted with dichloromethane (3x 20 mL). The organic layer was dried over Na_2SO_4, the solvent was removed under vacuum, and the crude product was purified by column chromatography to give compound **16,** as a sticky oil.

Benzofuran-2-yl(4-((4'-((methyl(propyl)amino)methyl)-[1,1'-biphenyl]-4-yl)methoxy)piperidin-1-yl)methanone (**16**): (49%): Rf = 0.50 (CH_2Cl_2/MeOH/$NH_{3(aq)}$ 8:1:1%); **^{1}H-NMR ($CDCl_3$, 600MHz):** δ = 0.92 (t, 3H, *J*=7.32, $CH_3CH_2CH_2N$), 1.51-1.59 (m, 2H, $CH_3CH_2CH_2N$), 1.77-1.87 (m, 2H, CH_2^{pip}), 1.94-2.03 (m, 2H, CH_2^{pip}), 2.22 (s, 3H, CH_3), 2.37 (t, 2H, *J*=7.44, $CH_3CH_2CH_2N$), 3.53 (s, 2H, $CH_2C_{12}H_8$), 3.58-3.69 (m, 2H, CH_2^{pip}), 3.74-3.79 (m, 1H, CH^{pip}), 4.02-4.12 (m, 2H, CH_2^{pip}), 4.69 (s, 2H, OCH_2), 7.19-7.68 (m, 13H, H^{Ar}); **^{13}C-NMR (150MHz, $CDCl_3$):** δ =12.06 ($CH_3CH_2CH_2N$), 20.69 ($CH_3CH_2CH_2N$), 42.40 (CH_2^{pip}), 59.67 (CH_3, CH_2^{pip}), 62.10 ($CH_3CH_2CH_2N$), 69.99 ($CH_2N(CH_3)C_3H_7$), 73.40 (OCH_2, CH^{pip}), 112.05 (CH^{furan}), 122.35, 123.71, 126.49, 127.22, 128.12, 129.67, 137.56, 139.60, 140.64, 149.38, 154.74, 160.05 (CAr), 171.33 (CO)

Synthesis of compound 18

To a suspension of naphthalene-2,6-dicarboxylic acid (**17**) (1 equiv.) and Li_2CO_3 (6 equiv.) in dichloromethane, CH_3I (6 equiv.) was added. The reaction mixture was stirred at room

temperature overnight. After this time 1N HCl (20 mL) was carefully added and the precipitated product as a white solid was filtered, washed with cold water, dried, and taken to the next step without further purification.

Dimethyl naphthalene-2,6-dicarboxylate (**18**):
(46%); **^{1}H-NMR (600MHz, $CDCl_3$):** δ =4.00 (s, 6H, 2xCH_3), 7.99 (d, 2H, *J*=8.4 Hz, H^{Ar}), 8.12 (d, 2H, *J*=8.4 Hz, H^{Ar}), 8.62-8.64 (m, 2H, H^{Ar})

Synthesis of compound 19

To the solution of dimethyl naphthalene-2,6-dicarboxylate **18** (1 equiv.) in dry THF, $LiAlH_4$ (2.6 equiv.) was added portion-wise for 30 min at room temperature, and the resulting reaction mixture was stirred under argon for 24 h. After this time 5mL of methanol was added drop-wise. The suspension was filtered through the pad of Celite and washed several times with THF. The solvent was removed under vacuum and the residue was extracted with acetone. Evaporation of acetone gave a crude product as a white solid which was taken to the next step without further purification.

Naphthalene-2,6-diyldimethanol (**19**):
(99%): Rf = 0.44 (Hexane/EtOAc 3:1); **^{1}H-NMR (600MHz, $CDCl_3$):** δ =4.64 (d, 2H, *J*=6.0 Hz, 2xCH_2), 5.36 (t, 2H, *J*=6.0 Hz, 2xOH), 7.43 (d, 2H, *J*=8.4 Hz, H^{Ar}), 7.71-7.79 (m, 2H, H^{Ar}), 7.83 (d, 2H, *J*=8.4 Hz, H^{Ar})

Synthesis of compound 20

To a solution of 2,6-bis(hydroxymethyl)naphthalene **19** (1 equiv.) in dichloromethane (5,5 mL) and DMF (4.8mL), phosphorus tribromide (1 equiv.) was added drop-wise under argon. The reaction mixture was stirred for one hour at room temperature. After this time, the reaction mixture was poured into a saturated ice-cold $NaHCO_3$ $_{(aq)}$ solution, extracted with dichloromethane (3x 25mL), dried over $MgSO_4$, and filtered. The solvent was evaporated to afford yellowish-white powder. The crude product was taken to the next step without further purification.

2,6-Bis(bromomethyl)naphthalene (**20**):

(83%): Rf = 0.48 (Hexane/EtOAc 3:1); **^{1}H-NMR (600MHz, $CDCl_3$):** δ =4.66 (s, 4H, 2xCH_2), 7.52 (dd, 2H, J=8.4 Hz, J=1.8 Hz, H^{Ar}), 7.79-7.82 (m, 4H, H^{Ar})

Synthesis of compound 22

To the stirred solution of *tert*-butyl 4-((6-(bromomethyl)naphthalen-2-yl)methoxy)piperidine-1-carboxylate **21** (1 equiv.) in dry acetone K_2CO_3 (2 equiv.) was added. Next *N*-methylpropylamine (2 equiv.) was added dropwise. The resulting mixture was stirred at room temperature overnight. After completion of the reaction, water was added (5mL). The mixture was extracted with dichloromethane (3x20mL). The organic layers were dried over Na_2SO_4 and filtered. The solvent was evaporated and the residue was purified by silica gel-flash column chromatography (eluent: CH_2Cl_2:MeOH gradient 40:1 to 10:1) to give compound **22** as a sticky oil.

tert-Butyl-4-((6-((methyl(propyl)amino)methyl)naphthalen-2-yl)methoxy)piperidine-1-carboxylate (**22**):
(86%): Rf = 0.47 (CH_2Cl_2/MeOH 8:1); **^{1}H-NMR (600MHz, $CDCl_3$):** δ =0.91 (t, 3H, J=7.2Hz, $C\underline{H_3}CH_2CH_2N$), 1.46 (s, 9H, 3x$CH_3$), 1.53-1.63 (m, 4H, CH_2^{pip}, $CH_3C\underline{H_2}CH_2N$), 1.85-1.92 (m, 2H, CH_2^{pip}), 2.23 (s, 3H, CH_3), 2.38 (t, 3H, J=7.2Hz, $CH_3CH_2C\underline{H_2}N$), 3.06-3.12 (m, 2H, CH_2^{pip}), 3.57-3.62 (m, 1H, CH^{pip}), 3.63 (s, 2H, CH_2), 3.75-3.85 (m, 2H, CH_2^{pip}), 4.70 (s, 2H, CH_2), 7.45 (dd, 1H, J=8.4 Hz, J=1.2 Hz, H^{Ar}), 7.49 (dd, 1H, J=8.4 Hz, J=1.2 Hz, H^{Ar}), 7.71-7.73 (m, 1H, H^{Ar}), 7.75-7.80 (m, 3H, H^{Ar}); **^{13}C-NMR ($CDCl_3$, 150 MHz):** δ = 12.09 ($\underline{C}H_3CH_2CH_2N$), 20.73 ($CH_3\underline{C}H_2CH_2N$), 28.65 (3x$CH_3$), 31.27 ($CH_2^{pip}$), 42.58 ($CH_3$), 53.66 ($CH_2^{pip}$), 59.81 ($CH_3CH_2\underline{C}H_2N$), 62.64 ($CH_2$), 70.18 ($OCH_2$), 74.15 ($CH^{pip}$), 79.65 ($\underline{C}(CH_3)_3$), 125.92, 126.16, 127.51, 127.92, 127.98, 128.21, 132.76, 133.07, 136.03 (C^{Ar}), 155.06 (CO)

Synthesis of compound 23

To a solution of *tert*-butyl 4-((6-((methyl(propyl)amino)methyl)naphthalen-2-yl)methoxy)piperidine-1-carboxylate **22** (1 equiv.) in dry chloroform (0.3 mL), 4N solution of HCl in dioxane (10 equiv.) was added and the resulting reaction mixture was stirred at room temperature overnight. After this time, the solvent was evaporated and the residue was

triturated with ethyl acetate to give the crude product which was taken to the next step without further purification.

N-Methyl-N-((6-((piperidin-4-yloxy)methyl)naphthalen-2-yl)methyl)propan-1-amine hydrochloride (**23**):

(86%): Rf = 0.06 (CH_2Cl_2/MeOH 8:1); **^{1}H-NMR (600MHz, CD_3OD):** δ =1.03 (t, 3H, *J*=7.8Hz, $C\underline{H_3}CH_2CH_2N$), 1.67-1.83 (m, 2H, $CH_3C\underline{H_2}CH_2N$), 1.83-1.92 (m, 2H, CH_2^{pip}), 1.97-2.05 (m, 2H, CH_2^{pip}), 2.73 (s, 3H, CH_3), 2.96-3.15 (m, 4H, CH_2^{pip}, $CH_3CH_2C\underline{H_2}N$), 3.24-3.30 (m, 2H, CH_2^{pip}), 3.72-3.78 (m, 1H, CH^{pip}), 4.30-4.34 (m, 1H, NCH_2), 4.49-4.53 (s, 1H, NCH_2), 4.67 (s, 2H, OCH_2), 7.58 (ddd, 2H, *J*=25.2 Hz, *J*=8.4 Hz, *J*=0.6 Hz, H^{Ar}), 7.81-7.91 (m, 3H, H^{Ar}), 7.97-7.99 (m, 1H, H^{Ar}); **^{13}C-NMR (CD_3OD, 150 MHz):** δ =11.18 ($\underline{C}H_3CH_2CH_2N$), 18.73 ($CH_3\underline{C}H_2CH_2N$), 28.67 ($CH_2^{pip}$), 40.13 ($CH_2^{pip}$), 42.04 ($CH_3$),58.66 ($CH_3CH_2\underline{C}H_2N$), 60.96 ($NCH_2$), 71.17 ($OCH_2$), 71.41 ($CH^{pip}$), 127.12, 127.89, 128.32, 128.80, 130.24, 135.03, (C^{Ar})

2. NMR spectra

SpinWorks 4: ADS-021

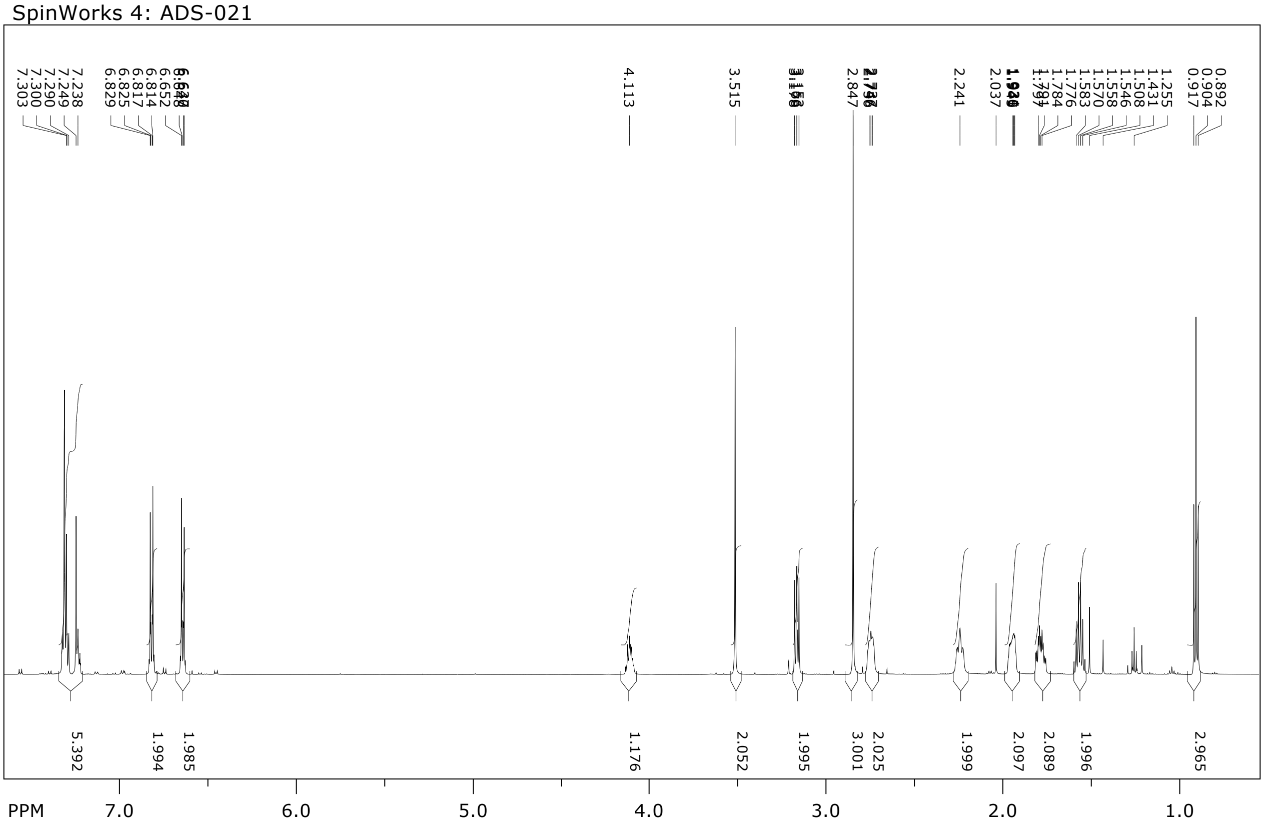

file: F:\FID UMED\bo-116-1\10\fid expt: <zg30>
transmitter freq.: 600.263707 MHz
time domain size: 65536 points
width: 12335.53 Hz = 20.5502 ppm = 0.188225 Hz/pt
number of scans: 16

freq. of 0 ppm: 600.260025 MHz
processed size: 32768 complex points
LB: 0.300 GF: 0.0000

Figure S1.^{1}H NMR ($CDCl_3$, 600MHz) spectra of compound ADS021 base.

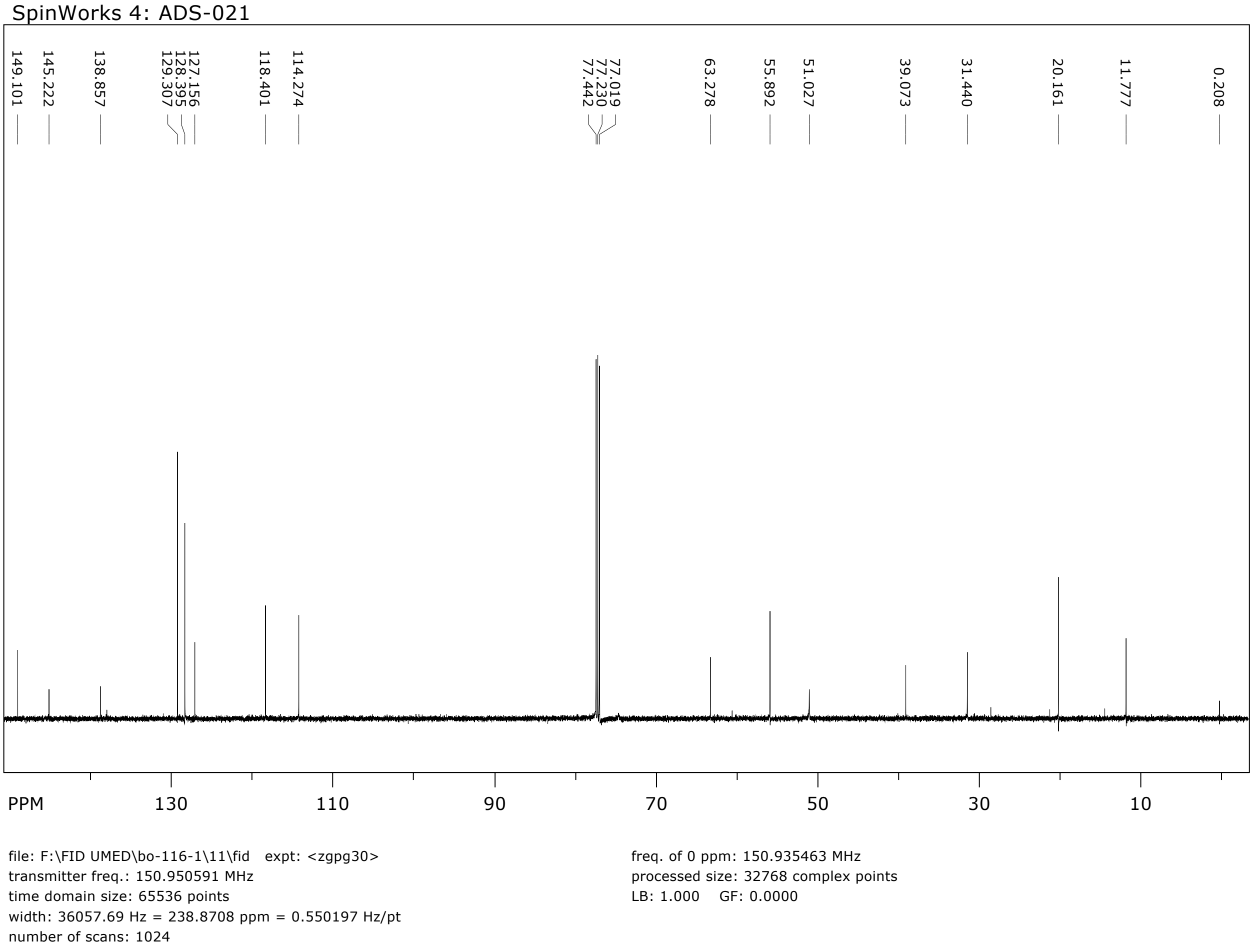

Figure S2. ^{13}C NMR (CDCl$_3$, 150 MHz) spectra of compound ADS021 base.

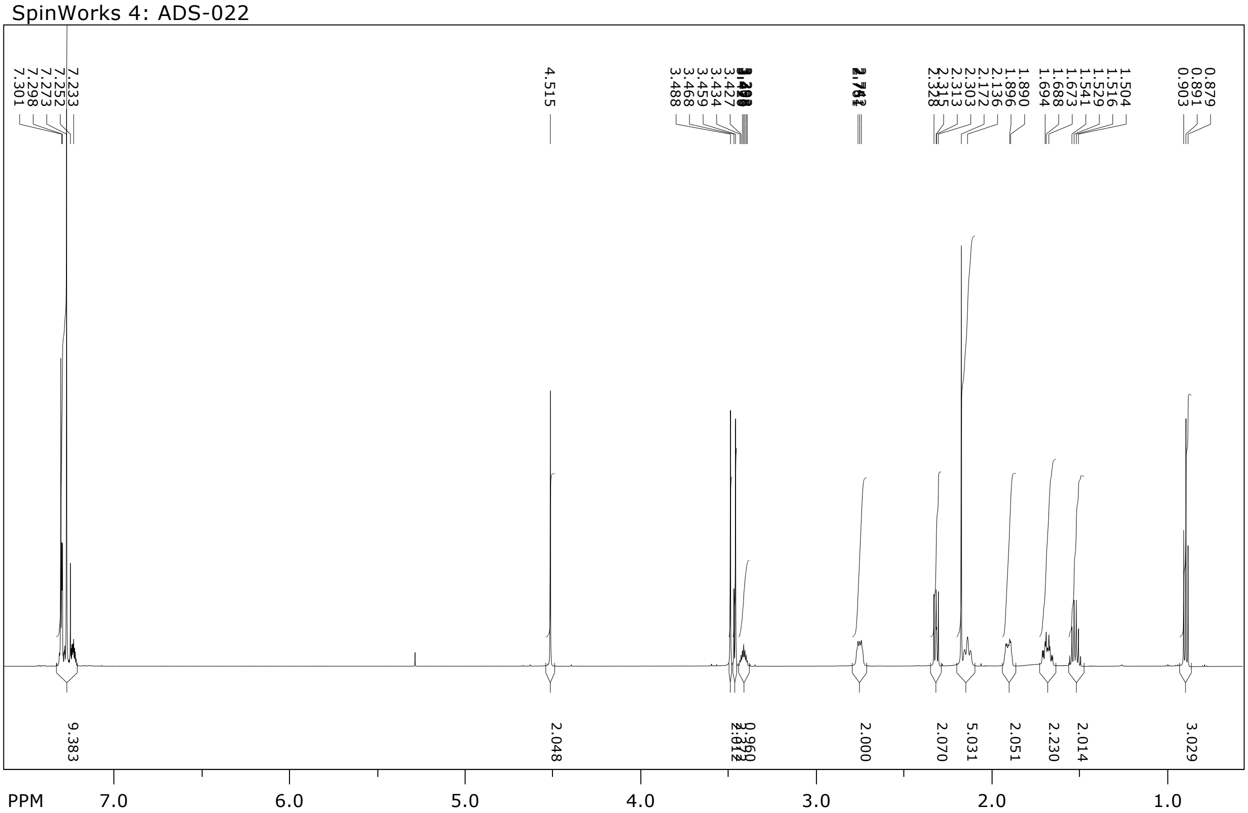

file: F:\FID UMED\bo-140\1\fid expt: <zg30>
transmitter freq.: 600.263707 MHz
time domain size: 65536 points
width: 12335.53 Hz = 20.5502 ppm = 0.188225 Hz/pt
number of scans: 16

freq. of 0 ppm: 600.260023 MHz
processed size: 32768 complex points
LB: 0.000 GF: 0.0000

Figure S3.^{1}H NMR ($CDCl_3$, 600MHz) spectra of compound ADS022 base.

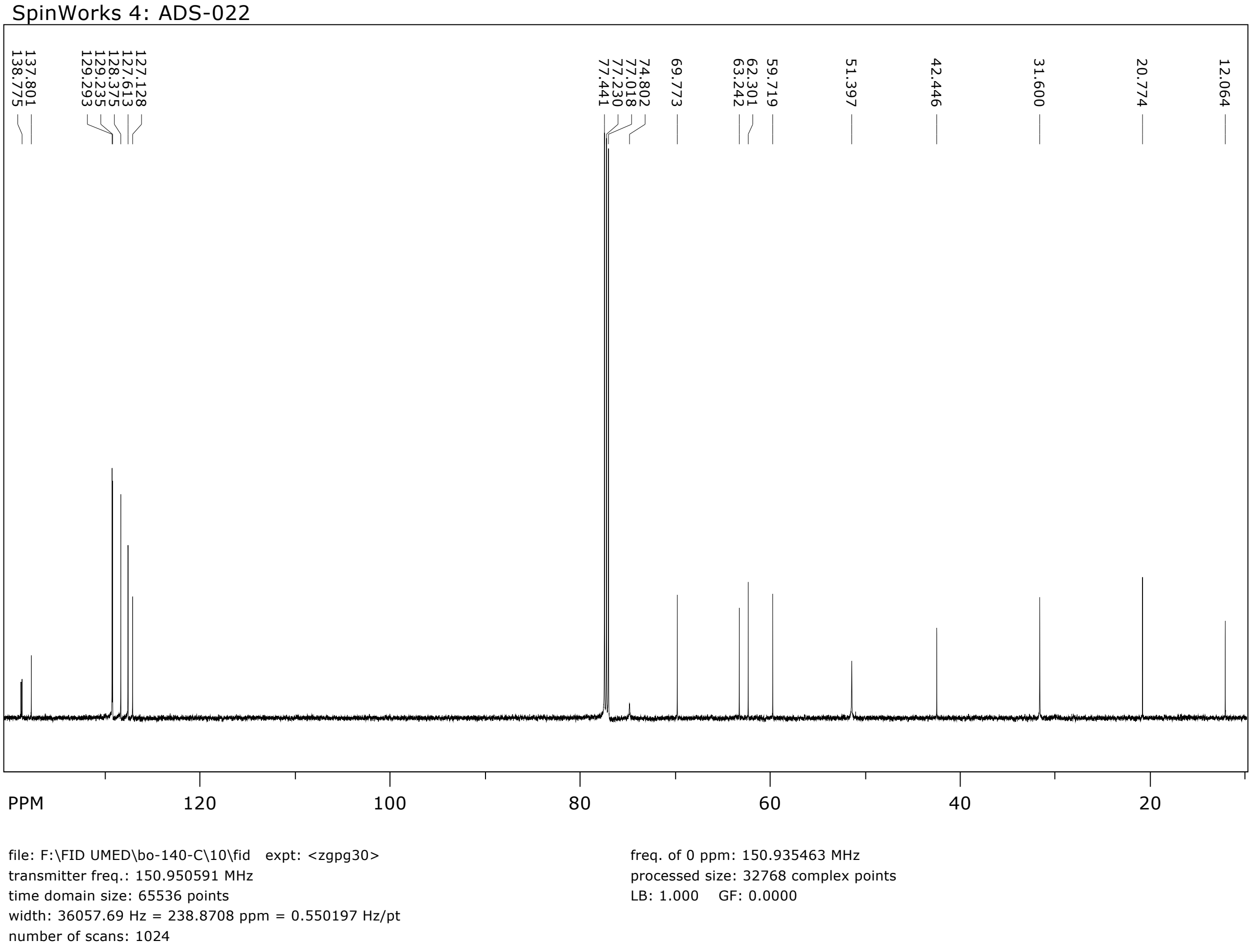

Figure S4. ^{13}C NMR (CDCl$_3$, 150 MHz) spectra of compound ADS022 base.

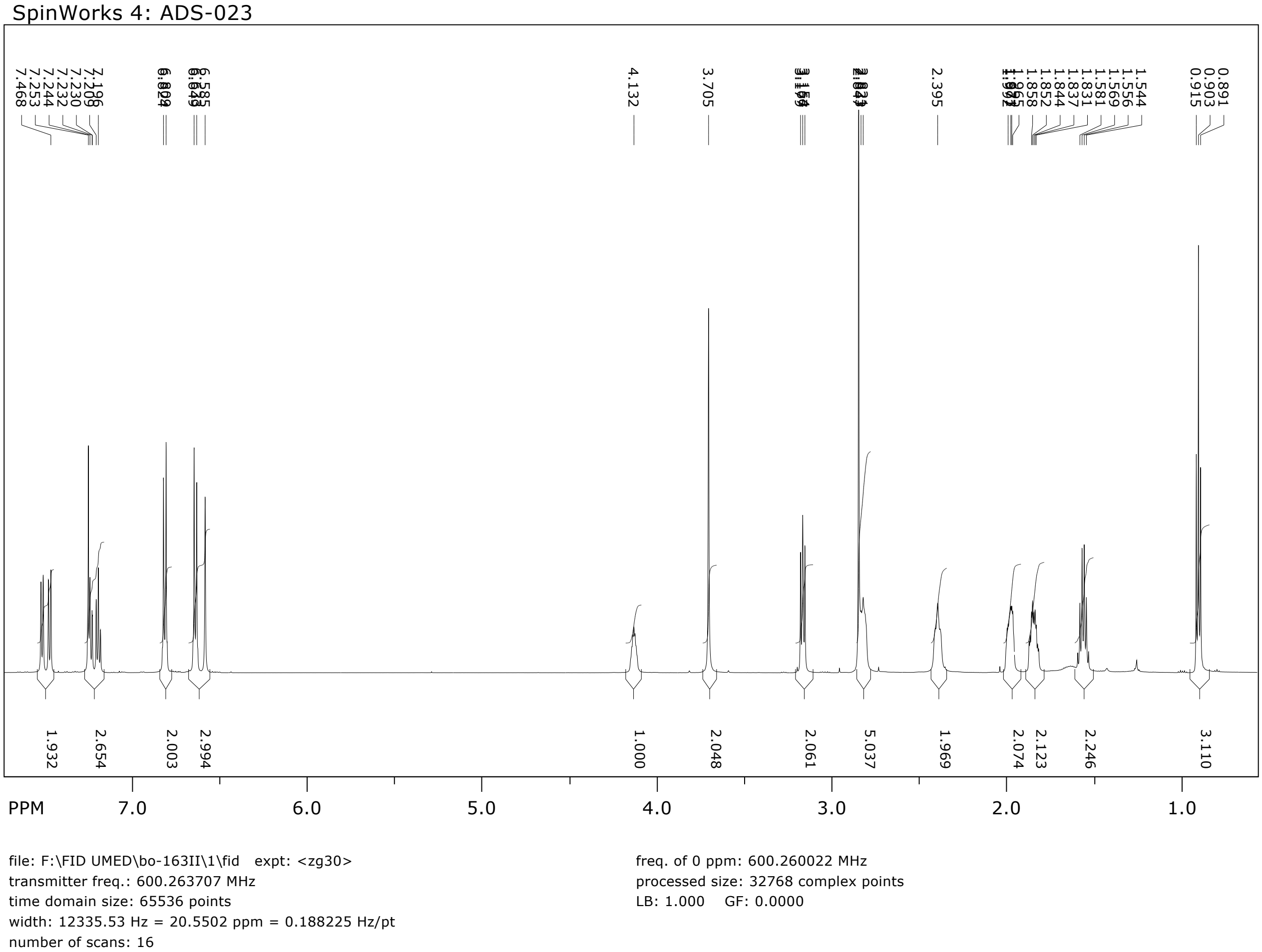

Figure S5.¹H NMR (CDCl$_3$, 600MHz) spectra of compound ADS023 base.

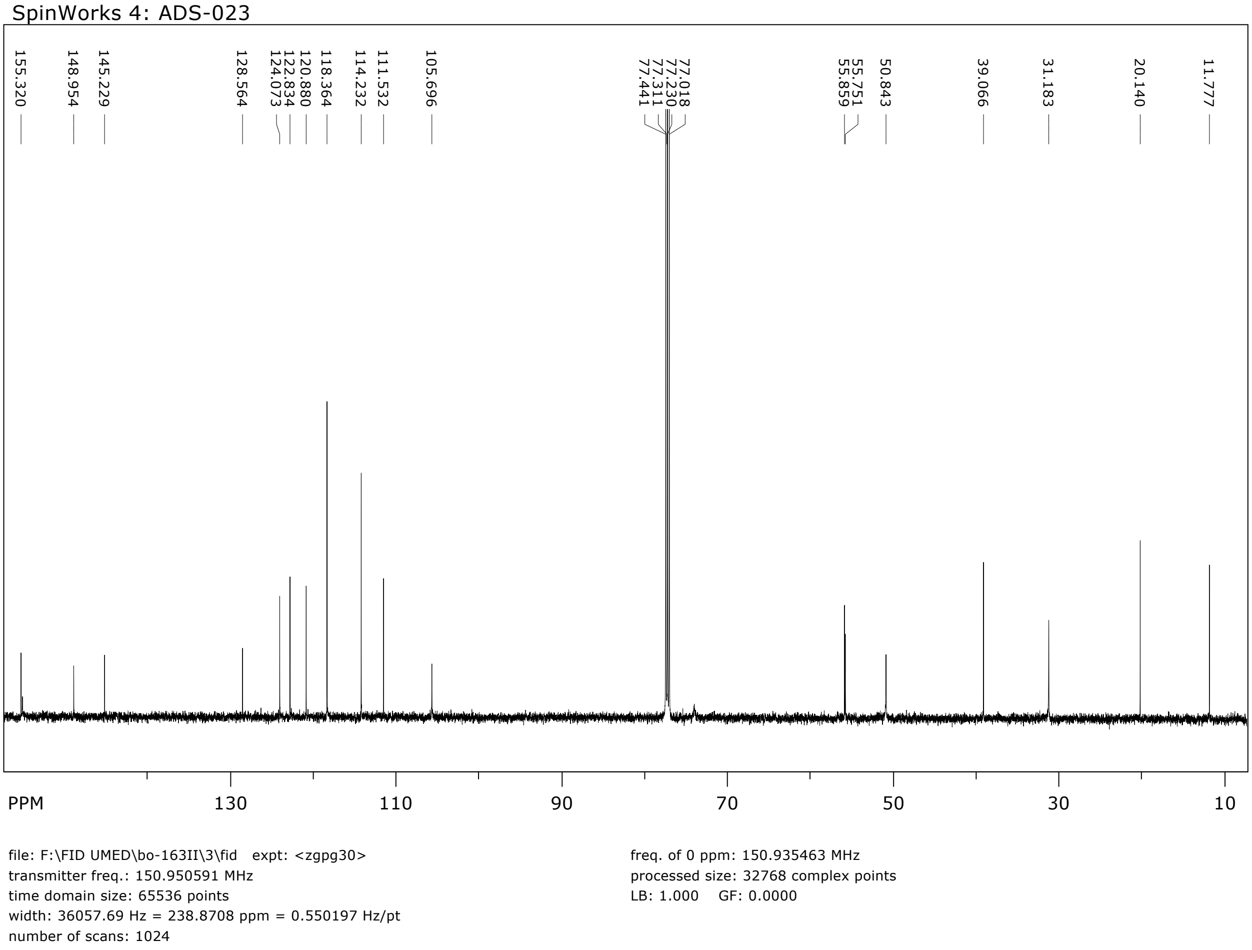

Figure S6. ^{13}**C NMR (CDCl$_3$, 150 MHz) spectra of compound ADS023 base.**

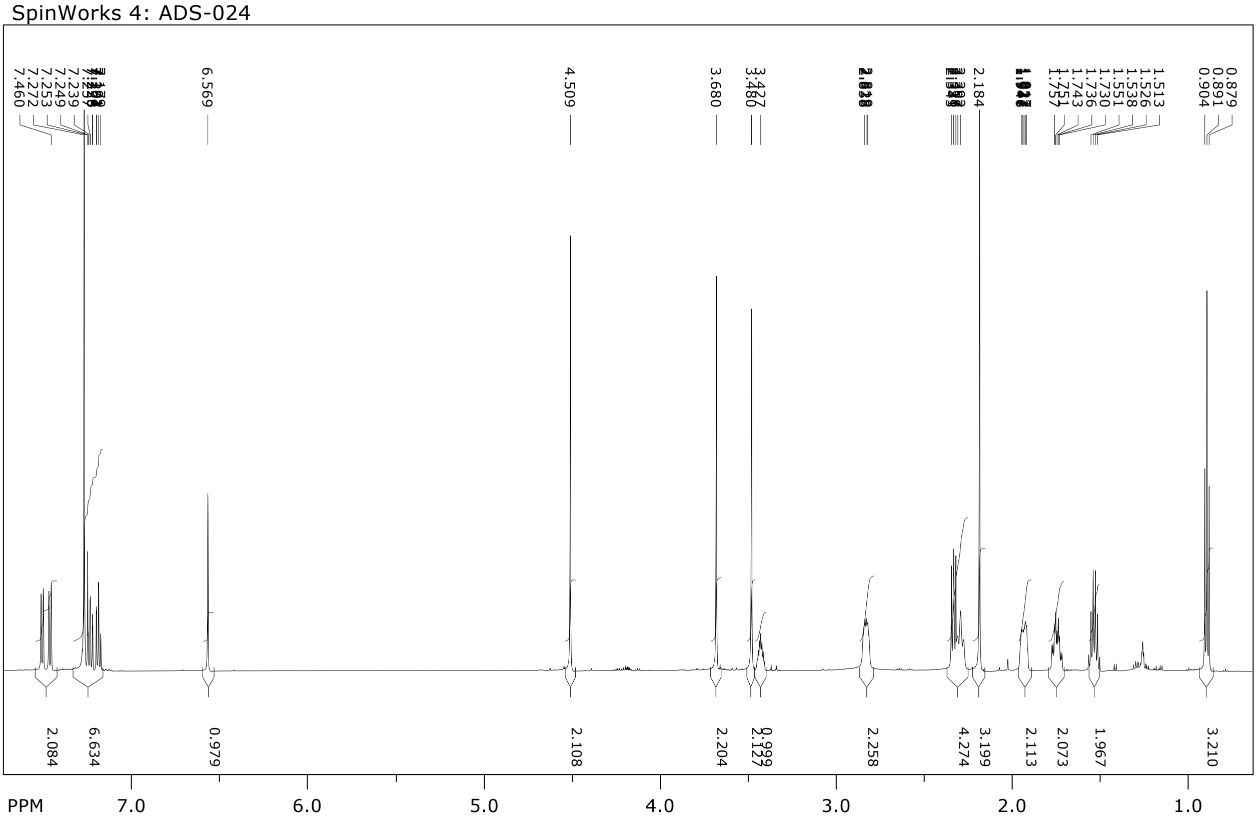

file: F:\FID UMED\bo-193-2\10\fid expt: <zg30>
transmitter freq.: 600.263707 MHz
time domain size: 65536 points
width: 12335.53 Hz = 20.5502 ppm = 0.188225 Hz/pt
number of scans: 16

freq. of 0 ppm: 600.260024 MHz
processed size: 32768 complex points
LB: 0.300 GF: 0.0000

Figure S7.^{1}H NMR ($CDCl_3$, 600MHz) spectra of compound ADS024 base.

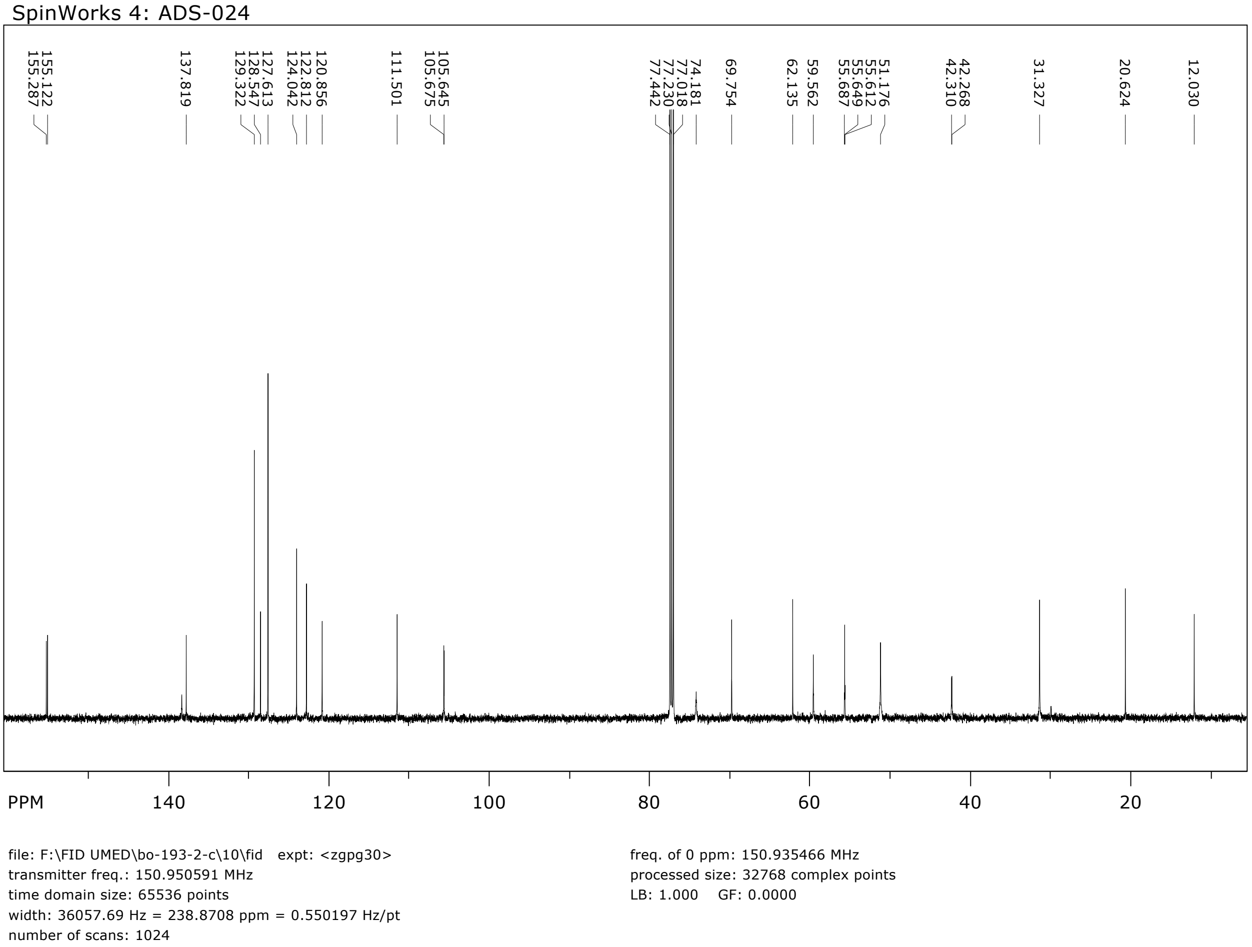

Figure S8. ^{13}C NMR (CDCl$_3$, 150 MHz) spectra of compound ADS024 base.

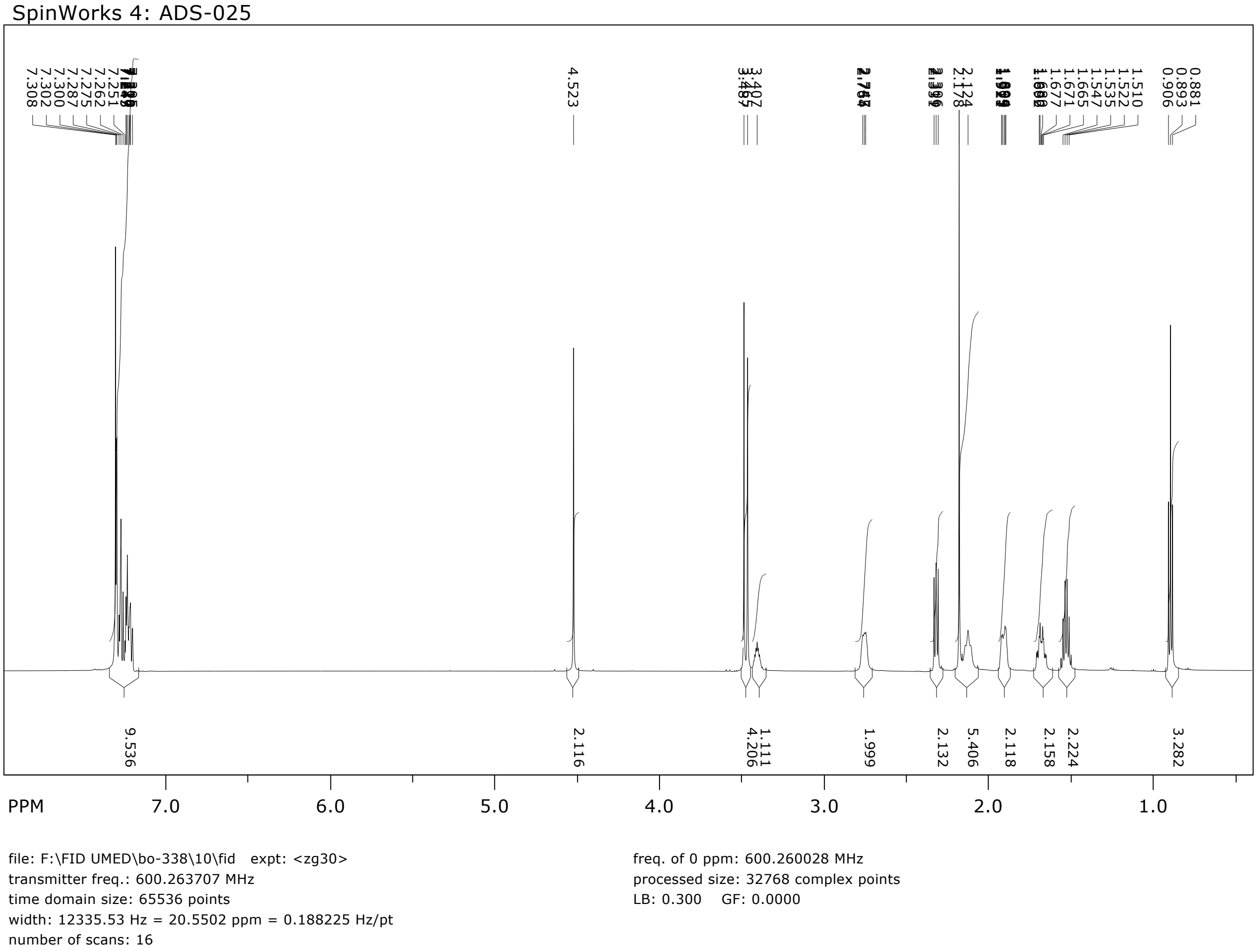

Figure S9.^{1}H NMR ($CDCl_3$, 600MHz) spectra of compound ADS025 base.

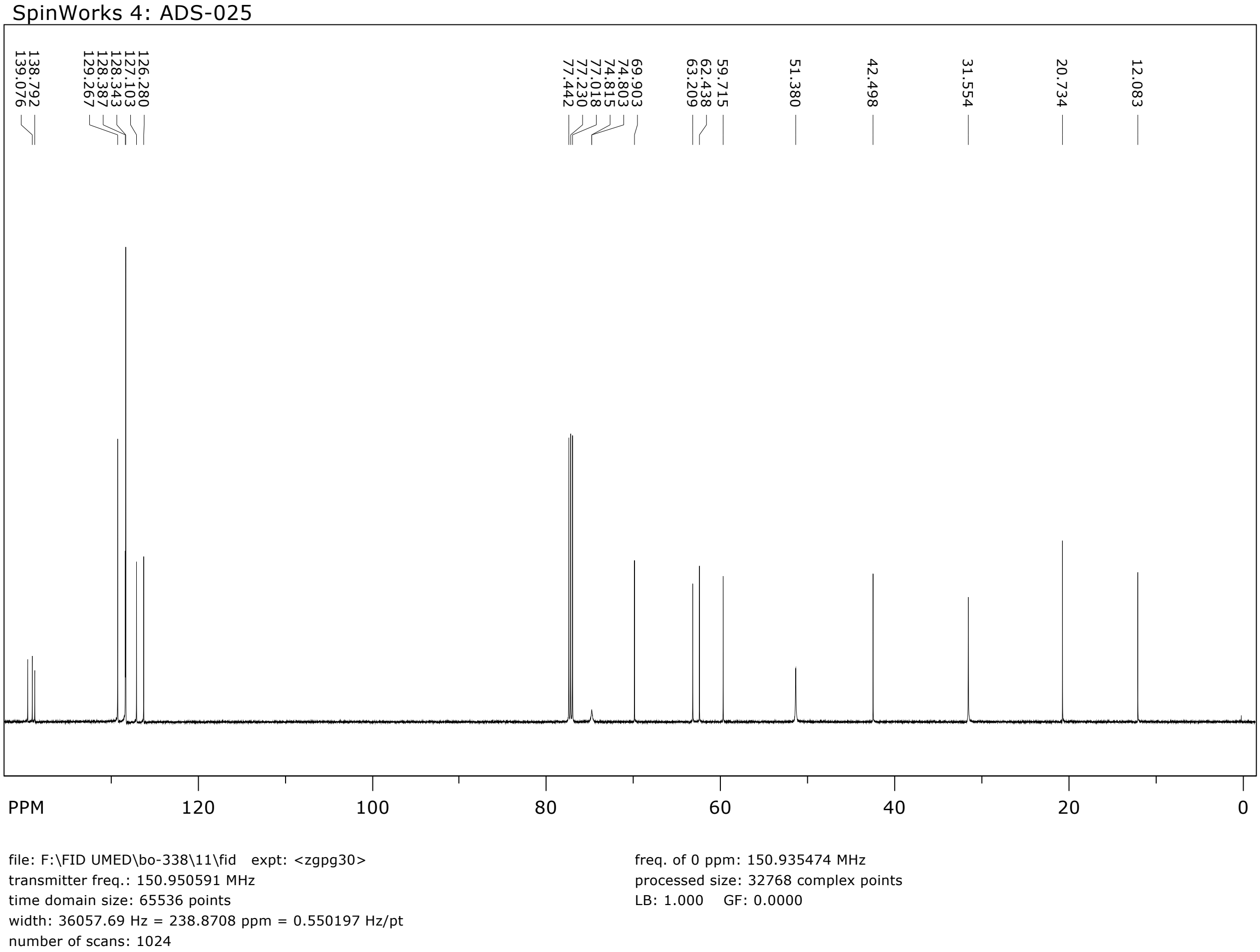

Figure S10. ^{13}C NMR (CDCl$_3$, 150 MHz) spectra of compound ADS025 base.

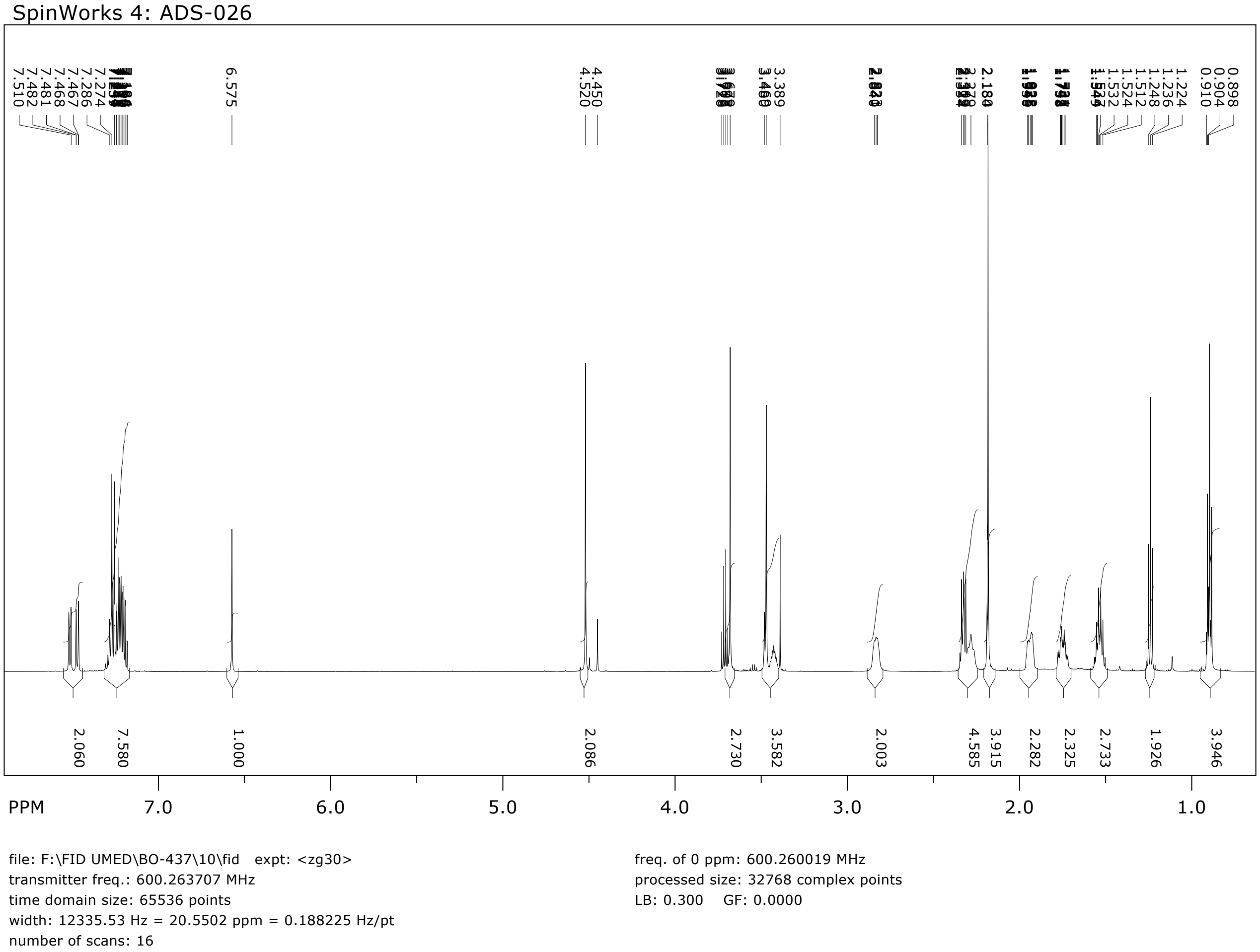

Figure S11.^{1}H NMR ($CDCl_3$, 600MHz) spectra of compound ADS026 base.

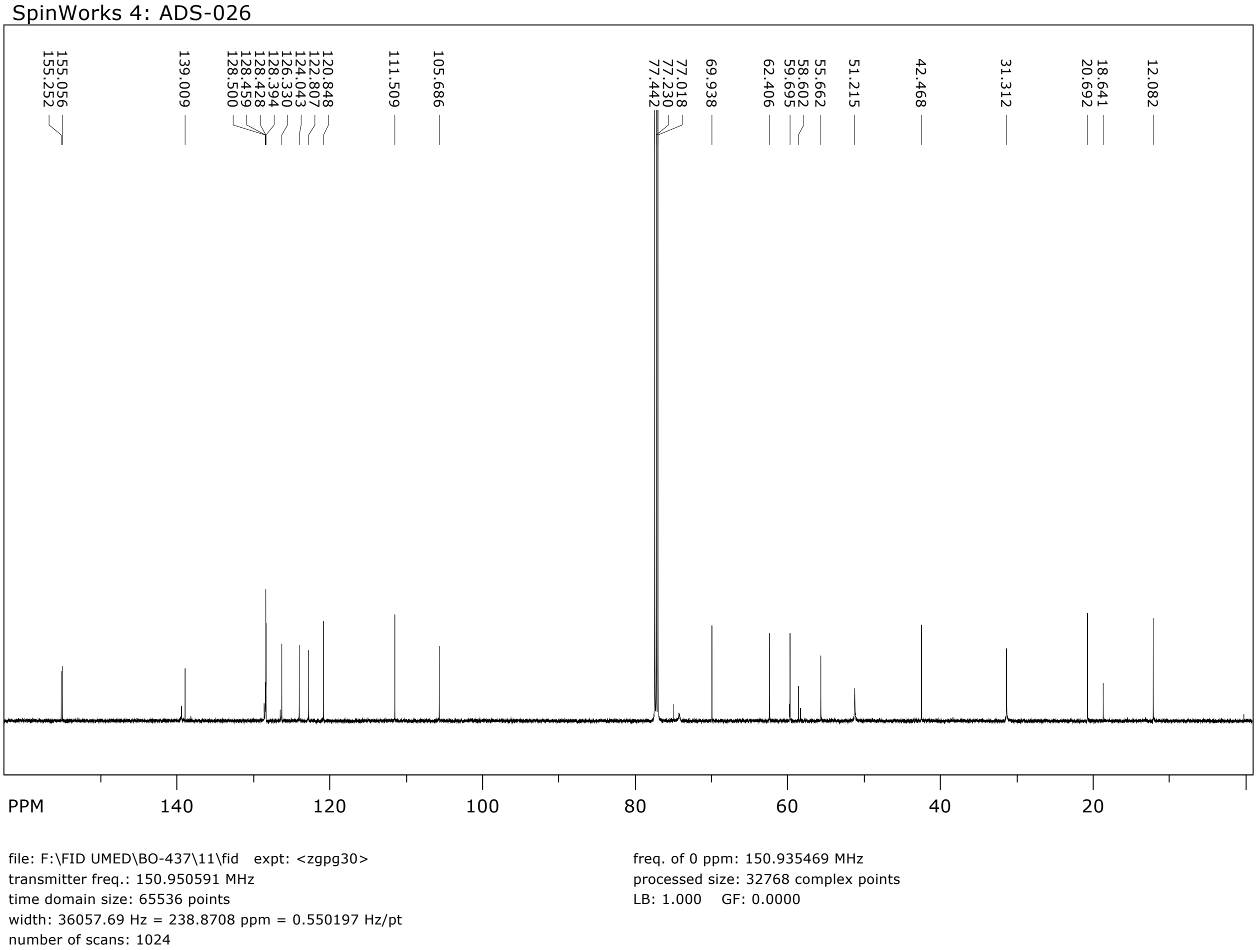

Figure S12. **^{13}C NMR (CDCl$_3$, 150 MHz) spectra of compound ADS026 base.**

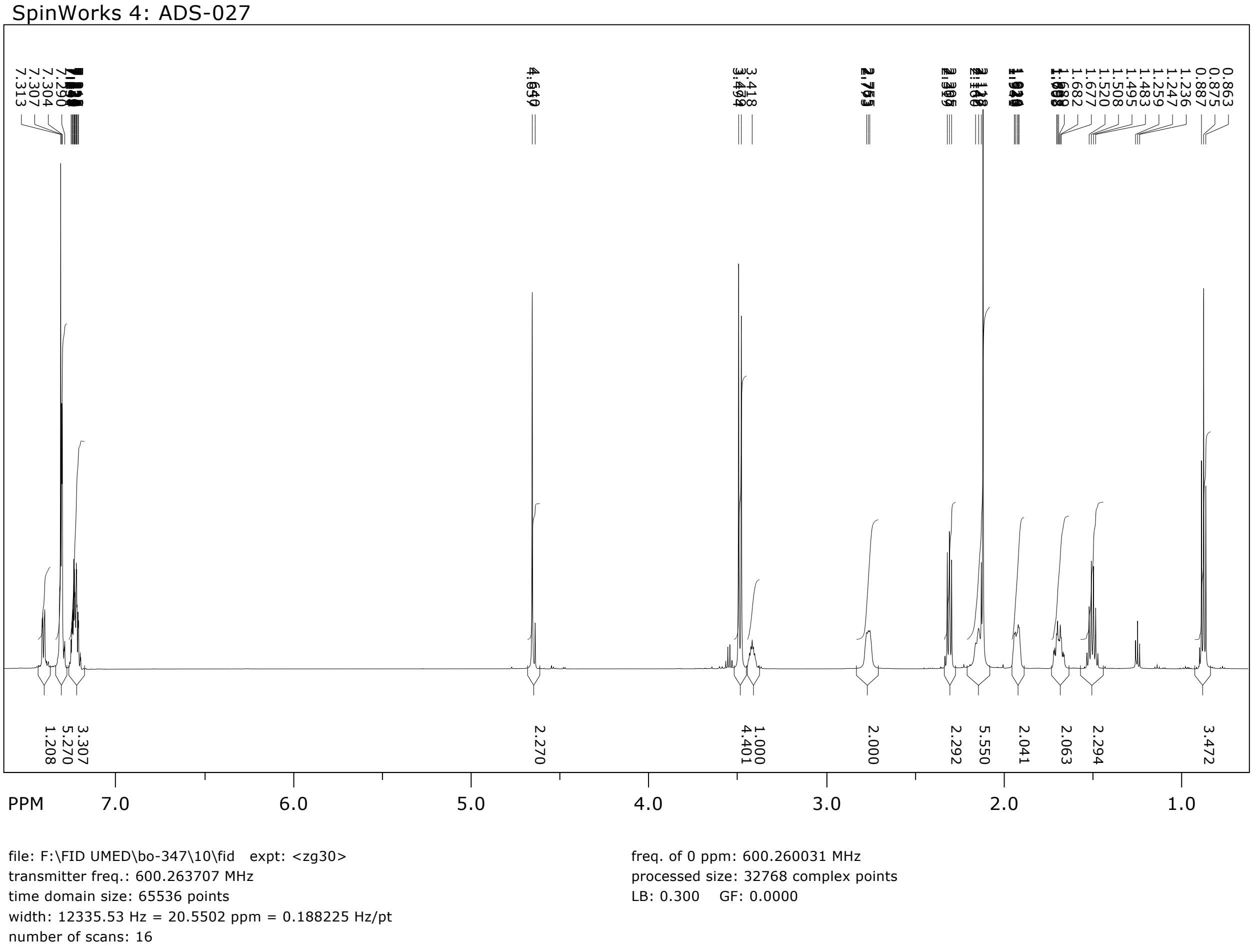

Figure S13.^{1}H NMR ($CDCl_3$, 600MHz) spectra of compound ADS027 base.

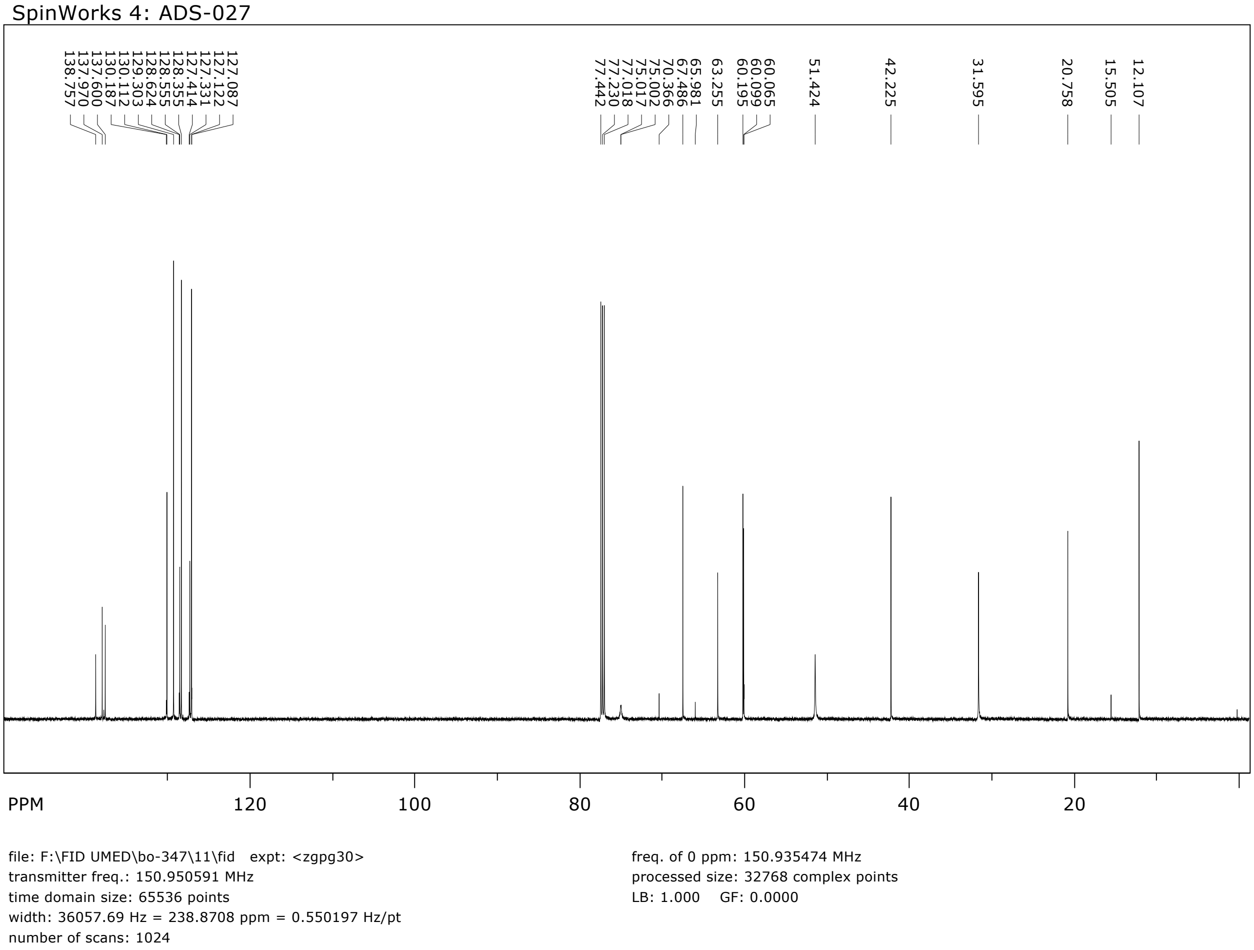

Figure S14. ^{13}C **NMR ($CDCl_3$, 150 MHz) spectra of compound ADS027 base.**

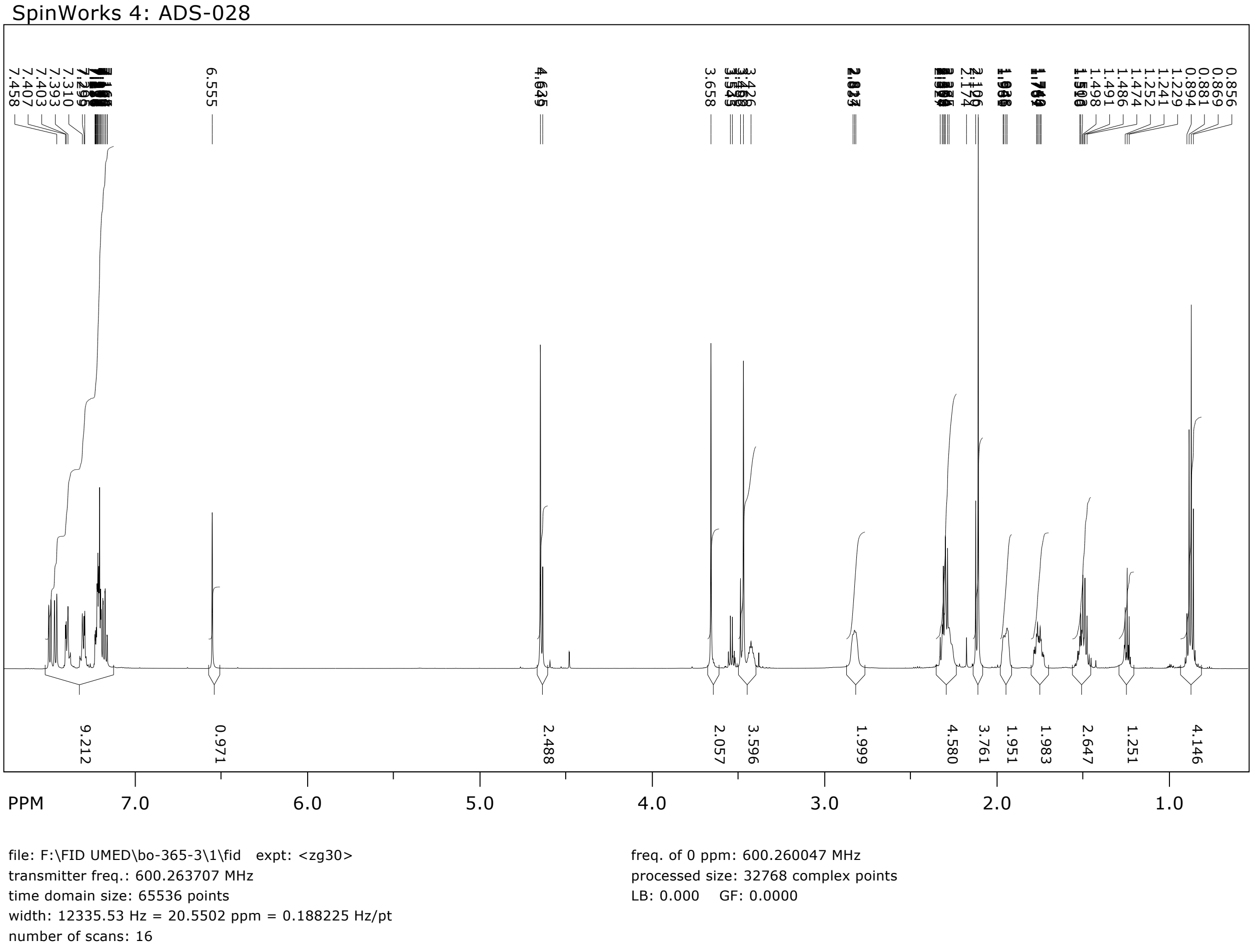

Figure S15.^{1}H NMR ($CDCl_3$, 600MHz) spectra of compound ADS028 base.

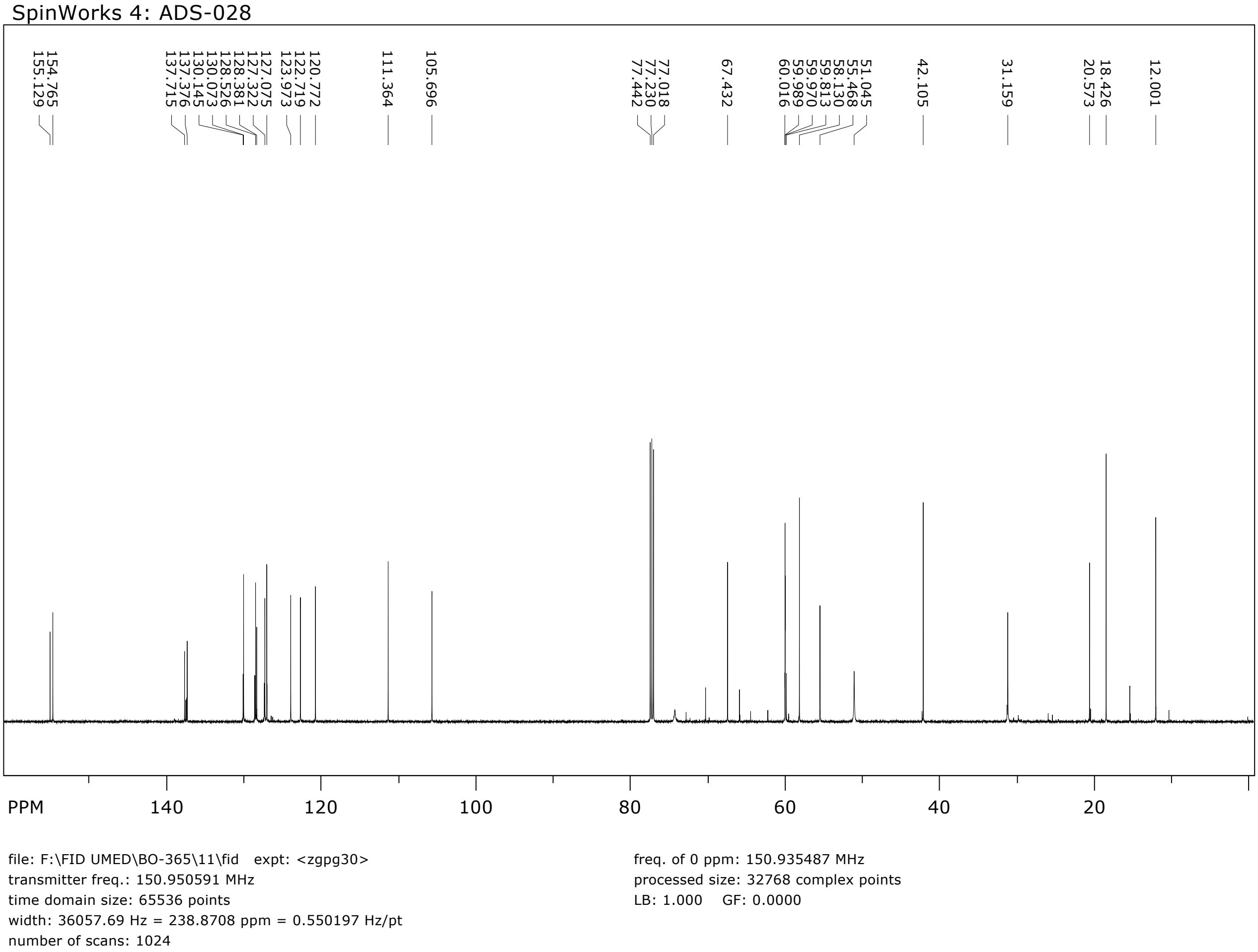

Figure S16. ^{13}C NMR (CDCl$_3$, 150 MHz) spectra of compound ADS028 base.

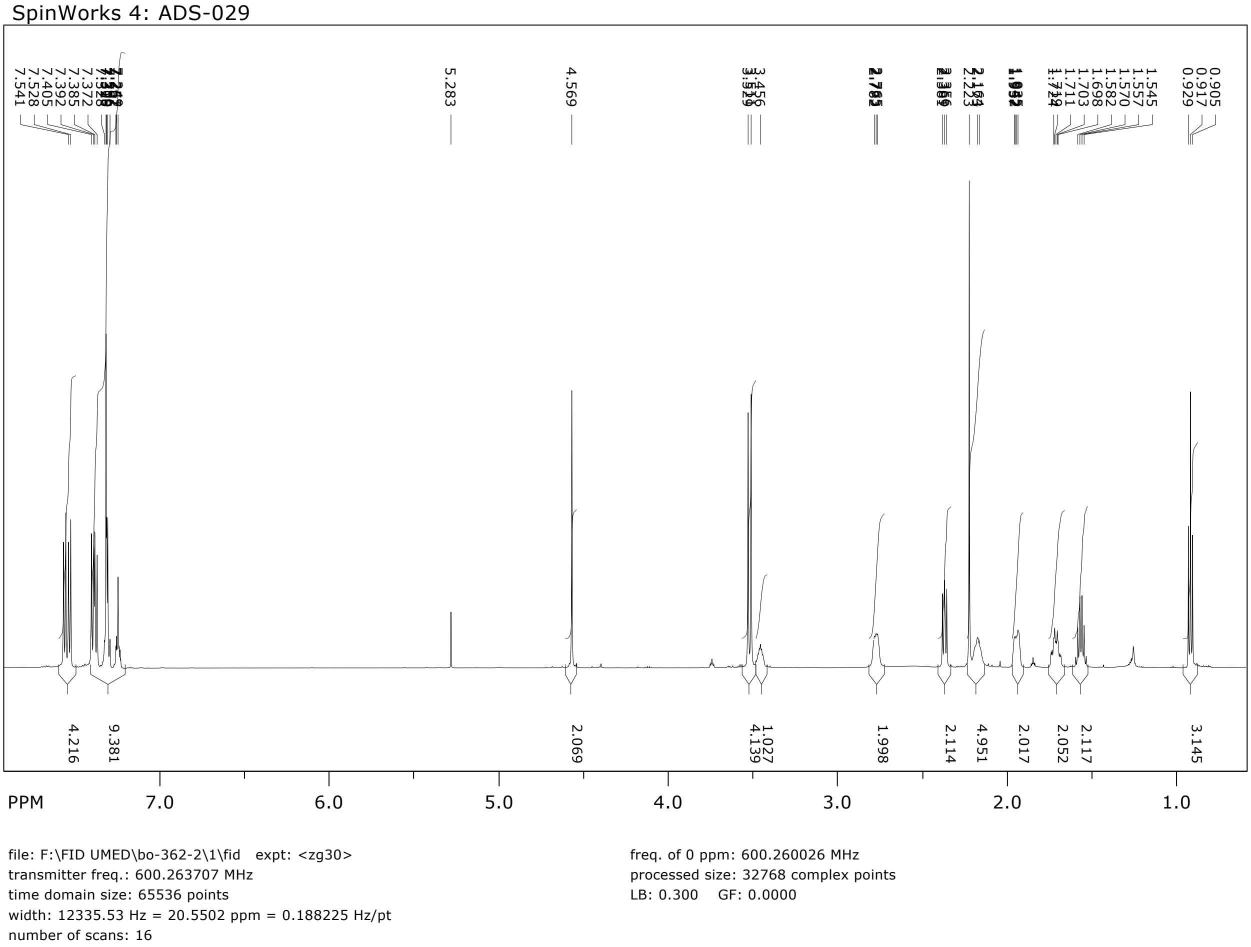

Figure S17.^{1}H NMR (CDCl$_3$, 600MHz) spectra of compound ADS029 base.

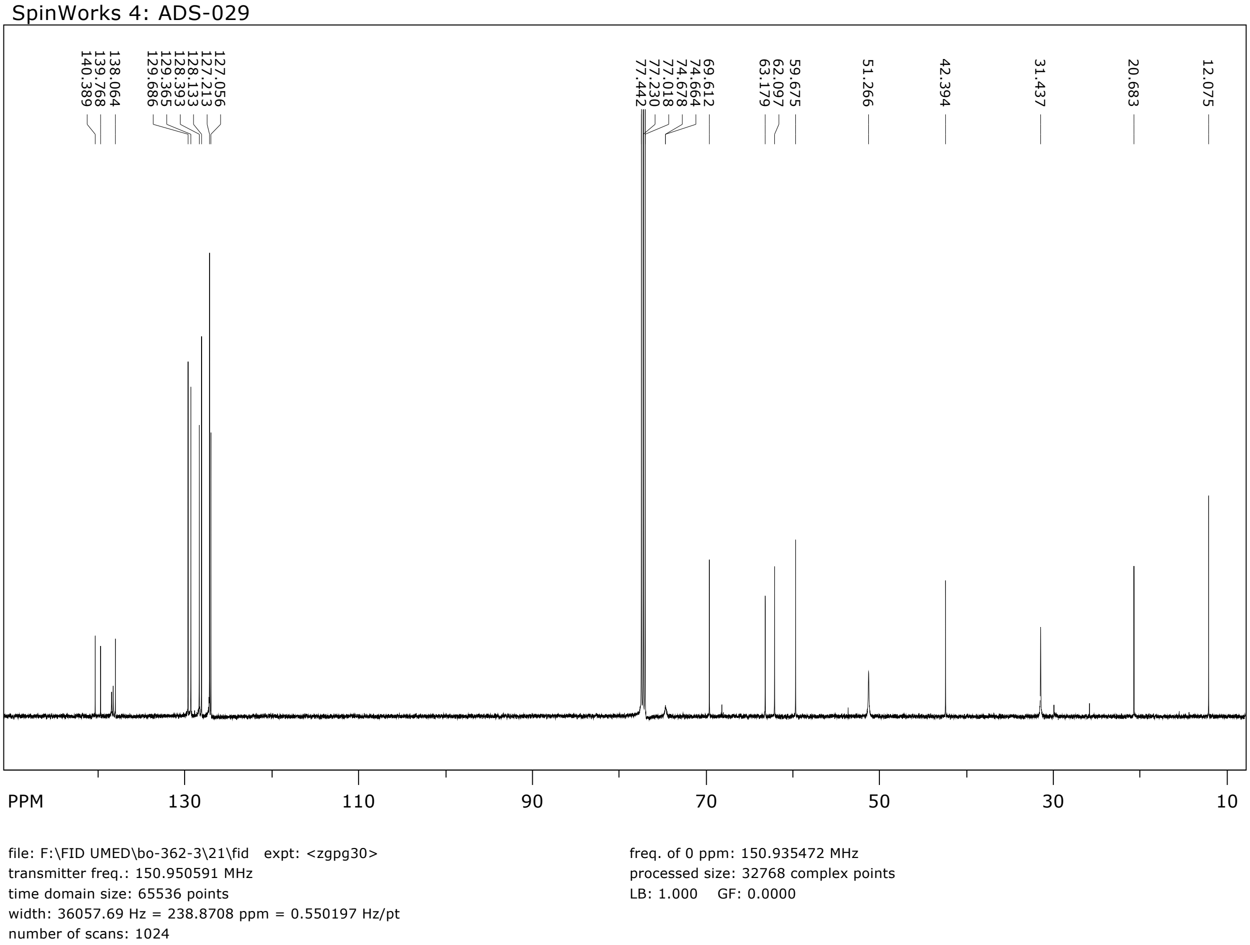

Figure S18. ^{13}**C NMR (CDCl$_3$, 150 MHz) spectra of compound ADS029 base.**

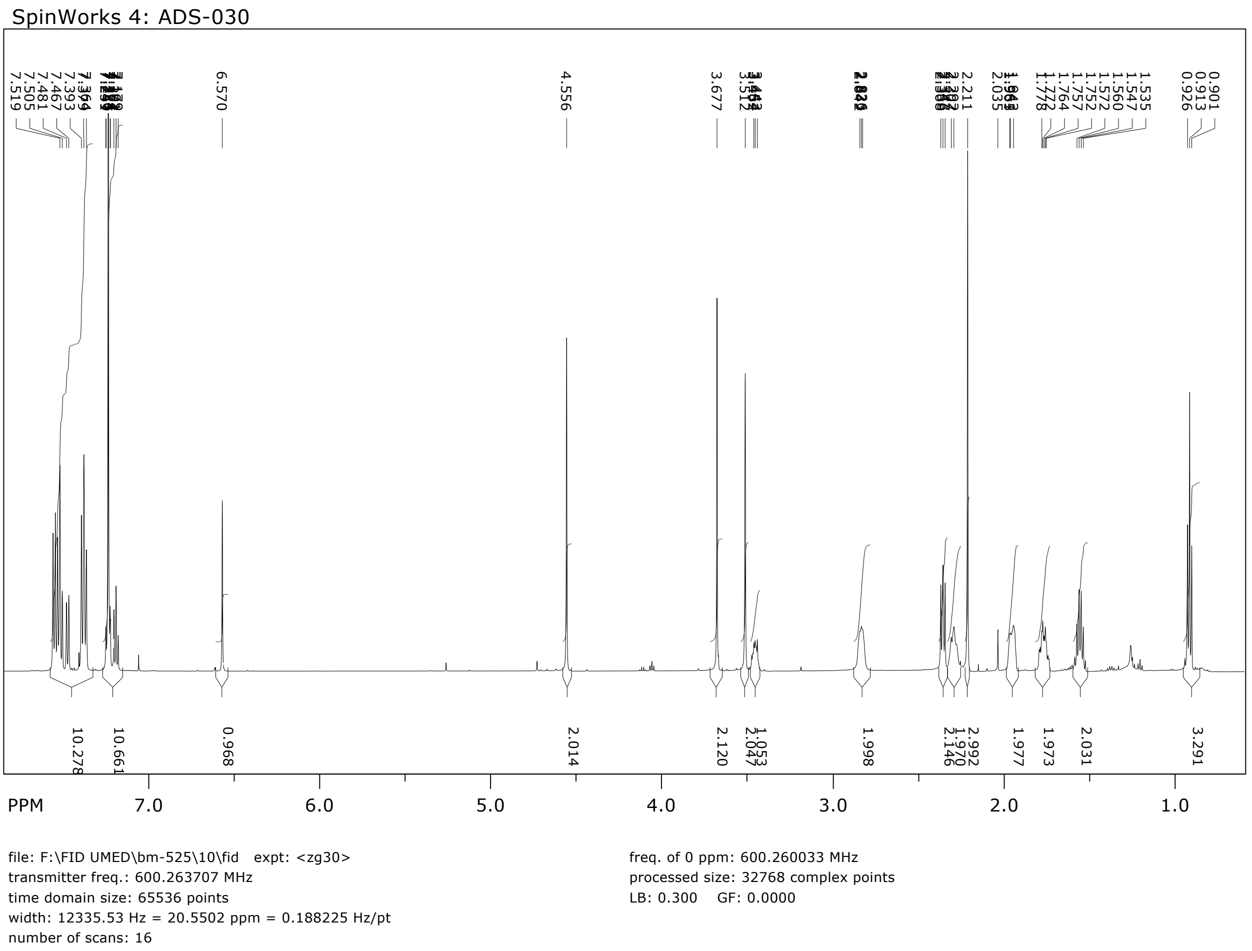

Figure S19.^{1}H NMR ($CDCl_3$, 600MHz) spectra of compound ADS030 base.

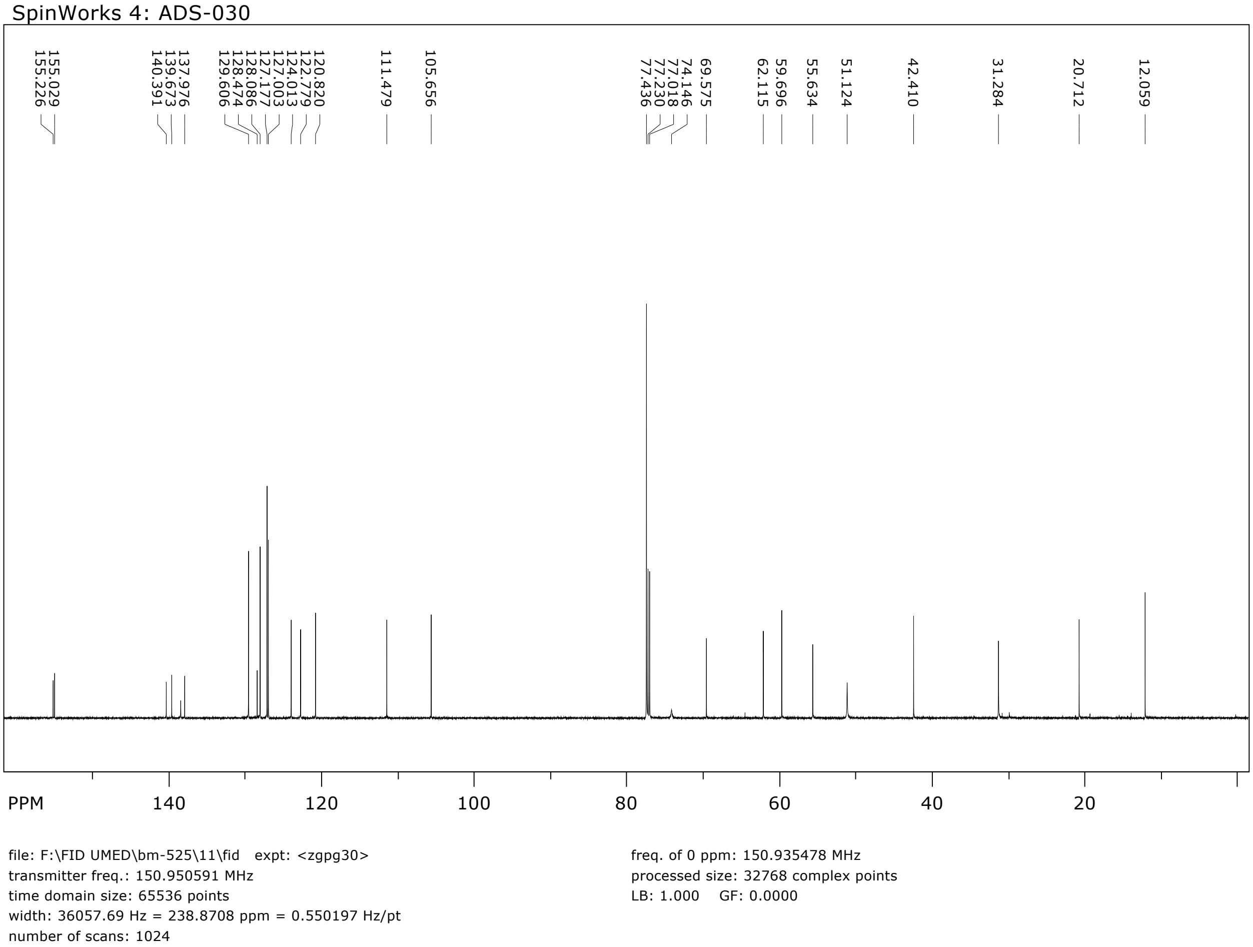

Figure S20 ^{13}C NMR (CDCl$_3$, 150 MHz) spectra of compound ADS030 base.

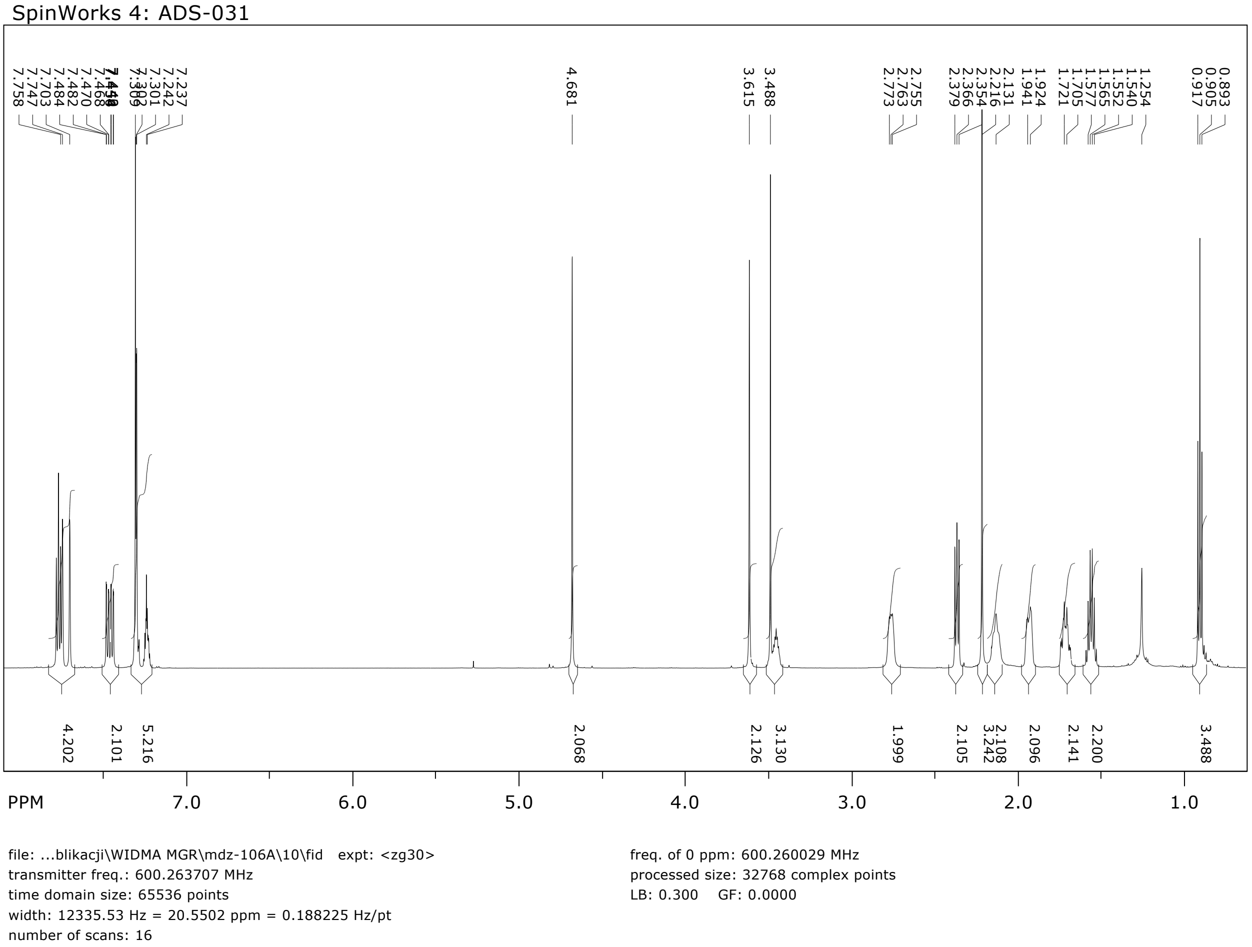

Figure S21.^{1}H NMR ($CDCl_3$, 600MHz) spectra of compound ADS031 base.

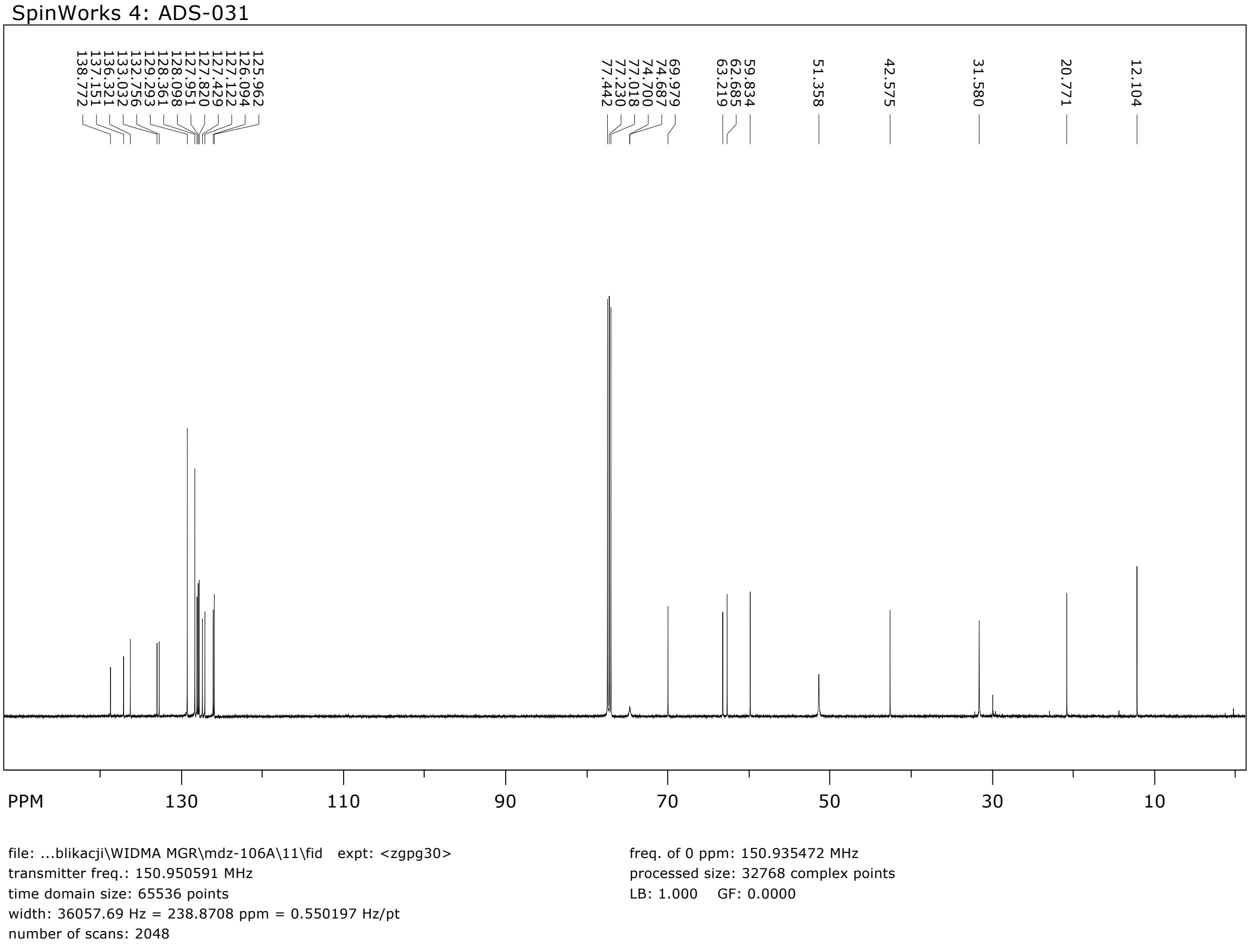

Figure S22. ^{13}**C NMR (CDCl$_3$, 150 MHz) spectra of compound ADS031 base.**

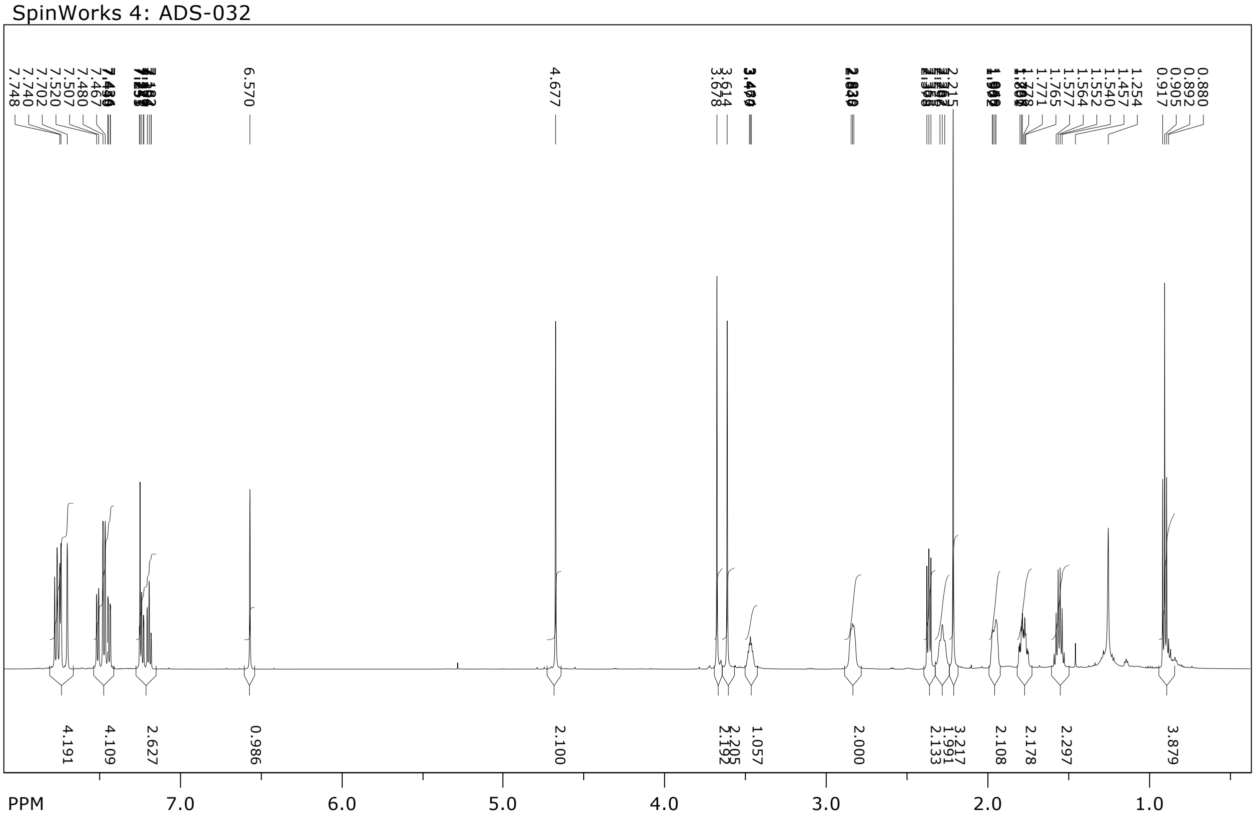

file: ...blikacji\WIDMA MGR\mdz-106B\10\fid expt: <zg30>
transmitter freq.: 600.263707 MHz
time domain size: 65536 points
width: 12335.53 Hz = 20.5502 ppm = 0.188225 Hz/pt
number of scans: 16

freq. of 0 ppm: 600.260024 MHz
processed size: 32768 complex points
LB: 0.300 GF: 0.0000

Figure S23.^{1}H NMR ($CDCl_3$, 600MHz) spectra of compound ADS032 base.

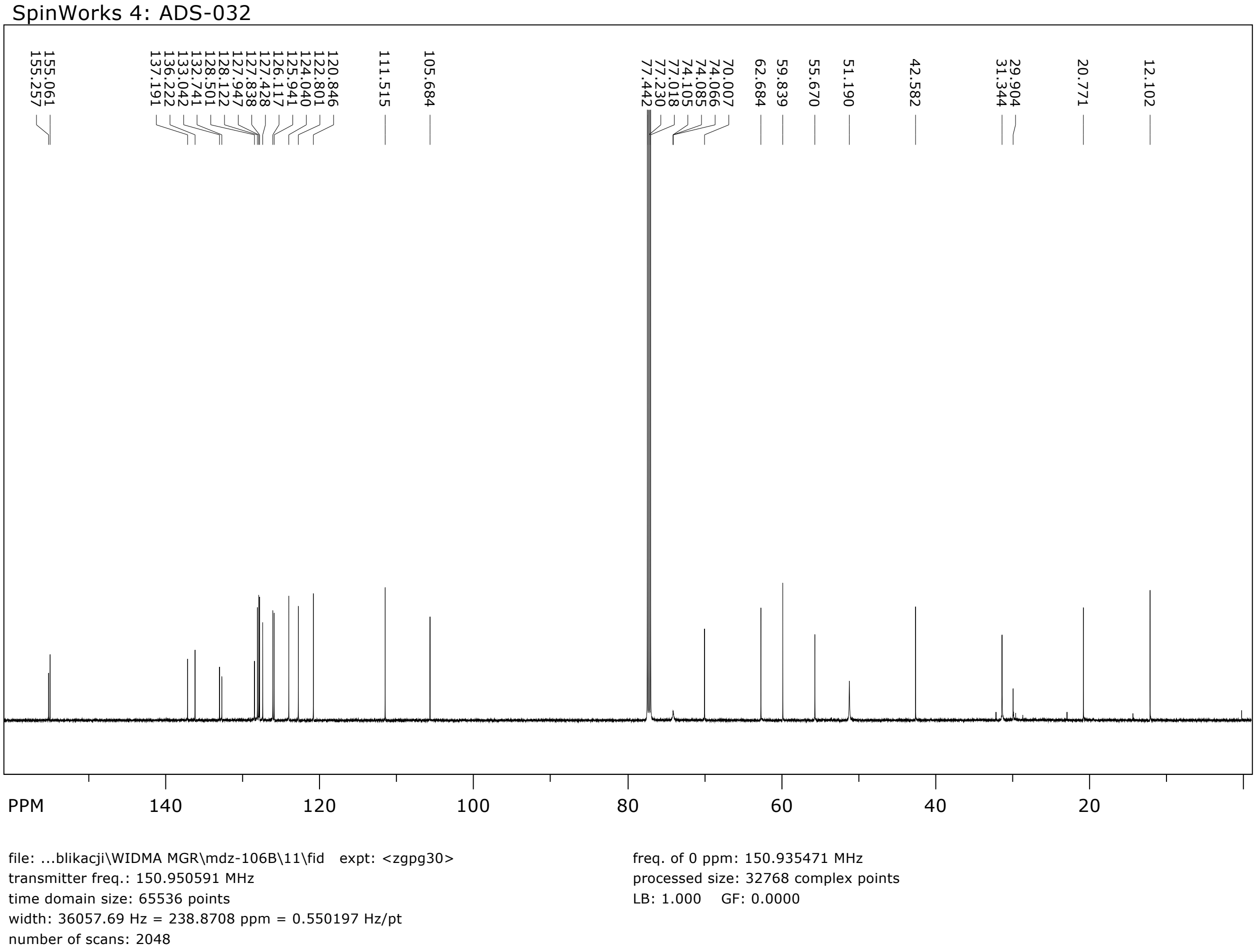

Figure S24. ^{13}C NMR ($CDCl_3$, 150 MHz) spectra of compound ADS032 base.

3. Pharmacological assay results.

3.1. *Ex vivo* assay for histamine H_3R receptor antagonists on guinea pig ileum.

Male guinea pigs, weighing 300-400 g were euthanized by a blow to the neck. Following this, a 20-30 cm length of the distal ileum, apart from the terminal 5 cm was rapidly removed and placed in phosphate buffer at room temperature (pH 7.4) containing (mM) NaCl (136.9); KCl (2.6); KH_2PO_4 (1.47); Na_2HPO_4 (9.58) and indomethacin (Sigma-Aldrich, St. Louis, MO, USA) ($1 \cdot 10^{-6}$ mol/L). The intraluminal content was rinsed and the isolated intestine was cut into 1.5-2 cm segments. The preparations were mounted between two platinum electrodes isotonically in a 20 mL organ bath filled with Krebs buffer: composition (mM) NaCl (118); KCl (5.6); $MgSO_4$ (1.18); $CaCl_2$ (2.5); $NaH_2PO_4 \cdot H_2O$ (1.28); $NaHCO_3$ (25); glucose (5.55) and indomethacin ($3 \cdot 10^{-7}$ mol/L). The solution was continuously bubbled with a 95 % O_2: 5 % CO_2 mixture and maintained at 37 °C under a constant load of 1.0 g (Hugo Sachs Hebel-Messvorsatz (Tl-2)/HF-modem; Hugo Sachs Elektronik, Hugstetten, Germany) connected to a pen recorder (Kipp & Zonen BD41, Delft, Holland). During an equilibration period of 60 min, the Krebs buffer was changed every 10 min. The preparations were then continuously stimulated at 15-20 V at a frequency of 0.1 Hz for a duration of 0.5 ms, with rectangular-wave electrical pulses (Grass Stimulator S-88; Grass Instruments Co., Quincy, Massachusetts, USA). After about 30min, the twitches were recurrent. Five minutes before (*R*)-(−)-α-methylhistamine (RAMH) (Toronto Research Chemicals Inc., North York, Canada), administration, pyrilamine (Sigma-Aldrich, St. Louis, MO, USA) ($1 \cdot 10^{-5}$ mol/L concentration in organ bath) was added. The first cumulative concentration-response curve was determined to RAMH (10 nM – 10 mM) at an increasing concentration spaced by three or 3.3-fold. The second to fourth curve was measured against increasing antagonist concentration (incubation time 20 min). The pA_2-values were calculated according to Arunlakshana and Schild [5]. Statistical analysis was carried out with the Student's t-test. In all tests, a $p<0.05$ was

considered statistically significant. The pA_2 values were compared with the affinity of thioperamide (Sigma-Aldrich, St. Louis, MO, USA).

3.2. *h*H_3R radioligand displacement binding assay.

The radioligand displacement binding assay was performed in membrane fractions of HEK-293 cells stably expressing hH_3R. Cell cultivation and membrane preparation was performed according to Kottke et al [6]. For the radioligand displacement assay, radioactively labeled [^{3}H]Nα-methylhistamine was used at a final concentration of 2 nM (KD = 3.08 nM). The total assay volume was set to 200 µL. The compounds were tested in several appropriate concentrations between 100 µM and 0.1 nM. Pipetting was partly done by Freedom Evo® (Tecan). Pitolisant was used to determine non-specific binding at a concentration of 10 µM. The membrane fraction (20 µg/well), test compounds, and radiolabeled ligand were incubated for 90 minutes at 25 °C while shaking. The bound radioligand was separated from the free radioligand by filtration through GF/B filters pre-treated with 0.3 % (m/v) polyethyleneimine using a cell harvester. Radioactivity was determined by liquid scintillation counting using a MicroBeta® Trilux (Perkin Elmer). The data was obtained in duplicates in at least three independent experiments. Non-specific binding was subtracted from the raw data to calculate specific binding values. The evaluation was performed with GraphPad Prism 6.1 (San Diego, CA, USA) using non-linear regression (one-site competition with a logarithmic scale). The Ki values were calculated from the IC_{50} values using the Cheng-Prusoff equation [7]. The statistical calculations were performed on $-\log(K_i)$. The mean values and 95 % confidence intervals were transformed to nanomolar concentrations.

3.3. Inhibition of *electric eel* AChE and *equine* serum BuChE

The target compounds were tested for their inhibitory potency against cholinesterases using Ellman's protocol, modified for 96-well microplates [8]. All the reagents were purchased from Sigma–Aldrich (Steinheim, Germany). The enzymes were prepared as 5 U/mL aqueous stock solutions and diluted before use to a final concentration of 0.384 U/mL. Then 20 μL of prepared enzyme solutions (AChE or BuChE) were added to the reaction mixture in the wells, containing 25 μL of the target compound (or water in case of blank samples), 200 μL of 0.1 M phosphate buffer (pH=8.0) and 20 μL of 5,5'-dithiobis-(2-nitrobenzoic acid) (DTNB) (0.0025M). All those reagents were preincubated for 5 min at 25 °C. The enzymatic reaction was initiated by the addition of 20 μL of substrate acetylthiocholine iodide (ATC) (0.00375M) or butyrylthiocholine iodide (BTC) (0.00375M) solutions (depending on the enzyme used). After 5 min of incubation, changes in absorbance were measured at 412 nm, using EnSpire multimode microplate reader (PerkinElmer, Waltham, MA, USA). Target compounds were tested at a screening concentration of 10 μM. Percent of enzyme inhibition was calculated based on the formula 100-(S/B)x100, where S and B were the respective enzyme activities with and without the test compound. For the most potent compounds, with at least 60 % of the enzyme inhibitory activity, IC_{50} values were determined. Calculations were based on the absorbance measured at six different concentrations of inhibitor, then converted to % enzyme inhibition, using the formula above. The obtained percentages of enzyme inhibition were plotted against the applied inhibitor concentrations, using nonlinear regression (GraphPad Prism 9; GraphPad Software, San Diego, CA, USA). Tacrine was tested as a reference compound. All the experiments were performed in triplicate.

3.4. H_3R intrinsic activity.

Intracellular cAMP accumulation was measured with homogenous TR-FRET immunoassay, using LANCE Ultra cAMP kit (PerkinElmer) and HEK293 cells, stably expressing human histamine H_3 receptor. An antagonist dose-response experiments were performed in a total assay volume of 20 µl in white 384-well plates, using 750 cells/well. (R)-(-)-α-Methylhistamine (30 nM) and forskolin (10 µM) were added simultaneously to cell suspension together with tested compound in appropriate concentrations (in range 0.0003-10 µM). Cells stimulation was performed for 30 min at room temperature. After incubation period, five microliters of europium (Eu) chelate-labeled cAMP tracer and 5 µl of ULight-labeled anti-cAMP mAb working solutions were added, mixed and incubated for 1 h. TR-FRET signal was read on Spark microplate reader (Tecan Life Sciences). Measured TR-FRET, which was inversely proportional to cAMP quantities in the sample, was then normalized and presented as % of maximal cAMP accumulation in the assay determined for the forskolin alone-stimulated cells. Sigmoidal dose-response curve fitting was performed with use of Prism software (v 8.4.3, GraphPad Software) and IC_{50} values were extracted from the fitting. K_b values of the compounds was estimated according to the Leff-Dougall variant of the Cheng-Prusoff equation $K_b = IC_{50}/((2 + ([Ag]/[EC_{50}])^n)^{1/n} - 1)$, where IC_{50} is the concentration of antagonist that inhibits agonist response by a 50%; [Ag] is the concentration of agonist employed in the assay, $[EC_{50}]$ is the agonist EC_{50} value in the assay and n is the Hill slope of the concentration-response curve of the agonist [9]. Showed results represent the mean of three separate experiments, each performed in triplicates.

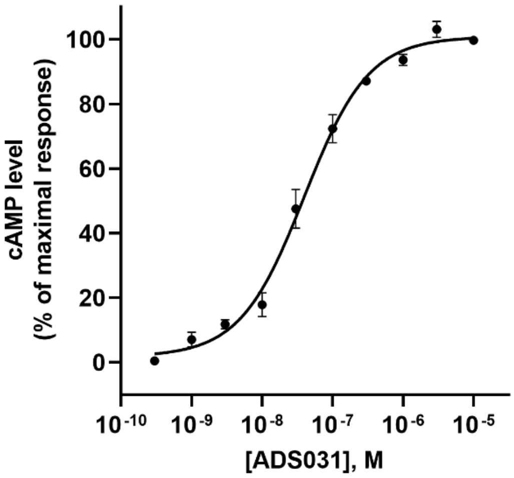

Figure S25. H_3R intrinsic activity of ADS031

3.5. Physiochemical parameters.

Table S1. Physiochemical parameters determined at the SwissADME web tool [10]

Cpd.	wlogP	mlogP	xlogP	M	Num. rotatable bonds	Num. H-bond acceptors	Num. H-bond donors	Molar Refractivity	TPSA
**ADS021**	4.04	3.56	5.00	338.49	7	2	0	109.97	15.71
**ADS023**	4.79	3.25	5.43	378.51	7	3	0	119.75	28.85
**ADS022**	3.87	3.45	4.39	366.54	9	3	0	117.61	15.71
**ADS024**	4.62	3.13	4.83	406.56	9	4	0	127.38	28.85
**ADS025**	3.87	3.45	4.39	366.54	9	3	0	117.61	15.71
**ADS026**	4.62	3.13	4.83	406.56	9	4	0	127.38	28.85
**ADS027**	3.87	3.45	4.39	366.54	9	3	0	117.61	15.71
**ADS028**	4.62	3.13	4.83	406.56	9	4	0	127.38	28.85
**ADS029**	5.54	4.42	6.01	442.64	10	3	0	143.04	15.71
**ADS030**	6.29	4.07	6.45	482.66	10	4	0	152.82	28.85
**ADS031**	5.03	4.07	5.64	416.60	9	3	0	135.11	15.71
**ADS032**	5.77	3.73	6.08	456.62	9	4	0	144.89	28.85

4. References.

(1) Yamane, T.; Hanaoka, K.; Muramatsu, Y.; Tamura, K.; Adachi, Y.; Miyashita, Y.; Hirata, Y.; Nagano, T. Method for Enhancing Cell Penetration of Gd3+-Based MRI Contrast Agents by Conjugation with Hydrophobic Fluorescent Dyes. *Bioconjug Chem* **2011**, *22* (11), 2227–2236. https://doi.org/10.1021/BC200127T.

(2) Ozcan, S.; Kazi, A.; Marsilio, F.; Fang, B.; Guida, W. C.; Koomen, J.; Lawrence, H. R.; Sebti, S. M. Oxadiazole-Isopropylamides as Potent and Noncovalent Proteasome Inhibitors. *J Med Chem* **2013**, *56* (10), 3783–3805. https://doi.org/10.1021/JM400221D.

(3) Tseng, P. W.; Yeh, S. W.; Chou, C. H. Syntheses and Pyrolyses of Benzofuran Analogues of α-Oxo-o- Quinodimethane. A Study on Vinylcarbene-Cyclopropene Rearrangement. *Journal of Organic Chemistry* **2008**, *73* (9), 3481–3485. https://doi.org/10.1021/JO702704E.

(4) Sarabia, F. J.; Ferreira, E. M. Radical Cation Cyclopropanations via Chromium Photooxidative Catalysis. *Org Lett* **2017**, *19* (11), 2865–2868. https://doi.org/10.1021/ACS.ORGLETT.7B01095.

(5) Arunlakshana, O.; Schild, H. O. Some Quantitative Uses Of Drug Antagonists. *Br J Pharmacol Chemother* **1959**, *14* (1), 48–58. https://doi.org/10.1111/j.1476-5381.1959.tb00928.x.

(6) Kottke, T.; Sander, K.; Weizel, L.; Schneider, E. H.; Seifert, R.; Stark, H. Receptor-Specific Functional Efficacies of Alkyl Imidazoles as Dual Histamine H3/H4 Receptor Ligands. *Eur J Pharmacol* **2011**, *654* (3), 200–208. https://doi.org/10.1016/j.ejphar.2010.12.033.

(7) Yung-Chi, C.; Prusoff, W. H. Relationship between the Inhibition Constant (KI) and the Concentration of Inhibitor Which Causes 50 per Cent Inhibition (I50) of an Enzymatic Reaction. *Biochem Pharmacol* **1973**, *22* (23), 3099–3108. https://doi.org/10.1016/0006-2952(73)90196-2.

(8) Ellman, G. L.; Courtney, K. D.; Andres, V.; Featherstone, R. M. A New and Rapid Colorimetric Determination of Acetylcholinesterase Activity. *Biochem Pharmacol* **1961**, *7* (2), 88–95. https://doi.org/10.1016/0006-2952(61)90145-9.

(9) Leff, P.; Dougall, I. G. Further Concerns over Cheng-Prusoff Analysis. *Trends Pharmacol Sci* **1993**, *14* (4), 110–112. https://doi.org/10.1016/0165-6147(93)90080-4.

(10) Daina, A.; Michielin, O.; Zoete, V. SwissADME: A Free Web Tool to Evaluate Pharmacokinetics, Drug-Likeness and Medicinal Chemistry Friendliness of Small Molecules. *Scientific Reports 2017 7:1* **2017**, *7* (1), 1–13. https://doi.org/10.1038/srep42717.
